# Insight Into the Diversity and Possible Role of Plasmids in the Adaptation of Psychrotolerant and Metalotolerant *Arthrobacter* spp. to Extreme Antarctic Environments

**DOI:** 10.3389/fmicb.2018.03144

**Published:** 2018-12-18

**Authors:** Krzysztof Romaniuk, Piotr Golec, Lukasz Dziewit

**Affiliations:** Department of Bacterial Genetics, Faculty of Biology, Institute of Microbiology, University of Warsaw, Warsaw, Poland

**Keywords:** *Arthrobacter* spp., plasmid, Antarctica, psychrotolerant, metalotolerant, adaptation

## Abstract

*Arthrobacter* spp. are coryneform Gram-positive aerobic bacteria, belonging to the class *Actinobacteria*. Representatives of this genus have mainly been isolated from soil, mud, sludge or sewage, and are usually mesophiles. In recent years, the presence of *Arthrobacter* spp. was also confirmed in various extreme, including permanently cold, environments. In this study, 36 psychrotolerant and metalotolerant *Arthrobacter* strains isolated from petroleum-contaminated soil from the King George Island (Antarctica), were screened for the presence of plasmids. The identified replicons were thoroughly characterized in order to assess their diversity and role in the adaptation of *Arthrobacter* spp. to harsh Antarctic conditions. The screening process identified 11 different plasmids, ranging in size from 8.4 to 90.6 kb. A thorough genomic analysis of these replicons detected the presence of numerous genes encoding proteins that potentially perform roles in adaptive processes such as (i) protection against ultraviolet (UV) radiation, (ii) resistance to heavy metals, (iii) transport and metabolism of organic compounds, (iv) sulfur metabolism, and (v) protection against exogenous DNA. Moreover, 10 of the plasmids carry genetic modules enabling conjugal transfer, which may facilitate their spread among bacteria in Antarctic soil. In addition, transposable elements were identified within the analyzed plasmids. Some of these elements carry passenger genes, which suggests that these replicons may be actively changing, and novel genetic modules of adaptive value could be acquired by transposition events. A comparative genomic analysis of plasmids identified in this study and other available *Arthrobacter* plasmids was performed. This showed only limited similarities between plasmids of Antarctic *Arthrobacter* strains and replicons of other, mostly mesophilic, isolates. This indicates that the plasmids identified in this study are novel and unique replicons. In addition, a thorough meta-analysis of 247 plasmids of psychrotolerant bacteria was performed, revealing the important role of these replicons in the adaptation of their hosts to extreme environments.

## Introduction

Almost 80% of the Earth's biosphere is permanently cold, i.e., the temperature in these regions stays below 5°C throughout the whole year. The ocean depths constitute the majority of such environments, but there are also vast constantly cold terrestrial areas, such as the polar and alpine regions (De Maayer et al., 2014). Antarctica is known to be one of the coldest regions on Earth, with an average annual temperature ranging between −48°C at the South Pole and −2°C on island areas. It is an extreme habitat for indigenous organisms, not only because of the low temperatures, but also due to the occurrence of other harsh environmental conditions, including increased UV radiation, strong and drying winds, and the low amount of easily accessible nutrients (Martianov and Rakusa-Suszczewski, 1990; D'amico et al., 2006; Kejna et al., 2013; Cowan et al., 2014; Grzesiak et al., 2015; Kosek et al., 2017). Organisms living in Antarctica also have to face the emerging contamination of this region. Despite the fact that most of Antarctica is considered a pristine area with levels of contaminants that are lower than elsewhere in the world (these are mainly the consequence of natural bioweathering of heavy metal-rich rocks), there are areas where human influence is becoming more and more visible. Anthropogenic pollution by sulfur, heavy metals, polycyclic aromatic hydrocarbons and persistent organic pollutants (i.e., toxic organic compounds that are resistant to environmental degradation through chemical, biological, or photolytic processes, e.g., pesticides and pharmaceuticals) has been observed in Antarctica (Boutron and Wolff, 1989; Graf et al., 2010; Kukucka et al., 2010). Interestingly, the anthropogenic contaminants in this region mainly come from indigenous sources (e.g., ships and research stations). Intercontinental atmospheric long-range transport of pollutants to Antarctica seems to be highly limited, mostly due to the strong westerlies at mid-latitudes (40–60° S), which isolate the continent in winter (Kukucka et al., 2010; Stohl and Sodemann, 2010).

The extreme Antarctic environment leads to the selection of well-adapted organisms. These are poly-extremophiles that can withstand a variety of harsh physical and chemical conditions. In this study, we have focused on psychrotolerant, and at the same time metalotolerant, bacteria of the genus *Arthrobacter*, isolated from contaminated soil collected in the area of the Henryk Arctowski Polish Antarctic Station on King George Island (Romaniuk et al., 2018).

*Arthrobacter* spp. are Gram-positive, coryneform aerobic bacteria, belonging to the class *Actinobacteria*. They are frequently found in various extreme environments, including Arctic and Antarctic ice and soil (Dsouza et al., 2015; Singh et al., 2017), and also various contaminated sites (Sun et al., 2015; Ren et al., 2016; Chauhan et al., 2018). This wide distribution of *Arthrobacter* spp. could be a consequence of their high tolerance to various stress factors, such as permanent cold, long-term nutrient starvation, oxidative stress, osmotic pressure, and toxic compounds (Yao et al., 2015). *Arthrobacter* spp. can utilize a wide range of organic compounds that are recognized as environmental pollutants, including nitroglycerin, benzene derivatives, polycyclic aromatic hydrocarbons, haloalcohols, haloalkanes, *N*-heterocyclic compounds, insecticides and herbicides (Sharpee et al., 1973; Cripps, 1975; Pipke et al., 1987; Van Den Wijngaard et al., 1991; Jain et al., 1994; Casellas et al., 1997; Datta et al., 2000; Strong et al., 2002; Seo et al., 2006; Kallimanis et al., 2007; Rong et al., 2009; Husserl et al., 2010; Ren et al., 2016, 2018). In some cases, these abilities are conferred by plasmids, e.g., carbaryl (1-naphthyl *N*-methylcarbamate) pesticide utilization by *Arthrobacter* sp. RC1000 was linked with its plasmids pRC1 and pRC2 (Hayatsu et al., 1999), and a nicotine-utilization gene cluster was identified in plasmid pAO1 of *Arthrobacter nicotinovorans* (Baitsch et al., 2001). The wide distribution of *Arthrobacter* spp. and their ability to thrive in extreme environments indicates their great potential for environmental biotechnologies (Bjerketorp et al., 2018; Wang et al., 2018).

Currently (Nov. 11, 2018), 20 complete genomic and 33 plasmid sequences of *Arthrobacter* spp. are available in the GenBank database. The plasmids range in size from 3.7 to 754.2 kb, and they differ significantly in their gene content. Some carry genetic modules of adaptive and biotechnological value, e.g., the 113-kb linear plasmid pAL1 of *A. nitroguajacolicus* Rue61a, which confers the ability to utilize the *N*-heteroaromatic compound 2-methylquinoline (Overhage et al., 2005). A number of plasmids of mesophilic *Arthrobacter* spp. have been described in detail (Baitsch et al., 2001; Eaton, 2001; Igloi and Brandsch, 2003; Sandu et al., 2003; Overhage et al., 2005; Parschat et al., 2007; Jerke et al., 2008; Kolkenbrock and Fetzner, 2010; Kolkenbrock et al., 2010; Wagenknecht and Meinhardt, 2011; Mihasan and Brandsch, 2013, 2016; Mihasan et al., 2013; Mihasan, 2015). However, little is known about the genetic organization, biology and adaptive role of plasmids of Arctic and Antarctic, cold-active *Arthrobacter* spp. To our best knowledge, only one cryptic plasmid from a psychrotolerant *Arthrobacter* strain isolated from a Greenland ice core has been thoroughly described (Miteva et al., 2008).

Here, we report the detailed genomic analysis of 11 novel plasmids (named the ANT plasmids) identified within a pool of psychrotolerant and metalotolerant Antarctic *Arthrobacter* strains. Our analysis have revealed a putative role of the identified replicons in the adaptation of *Arthrobacter* spp. to the extreme Antarctic environment and gives an insight into the overall *Arthrobacter* plasmidome. What is more, this analysis complements previous works describing Antarctic plasmidomes, as in the majority they describe the ecological role of plasmids of Gram-negative bacteria (it was revealed that these replicons constitute more than 80% of all already analyzed plasmids of cold-active bacteria) (Dziewit and Bartosik, 2014; Ciok et al., 2018). Therefore, the knowledge on biology, evolution and diversity of plasmids of psychrotolerant Gram-positive bacteria is rather scarce. Therefore, thorough analyses of novel replicons of *Arthrobacter* spp. representing high GC content, Gram-positive genus give a broader view on the overall role of plasmids in bacteria inhabiting extreme polar environments and tend toward reducing the abovementioned bias in analyses of polar plasmidomes.

## Materials and Methods

### Bacterial Strains, Plasmids, and Culture Conditions

Thirty six *Arthrobacter* strains isolated from petroleum-contaminated soil collected in Antarctica were analyzed in this study (Romaniuk et al., 2018). Additionally, *Escherichia coli* DH5α was used (Hanahan, 1983). The strains were grown in LB or minimal salts medium M9 (Sambrook and Russell, 2001) (liquid or solidified with 1.5% agar) at 20°C (*Arthrobacter* spp.) and 37°C (*E. coli*). The media were supplemented with kanamycin (50 μg/ml), sodium pyruvate (0.4%) and laminarin (1%). Plasmids used and constructed in this study are listed in Table S1.

All *Arthrobacter* strains analyzed in this study are deposited at the microbiological repository of Institute of Microbiology, Faculty of Biology, University of Warsaw and are available for further analyses upon request.

### Standard Molecular Biology Techniques

Plasmid DNA was isolated using a standard alkaline lysis method (Birnboim and Doly, 1979). Routine DNA manipulations were carried out using standard methods (Sambrook and Russell, 2001). PCRs were performed using a KAPA HiFi PCR Kit and appropriate primer pairs in a Mastercycler (Eppendorf, Hamburg, Germany). The following program was used: a 3-min initial denaturation at 95°C, then 35 cycles of denaturation at 98°C for 20 s, annealing at 46–56°C (depending on the primer pair) for 15 s, and extension at 72°C for 30 s per kb, followed by a final extension at 72°C for 1 min per kb. Primers used in this study are listed in Table S1.

### DNA Sequencing

DNA sequencing was performed using an Illumina MiSeq instrument in paired-end mode using a v3 chemistry kit. The obtained sequence reads were filtered for quality and assembled using Newbler v3.0 software (Roche, Basel, Switzerland). Final gap closure was performed by capillary sequencing of PCR products using an ABI3730xl DNA Analyser (Applied Biosystems, Waltham, USA). The summary of the sequencing of particular plasmids was presented in Table S2.

### Bioinformatics

Plasmid sequences were manually annotated using Artemis software (Carver et al., 2012). Similarity searches were performed using the BLAST programs (Altschul et al., 1997) and Conserved Domains Database (Marchler-Bauer et al., 2017), provided by the NCBI (http://blast.ncbi.nlm.nih.gov/Blast.cgi) and Pfam (Finn et al., 2016). The detection of RNA sequences was performed using the ARAGORN v1.2.38 (Laslett and Canback, 2004) and tRNAscan-SE programs (Lowe and Chan, 2016). Helix-turn-helix motifs were identified using the helix-turn-helix DNA-binding motif prediction tool (Dodd and Egan, 1990). EC numbers were assigned using the KEGG database (Kanehisa et al., 2017) and UniProt Knowledgebase (UniProtKB) (Pundir et al., 2017). Restriction-modification systems were analyzed and classified using the REBASE database (Roberts et al., 2015). Insertion sequences were analyzed using ISfinder (Siguier et al., 2006). Comparative genomic analyses were performed using Circoletto (Darzentas, 2010) and BLASTp. The BLASTp search using a manually curated database of proteins encoded within the phenotypic modules of the ANT plasmids, was performed for investigation of the distribution of particular genetic modules in *Arthrobacter* genomes. For this analysis, the following cutoff values were applied (e-value < 1e-05 and amino acid sequence identity of at least 40%). Phylogenetic analyses were performed using the MEGA6 tool (Tamura et al., 2013) and trees were constructed using the Maximum Likelihood method based on the Tamura-Nei model (for the 16S rDNA tree) and Le and Gascuel model (for the ParA tree).

### Growth Kinetics

Growth curves were determined for bacteria cultivated in 2 ml lots of appropriate media in the glass tubes at 20°C (*Arthrobacter* spp.) or 37°C (*E. coli*) with shaking (150 rpm). The OD_600_ of the culture was measured every 24 h using SunriseTM plate reader (Tecan, Männedorf, Switzerland) with Magellan software (Tecan).

### Tellurium Resistance Testing

Analytical grade salt (Na_2_TeO_3_) was used in a resistance assay performed in 96-well plates, as described previously (Dziewit et al., 2013). Triplicate cultures of each strain were challenged with a range of concentrations of those heavy metal salts. Isolates that grew in the presence of 1 mM Te(IV) were considered resistant (Lewis et al., 2018).

### Direct-Plate UV Irradiation Assay

Responses to UV irradiation of various strains were compared by applying the modified direct-plate irradiation protocol (Lin and Wang, 2001). Bacterial cultures were grown overnight to about 1 x 10^8^ cells per ml. These cultures were plated on the LB plates and irradiated with the UVC lamp (Philips TUV−30-W-245 nm Lamp, type No. 57413-P/40) at a distance of 45 cm at exposure times (5, 10, 15, 18, 21, 24 s). Before each exposition, lids of the treatment plates were removed to avoid shielding. After irradiation, the lids were replaced and the plates were immediately placed in the incubator at 37°C for 48 h.

### Nucleotide Sequence Accession Numbers

Nucleotide sequences of the *Arthrobacter* plasmids were deposited in the GenBank (NCBI) database under the accession numbers MH067967-MH067977. Additionally, nucleotide sequences and information concerning insertion sequences and transposons were deposited in ISfinder (Siguier et al., 2006) and transposon registry (https://transposon.lstmed.ac.uk/) databases, respectively.

## Results and Discussion

### General Features of the ANT Plasmids

In a previous study, we isolated and characterized 90 psychrotolerant bacterial strains from petroleum-contaminated soil collected near the Henryk Arctowski Polish Antarctic Station on King George Island, Antarctica (62°09.601′ S, 58°28.464′ W) (Romaniuk et al., 2018). PCR amplification and sequencing of the 16S rRNA genes of these isolates followed by comparative sequence analysis allowed taxonomic assignment of all strains. The results showed that the main group of bacteria (36 isolates, named the ANT strains in this work) belong to the *Arthrobacter* genus (Romaniuk et al., 2018). This observation corroborates previous reports that *Arthrobacter* spp. are among the most frequently isolated cultivable soil bacteria, even in polar regions, e.g., they comprised 14.7% of cultivable bacteria isolated from soil samples collected at the Browning Peninsula, Eastern Antarctica and 22% of isolates originating from postglacial soils of Ecology Glacier, King George Island, Antarctica (Zdanowski et al., 2013; Pudasaini et al., 2017). Also the results of various metagenomic analyses suggest that *Arthrobacter* spp. are amongst most frequent bacterial genera in soil from polar regions as they were identified in >3 clone-library-based or 454-based studies (as it was revealed from the summarized literature data) (Pearce et al., 2017). Moreover, metagenomic analyses of other soils worldwide also indicate that *Arthrobacter* spp. often constitute a dominant or subdominant group, e.g., in rhizosphere microbiome of burned holm-oak in the Sierra Nevada Natural and National Park (SE Spain) they comprised more than 21% of the total bacterial community, while in the permafrost from the Qinghai-Tibet Plateau *Arthrobacter* spp. comprised up to 15% of all bacteria (Hu et al., 2016; Fernandez-Gonzalez et al., 2017).

**Figure 1:**
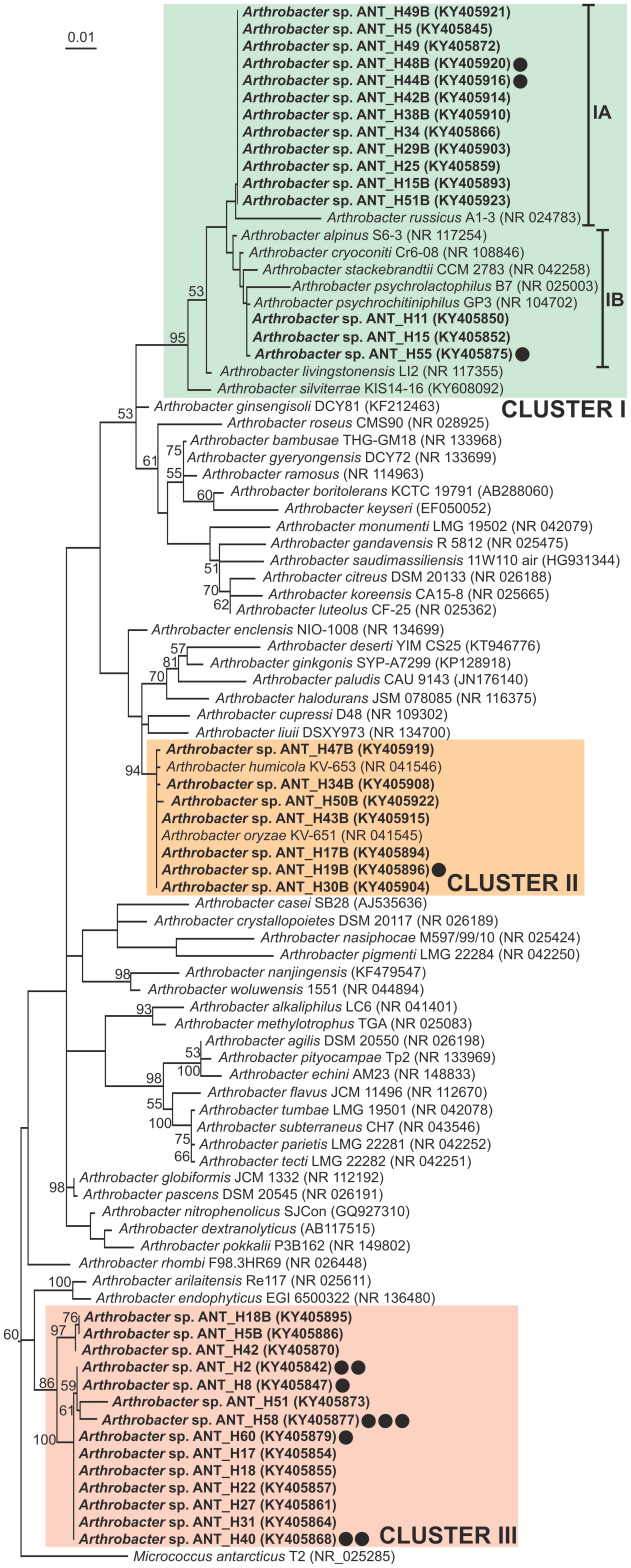
Phylogenetic tree for 16S rDNA sequences of *Arthrobacter* spp. The tree was constructed by applying the Maximum Likelihood method based on the Tamura-Nei model. Statistical support for the internal nodes was determined by 1,000 bootstrap replicates and values of ≥50% are shown. The tree is drawn to scale, with branch lengths measured in the number of substitutions per site. The analysis involved 91 nucleotide sequences. All positions containing gaps and missing data were eliminated. There were a total of 1,121 positions in the final dataset. The 16S rDNA sequence of *Micrococcus antarcticus* T2 was used as an outgroup. GenBank accession numbers of the 16S rDNA sequences used for the phylogenetic analysis are given in brackets. The strains analyzed in this study are in bold text. Clusters I–III, distinguished by colored backgrounds, contain the ANT strains. The IA and IB subclusters are within the cluster I. Strains containing plasmids were indicated by black dots. Number of dots corresponds to the number of plasmids found within the particular strain.

Phylogenetic analysis using partial 16S rDNA sequences (1,206 nt) of 36 ANT strains and 54 type strains representing various *Arthrobacter* species retrieved from the NCBI database was performed. The tree topology revealed that the ANT strains form three separate clusters, which strongly suggests their divergence, although they were all isolated from the same soil sample (Figure 1). The levels of identity of 16S rDNA sequences within each cluster are high. Cluster I groups 23 strains, including 15 ANT strains. Within this cluster two subclusters were distinguished: IA grouping 12 ANT strains together with a reference strain *Arthrobacter russicus* A1-3, isolated from an air sample from the Russian space station Mir (exhibiting reciprocal identity of 16S rDNA sequences between 97.8 and 100%) (Li et al., 2004), and IB clustering three ANT strains showing the highest identity level (99.8%) with the sequence of *Arthrobacter psychrochitiniphilus* GP3, isolated from Adélie penguin guano from Antarctica (Wang et al., 2009). Cluster II groups seven ANT isolates and two reference strains, i.e., *Arthrobacter oryzae* KV-651 and *Arthrobacter humicola* KV-653, isolated from a paddy soil sample collected in Japan (Kageyama et al., 2008). These strains exhibit very high reciprocal identity (between 99.8 and 100%) of their 16S rDNA sequences. The remaining 14 ANT strains form cluster III gathering strains with 16S rRNA genes identical in 98 to 100%. An outlier for this cluster is *Arthrobacter arilaitensis* Re117, sharing between 97.2 and 97.6% 16S rDNA identity with the ANT strains grouped within cluster III (Irlinger et al., 2005) (Figure 1).

Plasmids, as mobile extrachromosomal elements, may potentially contribute to bacterial adaptation to various environmental conditions occurring in diverse geographical locations, including Antarctica (Dziewit and Bartosik, 2014). The *Arthrobacter* strains analyzed in this study were isolated from a multi-extreme environment that is not only permanently cold, but is also highly contaminated with heavy metals and toxic organic compounds (Romaniuk et al., 2018). Therefore, it is possible that plasmids of bacteria originating from this environment carry various genetic modules of adaptive value. To identify and characterize the plasmidome of Antarctic *Arthrobacter* spp., all of the aforementioned ANT strains were screened for extrachromosomal replicons. This analysis revealed the presence of 13 plasmids (named the ANT plasmids) within nine strains (six carry a single plasmid, two have two plasmids, and one strain has three plasmids). Based on analysis of NCBI database resources, we noticed that plasmid-containing *Arthrobacter* spp. usually carry one to three extrachromosomal replicons, although a recent report describes *Arthrobacter* sp. ZXY-2, a strain hosting five plasmids (Zhao et al., 2017).

The complete nucleotide sequences of the 13 ANT plasmids were determined and thoroughly analyzed. All of these plasmids are circular replicons ranging in size from 8.4 to 90.6 kb, with a high GC content of between 57.4 and 63.7%. Our analysis revealed the presence of 11 unique plasmid sequences, since the smallest of the plasmids, pA2H1 (8,447 bp), was found in three different strains: ANT_H2, ANT_H55, and ANT_H60. Interestingly, the second plasmid in the ANT_H2 strain, pA2H2, was not present in either ANT_H55 or ANT_H60 (Table 1). The presence of the same plasmid in three strains suggests that horizontal gene transfer occurs in Antarctic soil. It is also noteworthy that two nearly identical plasmids, pA44BH1 and pA48BH1, differing by only 10 bp, were found in two different ANT strains (Table 1).

**Table 1:** General features of the ANT plasmids.

**Plasmid name**	**GenBank accession number**	**Strain**	**Plasmid size (bp)**	**GC content (%)**	**Number of genes**	**% of coding regions**	**Genetic modules^a^**
pA2H1	MH067968	ANT_H2	8,447	57.4	9	74.8	MOB, REP, TNP
		ANT_H55				
		ANT_H60				
pA2H2	MH067969	ANT_H2	64,347	61.8	60	82.6	EFX, LIP, PAR, TER, TNP, TRA, UMU, XER
pA8H1	MH067977	ANT_H8	43,280	60.8	46	80.3	LAM, PAR, RM, SLP, SUL, TER, UMU, XER
pA19BH1	MH067967	ANT_H19B	69,439	63.7	54	86.1	ARM, EFX, PAR, TNP, TRA
pA40H1	MH067970	ANT_H40	78,287	59.0	71	84.7	COP, PAR, RM, TA, TNP, TRA
pA40H2	MH067971	ANT_H40	90,618	59.1	89	83.0	CYT, PAR, RM, SLP, SUL, TER, TNP, TRA, UMU, XER
pA44BH1	MH067972	ANT_H44B	40,676	59.3	36	86.9	PAR, RE, TNP, TRA
pA48BH1	MH067973	ANT_H48B	40,674	59.3	37	86.9	PAR, RE, TNP, TRA
pA58H1	MH067974	ANT_H58	33,641	57.7	31	84.9	PAR, RM, TRA
pA58H2	MH067975	ANT_H58	73,138	60.4	73	87.0	CYT, PAR, RM, SUL, TNP, TRA
pA58H3	MH067976	ANT_H58	84,207	59.8	78	83.1	ARM, MFS, PAR, RM, TAU, TCT, TNP, TRA, UMU

a *ARM, aromatic compound utilization; COP, copper resistance; CYT, cytochrome biogenesis; EFX, drug-specific efflux system; LAM, laminarinase; LIP, lipoprotein export; MFS, major facilitator transporter; MOB, mobilization for conjugal transfer; PAR, partitioning; RE, type IV restriction enzyme; REP, replication; RM, restriction-modification; TA, toxin-antitoxin; TNP, transposition; SLP, sulfate transport; SUL, sulfur metabolism; TAU, taurine transport; TCT, tricarboxylate transport; TER, tellurium resistance; TRA, conjugal transfer; UMU, UV-damage protection/repair; XER, recombination*.

**Figure 2:**
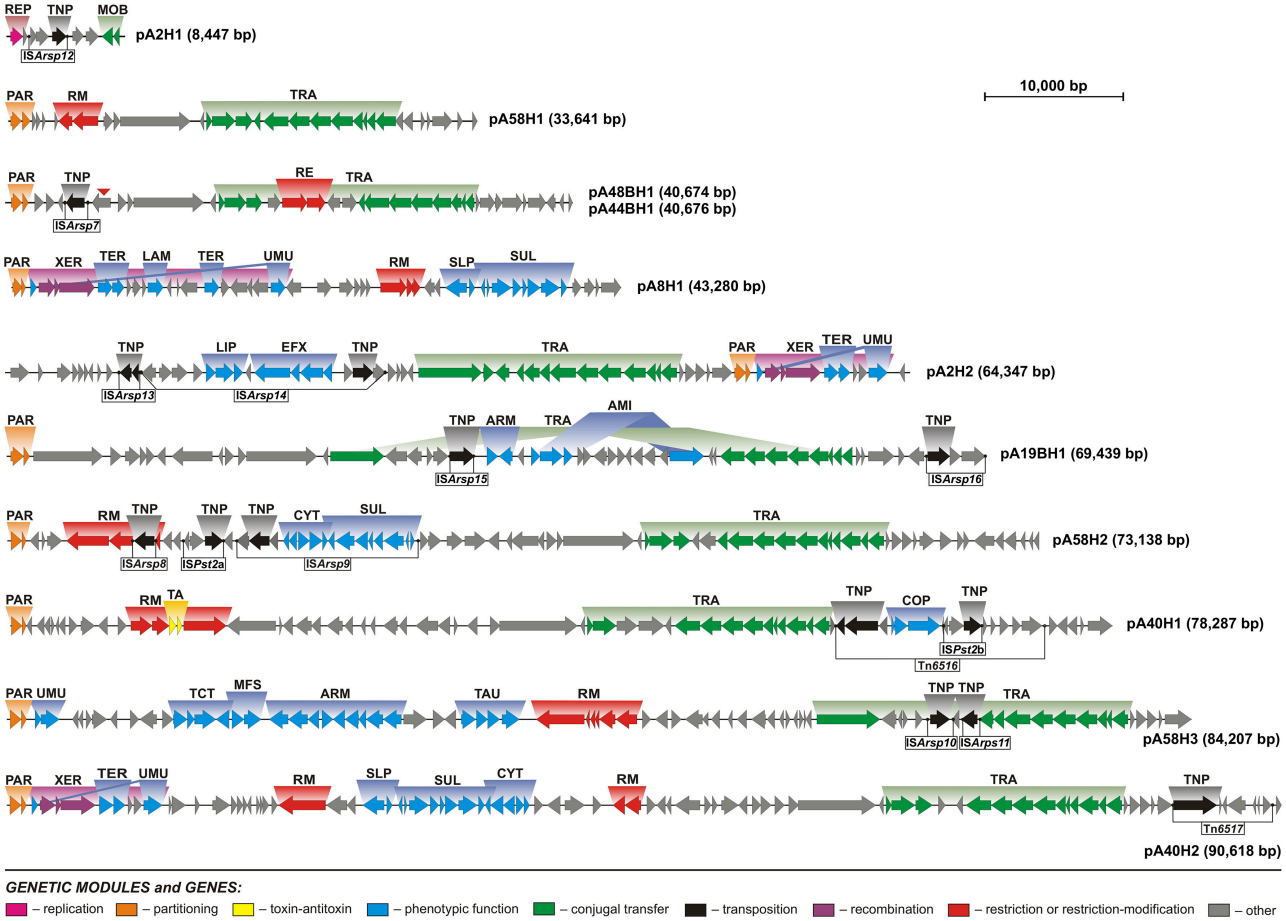
Linear maps showing the genetic structure and organization of the circular plasmids of Antarctic *Arthrobacter* spp. Arrows indicate genes and their transcriptional orientation. Predicted genetic modules are indicated by colored boxes: ARM, aromatic compound utilization; COP, copper resistance; CYT, cytochrome biogenesis; EFX, drug-specific efflux; LAM, laminarinase; LIP, lipoprotein export system; MFS, major facilitator transporter; MOB, mobilization for conjugal transfer; PAR, partitioning; RE, type IV restriction enzyme; REP, replication; RM, restriction-modification; TA, toxin-antitoxin; TNP, transposition; SLP, sulfate transport; SUL, sulfur metabolism; TAU, taurine transport; TCT, tricarboxylate transport; TER, tellurium resistance; TRA, conjugal transfer; UMU, UV-damage protection/repair; XER, recombination. Potentially active transposable elements were distinguished and the location of their IR sequences is marked by black dots. A red triangle indicates the gene that was disrupted in the plasmid pA44BH1, but is intact within pA48BH1.

After manual annotation, 582 predicted genes were distinguished in the sequenced plasmid genomes and putative biological functions were assigned to 56% of them (Figure 2, Table S3). The smallest (pA2H1) and largest (pA40H2) ANT plasmids, carry 9 and 89 predicted genes, respectively. In comparison, the smallest *Arthrobacter* plasmid known until now, pAG001 (3.7 kb) of *Arthrobacter* sp. IHBB 11108, contains 6 genes (Kiran et al., 2015), while the largest, unnamed2 (754.2 kb), isolated from strain ERGS1:01, carries 675 genes (Kumar et al., 2015).

### Genetic Modules Comprising the ANT Plasmid Backbones

Bacterial plasmids have a modular structure and it is possible to dissect them into several functional genetic modules. The backbone is comprised of a set of gene clusters, coding for replication, stabilization and conjugal transfer functions, which are necessary for plasmid maintenance and spread (Toussaint and Merlin, 2002). Comprehensive analyses of the *Arthrobacter* plasmid sequences allowed us to distinguish and characterize the genetic modules comprising their functional backbones (Figure 2).

### Replication and Stabilization Modules

Detailed genomic analyses of the ANT plasmids led to the identification of a recognizable replication system only in the replicon pA2H1. This module is similar to a previously described replication system of the plasmid pPRH of *Arthrobacter rhombi* PRH1 (Stanislauskiene et al., 2012). It is composed of two overlapping genes, *pA2H1_01* and *pA2H1_02* (Figure 2, Table S3). Analysis of the putative polypeptide encoded by *pA2H1_01* using BLASTp identified the closest homolog as a hypothetical protein of *Glutamicibacter* sp. BW77 (acc. no. WP_096254262) (91% amino acid sequence identity), containing replicase (pfam03090) and primase C-terminal 1 (PriCT_1) domains characteristic for replication proteins. Analysis of the protein encoded by *pA2H1_02* revealed the presence of two helix-turn-helix motifs (RTARELAQKTGLSERTIRAWTA, residues 11–32 and LSMRAISKEVGIAVSAVHYALQ, residues 57–78), which suggests that this protein can interact with DNA.

Attempts to identify replication systems within the sequences of the other ANT plasmids were unsuccessful. A previous study of *Arthrobacter* plasmids also concluded that the identification of replication systems by *in silico* analysis is probably not possible based on our current knowledge (Mihasan, 2015).

Plasmids, as extrachromosomal elements that are usually not crucial for cell viability, have to carry their own survival kit, i.e., a stabilization system that helps to maintain the replicon in a bacterial cell. This could be especially important in the meaning of bacteria inhabiting extreme environments, where a general tendency to lose extrachromosomal elements may be even stronger comparing with more favorable environmental conditions. Therefore, analyzing the role of plasmids in adaptation, it is important to understand the mechanisms enabling their stable maintenance in bacterial cell. There are two main groups of plasmid-stabilizing modules: (i) partitioning systems (PAR) that promote the active segregation of plasmids into daughter cells during cell division, and (ii) toxin-antitoxin systems (TA) that cause post-segregational elimination of plasmid-less cells (Thomas, 2000; Salje, 2010; Harms et al., 2018).

Analyses of the ANT plasmids revealed that 10 of them carry partitioning systems composed of two genes encoding ParA (ATPase) and ParB (DNA-binding protein), that may potentially interact with a predicted *cis*-located partitioning site, *parS* (Table S4). The only exception is the smallest identified plasmid pA2H1, which lacks a recognizable stabilization system. Based on a comparative analysis of ParA protein sequences, Mihăşan proposed that *Arthrobacter* plasmids should be divided into 4 clades (Mihasan, 2015). In the present study, we have extended this classification by adding all currently available *Arthrobacter* plasmid-encoded ParA sequences.

**Figure 3:**
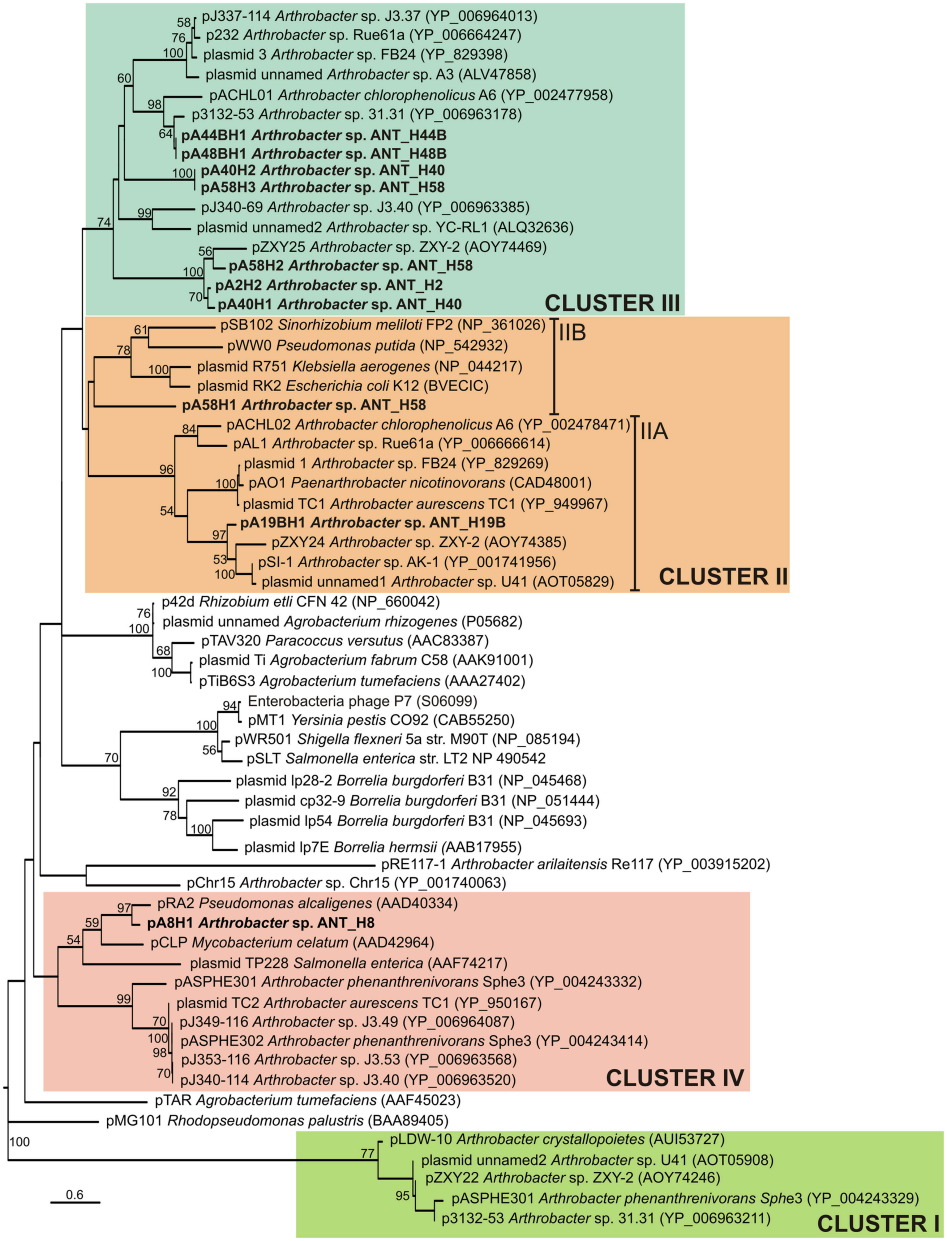
Phylogenetic tree of partitioning proteins (ParAs) encoded within the *Arthrobacter* plasmids. The analysis was based on 39 sequences of the ParA proteins (122 amino acid positions) encoded within the *Arthrobacter* plasmids. Additionally, 23 reference ParA sequences of other bacteria, previously used by Mihasan (2015), were included in this analysis. The unrooted tree was constructed using the Maximum Likelihood method based on the Le and Gascuel model (Le and Gascuel, 2008). The tree is drawn to scale, with branch lengths measured in the number of substitutions per site. Statistical support for the internal nodes was determined by 1,000 bootstrap replicates and values of ≥50% are shown. Accession numbers of the protein sequences used for the analysis are given in brackets. Clusters I–IV group ParA proteins of *Arthrobacter* plasmids, with reference to previously proposed clustering (Mihasan, 2015). The names of plasmids analyzed in this work are in bold text.

Phylogenetic analysis was performed using 62 plasmid-encoded ParA protein sequences (122 amino acid positions), including 9 ParAs originating from the ANT plasmids and 53 sequences used previously (Mihasan, 2015), or identified by us in searches of the NCBI database. The resulting phylogenetic tree topology revealed that the ParA proteins encoded by the ANT plasmids group within three separate clusters (Figure 3). The seven ParAs of plasmids pA2H2, pA41H1, pA40H2, pA44BH1, pA48BH1, pA58H2, and pA58H3 were grouped together with nine other *Arthrobacter* spp. plasmid ParAs previously classified within cluster III (Mihasan, 2015) (Figure 3). The ParA proteins of pA19BH1 and pA58H1 were classified within cluster II (Mihasan, 2015) (Figure 3). However, it was possible to distinguish two subclusters within this grouping. The first (IIA) includes the ParA of pA19BH1 and sequences from *Arthrobacter* spp. and closely related *Paenarthrobacter nicotinovorans* ATCC 49919 (Igloi and Brandsch, 2003), while the second (IIB) contains the ParA of pA58H1 and proteins originating from the plasmids RK2 of *E. coli* (Easter et al., 1998), R751 of *Klebsiella aerogenes* (Thorsted et al., 1998), pWW0 of *Pseudomonas putida* (Greated et al., 2002), and pSB102 of *Sinorhizobium meliloti* FP2 (Schneiker et al., 2001) (Figure 3). The ParA protein of pA8H1 was grouped within cluster IV. Interestingly, this ParA formed a separate subcluster together with three plasmids of diverse origin, i.e., pRA2 of *Pseudomonas alcaligenes* NCIB 9867 (Kwong et al., 2000), pCLP of *Mycobacterium celatum* (Le Dantec et al., 2001), and plasmid TP228 of *Salmonella enterica* subsp. enterica serovar Newport (Hayes, 2000) (Figure 3).

A stabilizing module of the second type, i.e., a toxin-antitoxin system, was found only within plasmid pA40H1 (Table S3). This is composed of two small non-overlapping genes with the putative antitoxin gene downstream of that encoding the toxin. *In silico* analyses showed that it is a *tad-ata*-like, type II TA system (Dziewit et al., 2007). The predicted toxin displays homology to proteins of the RelE/ParE superfamily, while the putative antitoxin belongs to the HTH_XRE family of transcriptional regulators (Dziewit et al., 2007). This observation suggests that TA systems are rare within *Arthrobacter* plasmids compared to PAR systems, and they probably play a minor role as stabilizing modules.

### Conjugal Transfer Modules

Plasmids can be transferred between bacterial cells via conjugation, a complex process involving pilus synthesis, aggregate stabilization, surface exclusion and DNA metabolism (Llosa et al., 2002). Some plasmids carry only a minimal set of genetic elements (i.e., usually an *oriT* sequence and a gene encoding a relaxase that initiates the conjugal transfer by nicking the DNA at *oriT*) enabling their conjugal transfer. Such replicons are called mobilizable plasmids, and their transfer depends on the presence of other self-transmissible elements in the bacterial cell (Smillie et al., 2010). The presence of the conjugal transfer systems within plasmids is important from the evolutionary and ecological point of view as it enables dissemination of adaptive features between bacteria inhabiting particular environment. Therefore, because of a central role in prokaryote adaptation and evolution, studying genetic diversity, biology, and biochemistry of plasmid transfer systems in various groups of bacteria (including extremophilic ones) is currently under active investigation (Pistorio et al., 2008).

Genomic analysis of the ANT plasmids revealed that all but one (pA8H1), carry between 2 and 13 genes encoding proteins potentially involved in conjugal transfer (Figure 2, Table S3). Based on comparative analyses we predict that nine ANT plasmids (pA2H2, pA19BH1, pA40H1, pA40H2, pA44BH1, pA48BH1, pA58H1, pA58H2, and pA58H3) are self-transmissible, i.e., conjugative replicons, while pA2H1 is a mobilizable plasmid. Genes encoding putative relaxases were identified and the subsequent analysis of conserved sequence motifs in these enzymes allowed their classification into two families: MOB_F_ and MOB_P_ (Garcillan-Barcia et al., 2009).

Relaxases (TraAs) encoded within plasmids pA2H2, pA58H3, and pA19BH1 belong to the MOB_F_ family. The TraAs of pA2H2 and pA58H3 share 94% amino acid sequence identity, and the conjugal transfer modules of these two plasmids are highly similar. However, this gene cluster is disrupted by the insertion of two transposable elements in pA58H3 (Figure 2), and this may influence the efficiency of the plasmid conjugation process. A homologous conjugal transfer module was previously identified in plasmid pZXY24 (acc. no. CP017425) of *Arthrobacter* sp. ZXY-2, originating from a pesticide plant in China. The last plasmid, pA19BH1, encodes a relaxase sharing only 39% amino acid sequence identity with the TraAs of pA2H2 and pA58H3. Interestingly, the conjugal transfer module of this replicon seems to be divided (and possibly inactivated) by the insertion of a large (24-kb) DNA fragment.

The relaxases of plasmids pA40H1, pA40H2, pA44BH1, pA48BH1, pA58H1, and pA58H2 were classified into the MOB_P_ family. BLASTn searches revealed that gene clusters found within these plasmids that encode components of a type IV secretion system involved in bacterial conjugation are related to several other conjugal transfer systems found within various *Arthrobacter* plasmids (Mihasan and Brandsch, 2016). The presence of conserved genes encoding proteins involved in plasmid conjugation, gene synteny and the occurrence of similar DNA repeats (previously identified within conjugal transfer modules of *Arthrobacter* spp. by Mihăşan and Brandsch) suggests that many *Arthrobacter* plasmids may be related and could have evolved from a common ancestor (Mihasan and Brandsch, 2016). Interestingly, the predicted relaxase of the mobilizable plasmid pA2H1 also belongs to the MOB_P_ family. This plasmid carries two non-overlapping genes encoding a predicted relaxase (MobA) and a mobilization protein (MobC). BLASTp analysis revealed the presence of a homologous mobilization module within plasmid KLBMP5180 (acc. no. CP012751) of *Glutamicibacter halophytocola* KLBMP 5180, isolated from the inner tissues of the halophyte plant *Limonium sinense* in China.

### Phenotypic Modules of the ANT Plasmids

Besides the genes comprising replicon backbones, many plasmids also carry auxiliary genes that determine phenotypes that may be beneficial under particular environmental conditions, e.g., antibiotic and heavy metal resistance, and the utilization of complex organic compounds (Toussaint and Merlin, 2002). Analysis of the auxiliary genetic load of the ANT plasmids revealed that seven of these replicons carry gene clusters that may have adaptive value, i.e., they might contribute to the survival of their hosts in the extreme polar environment. Phenotypic modules recognized in these plasmids are possibly involved in (i) protection against ultraviolet (UV) radiation, (ii) resistance to heavy metals, (iii) transport and metabolism of organic compounds, (iv) sulfur metabolism, and (v) protection against exogenous DNA (Table S5).

### Protection Against UV Radiation

Due to stratospheric ozone depletion, Antarctica is a region where the level of UV radiation is higher than in other areas (Madronich, 1994). UV-B exposure may cause DNA damage and increase production of reactive oxygen species (ROS), thus inducing mutagenesis and leading to genomic instability (Santos et al., 2012). The *umuCD* DNA repair system is a component of the global SOS system and its expression is regulated by the transcriptional repressor LexA (Butala et al., 2009). In response to DNA damage (e.g., caused by UV radiation), *umuCD*-encoded DNA polymerase of the Y family, enables DNA synthesis past lesions in the template strand. Therefore, the *umuCD* genes play an important role in maintaining genome stability in bacteria (Goodman et al., 2016; Murison et al., 2017). Genomic analysis revealed that these DNA repair systems are often found within plasmids of cold-active bacteria, especially those originating from polar regions (Dziewit and Bartosik, 2014).

UMU modules (comprised of *umuCD*) were identified within four of the analyzed ANT plasmids: pA2H2, pA8H1, pA40H2, and pA58H3 (Table S5). However, in the first three replicons these modules were disrupted by the insertion of DNA fragments between the *umuC* and *umuD* genes. These insertions (XER modules) may in fact represent integrative elements of a novel type. They contain (i) conserved core regions, composed of three genes encoding a predicted tyrosine recombinase XerD, a transcriptional regulator of the XRE family, and site-specific recombinase XerC, as well as (ii) variable regions, composed of 4 or 14 genes. Interestingly, the variable regions contain genetic modules of potential adaptive value, e.g., a tellurium resistance module and a gene encoding a putative laminarinase (Figure 2). We hypothesize that the conserved core regions of these elements are responsible for DNA rearrangements, while the variable regions constitute an auxiliary genetic load. The elements inserted within the *umuCD* modules of plasmids pA2H2 and pA40H2 are 7.5 kb in size and exhibit 99% nucleotide sequence identity. The element found in pA8H1 is larger (16.6 kb) and contains a DNA region present within two other XER modules plus an additional DNA fragment.

For testing the predicted biological function of the UMU module carried by the pA58H3 plasmid, it was PCR amplified and cloned within the pBBR1 MCS-2 vector. Comparing the UV tolerance of *E. coil* carrying obtained pBBR-UMU and “empty” vector revealed lack of differences. Based on the obtained results we think that the tested genetic module is inactive in *E. coli*.

### Resistance to Heavy Metals

In a previous study, we performed a chemical analysis of soil samples collected near the Arctowski Polar Station, which revealed high concentrations of chromium, copper, mercury, nickel, and zinc. Moreover, we showed that bacteria isolated from these samples were multi-resistant and carried various heavy metal resistance genes. In particular, the ANT strains were resistant to the following heavy metals: As(V)−34 strains (MIC values between 25 and 700 mM), Cu(II)−34 (2–8 mM), As(III)−32 (3–18 mM), Ni(II)−20 (2–4 mM), Zn(II)−7 (2–3 mM), Cr(VI)−4 (2 mM), and Co(II)−2 (2 mM) (Romaniuk et al., 2018). In the present study, we tested for the presence of heavy metal resistance genes within the *Arthrobacter* plasmids. Four heavy metal resistance modules were detected by *in silico* analysis of these replicons (Table S5).

Three putative tellurium resistance modules (TER) were found in plasmids pA2H2, pA8H1, and pA40H2 (Figure 2, Table S5). Tellurium is toxic to most living organisms and its toxicity is related to the generation of ROS and disturbance of the thiol:redox buffering system. Previously, it was shown that tellurium-resistant bacteria are common in Antarctic soils (Arenas et al., 2014; Rodriguez-Rojas et al., 2016a,b; Munoz-Villagran et al., 2018; Valdivia-Gonzalez et al., 2018). Our analysis revealed that the tellurium resistance modules of the ANT plasmids are composed of two overlapping genes encoding tellurium resistance protein TerC and a hypothetical protein that probably operates as a cation-transporting ATPase. This module is present in the three aforementioned plasmids and also within the genome of *Paeniglutamicibacter gangotriensis* Lz1yT, a bacterium isolated from a penguin rookery soil sample collected in Antarctica (Shivaji et al., 2013).

We tested whether the TER modules carried by the *Arthrobacter* plasmids confer resistance to Te(IV). The MIC values were determined to establish the level of tellurium resistance of the *Arthrobacter* plasmid-bearing strains. All analyzed strains were recognized as sensitive and the MIC value for all them was 1 mM. All identified TER modules are nearly identical, thus only one of them was used for further analyses. The TER module of the pA40H2 plasmid was amplified by PCR and cloned within the pBBR1 MCS-2 vector for testing its activity in the heterological host—*E. coli*. Unfortunately, it was found to be inactive in this host.

The fourth heavy metal resistance module (COP) was found in plasmid pA40H1, as a passenger gene cluster of the transposon Tn*6516*. COP is predicted to be involved in copper resistance. Cu is an essential trace element for all bacteria, functioning as a cofactor of various enzymes. However, an excess of Cu is toxic. Under aerobic conditions, Cu is thought to catalyze the production of hydroxyl radicals, via the Fenton and Haber-Weiss reactions, that may cause oxidative damage to various cellular macromolecules. Under anaerobic conditions an excess of copper leads to the formation of adventitious Cu(I)-thiolate bonds, which inactivates enzymes whose activity depends on free cysteines or disulfide bonds. In addition, copper in excess can lead to incorrect disulfide bond formation in the periplasm (Ladomersky and Petris, 2015). The analyses performed in our previous study revealed that the concentration (elemental composition) of copper in soil collected near the Arctowski station was 126 μg g^−1^, i.e., up to 7 times higher than at other sites on King George Island (Romaniuk et al., 2018). These concentration is above the acceptable norms for Cu content in various soils and sediments, including agricultural soils in European Union (Kabata-Pendias and Pendias, 2001; Hooda et al., 2010; Toth et al., 2016). Moreover, we found that 94% of *Arthrobacter* strains isolated from this region were resistant to Cu(II) (Romaniuk et al., 2018). However, the identification of only one copper resistance module within the ANT plasmids, suggests that there are other chromosomal Cu-resistance genes.

The COP module of the pA40H1 plasmid is composed of three genes encoding (i) copper chaperone CopZ (COG2608) which functions as a heavy metal transporter, (ii) a protein containing a heavy metal-binding domain, and (iii) a copper-translocating P-type ATPase (COG2217) (Lu et al., 2003; Singleton et al., 2009) (Figure 2, Table S5). These proteins exhibit between 97 and 100% amino acid sequence identity with homologous proteins (acc. nos. WP_047117894-WP_047117894) of *Arthrobacter* sp. YC-RL1, an aromatic compound-degrading bacterium isolated from polluted agricultural soil in China (Ren et al., 2016).

### Transport and Metabolism of Organic Compounds

Lichens and bryophytes are abundant in Antarctica (Colesie et al., 2018). These organisms accumulate a storage glucan in their cells called moss starch, which is composed of glucose units linked by β-1,3 and β-1,4 glycosidic bonds. One of the enzymes that enables bacteria to use moss starch as a carbon source is laminarinase. Interestingly, the same enzyme may be used by marine bacteria for the utilization of the algal storage glucan laminarin (Davis, 1992; Alderkamp et al., 2007). *In silico* analyses of the plasmid pA8H1 identified a predicted gene (*pA8H1_p12*) encoding a glycoside hydrolase family protein. Detailed analysis of this pA8H1-encoded protein led to the identification of a LamG domain, that is characteristic of laminarinases, responsible for the hydrolysis of 1,3-beta-D-glucosidic bonds in glucans such as laminarins or curdlans. The presence of a predicted laminarinase (LAM) module in pA8H1, suggested that the strain ANT_H8 may be able to utilize moss starch as an alternative carbon and/or energy source. This was tested in a series of growth experiments. It was shown that none of analyzed *Arthrobacter* strains is able to use laminarin as a sole carbon source for growth. Additionally, the LAM module was tested in a heterological host. This analysis revealed that *E. coli* carrying pBBR-LAM plasmid was not able to utilize laminarin. This may suggest that the module is not functional or needs some specific (yet not known) environmental factors for its activation. Alternatively, we may also speculate that this enzyme is responsible for utilization of a glucose polimer, other than laminarin.

Analysis of the plasmid pA58H3 revealed the presence of a large gene cluster containing four genetic modules potentially involved in the transport and metabolism of organic compounds. The modules encode (i) a tricarboxylate transport system, (ii) an ABC-type nitrate/sulfonate/bicarbonate transport system, (iii) major facilitator transporters, and (iv) enzymes potentially involved in aromatic compound utilization (Figure 2, Table S5).

The putative tricarboxylate transport system (TCT module) of pA58H3 is composed of 4 genes encoding homologs of the TctA, TctB, and TctC proteins, as well as a LysR family transcriptional regulator. This gene cluster is homologous to a genetic module of *Arthrobacter* sp. Leaf337 (Bai et al., 2015). It was proposed that this type of module may be involved in citrate transport (Sweet et al., 1979). Also present within pA58H3 are genes encoding an ABC-type nitrate/sulfonate/bicarbonate transport system, which may be involved in taurine transport (TAU module). Taurine is widely distributed in animal tissues. Excess taurine produced by animals is excreted either directly in urine, or as taurocholate in bile (Sekowska et al., 2000; Schuller-Levis and Park, 2003). While plants and animals cannot degrade taurine, various bacteria and fungi are capable of doing this. They can use taurine as the sole source of carbon, nitrogen, sulfur and energy (Shimamoto and Berk, 1979). In *Arthrobacter* spp. taurine most probably supports growth when utilized as the sole sulfur source, since sulfite formation by the action of taurine dioxygenase was confirmed for *Arthrobacter* sp. Rue61a (Niewerth et al., 2012). It is also noteworthy that taurine can protect bacteria from osmotic stress, and thus it could be an important protective agent under extreme environmental conditions (Mosier et al., 2013).

A gene encoding a predicted major facilitator transporter (MFS module) was also found in plasmid pA58H3. The pA58H3_p18 transporter shares >50% amino acid sequence identity with several aromatic acid, vanillate and benzoate transporters of *Rhodococcus* spp. In addition, it contains a conserved TIGR00895 domain that is shared by members of the cl26865 superfamily, grouping transporters of benzoate, carbohydrates, organic alcohols or acids. Therefore, we speculate that the activity of this transporter may be linked with the function of the fourth genetic module within the aforementioned gene cluster of pA58H3, that contains genes potentially involved in the utilization of aromatic compounds such as phenoxybenzoates (ARM module). This module contains genes encoding enzymes involved in the transformation of aromatic compounds: (i) aldehyde dehydrogenase (pA58H3_p20), belonging to a large family of NAD(P)^+^-dependent enzymes enabling the oxidation of endogenous and exogenous aliphatic and aromatic aldehydes to their corresponding carboxylic acids, and playing an important role in detoxification (Sophos and Vasiliou, 2003); (ii) 3-ketoacyl-(acyl-carrier-protein) reductase (pA58H3_p21), belonging to the SDR superfamily of enzymes catalyzing the metabolism of steroids, cofactors, carbohydrates, lipids, aromatic compounds, amino acids, and acting in redox sensing (Oppermann et al., 2003); (iii) phthalate dioxygenase reductase (pA58H3_p24), an FMN-dependent reductase mediating electron transfer from the two-electron donor reduced nicotinamide adenine nucleotide (NADH), to the one-electron acceptor, [2Fe-2S] (Correll et al., 1992; Gassner et al., 1995); (iv) Zn-dependent alcohol dehydrogenase (pA58H3_p26), that may catalyze the NAD(P)(H)-dependent interconversion of alcohols to aldehydes or ketones (Persson et al., 2008); and (v) phthalate 4,5-dioxygenase (pA58H3_p27), belonging to a large class of aromatic ring-hydroxylating dioxygenases that enable microorganisms to tolerate and utilize aromatic compounds for growth (Tarasev and Ballou, 2005).

Genes encoding proteins potentially involved in the utilization of aromatic compounds were also found in plasmid pA19BH1: (i) *pA19BH1_p21* encoding a predicted 3-ketoacyl-(acyl-carrier-protein) reductase belonging to the SDR superfamily; and (ii) *pA19BH1_p22* encoding a putative class III extradiol dioxygenase which uses a non-heme Fe(II) to cleave aromatic rings between a hydroxylated carbon and an adjacent non-hydroxylated carbon (Broderick, 1999).

The presence of genes encoding enzymes potentially involved in aromatic compound utilization within plasmids pA53H3 and pA19BH1 may be strictly linked to the presence of pollutants in the petroleum-contaminated soil sample from which the *Arthrobacter* strains were isolated. This soil sample was collected near the petroleum pumping and storage warehouse at the Henryk Arctowski Polish Antarctic Station (Romaniuk et al., 2018).

It is also important to mention that within the transposable element IS*Arsp14*, carried by plasmid pA2H2, two predicted efflux systems (LIP and EFX) were found. The putative ABC-type lipoprotein export system, namely LIP, is composed of three genes encoding (i) an ATPase (COG1136) homologous to LolD, (ii) a permease of the FtsX-like family (COG0577)—the LolC homolog, and (iii) a transcriptional regulator of the TetR family. This efflux system is similar to LolCDE, found in gram-negative bacteria (Zuckert, 2014). The second module EFX is composed of four genes encoding (i) a predicted transporter of the MMPL family, most probably involved in lipid transport, (ii) a putative drug efflux protein of EmrB/QacA subfamily, and (iii) two TetR family transcriptional regulators. Interestingly, a homologous genetic module was also found within plasmid pA19BH1 (Figure 2). It is possible that the EFX module is a drug-specific efflux system (Lomovskaya and Lewis, 1992; Saier et al., 1998).

### Sulfur Metabolism

Sulfur oxidation is an important and well-characterized process of chemolithotrophic bacteria. It may also be used by heterotrophic bacteria for detoxication and probably energy generation (Liu et al., 2014). Glutathione spontaneously reacts with elemental sulfur to generate glutathione persulfide, and this compound may be oxidized by the enzyme sulfur dioxygenase (EC 1.13.11.18) to form sulfite and glutathione. It has been proposed that this reaction is not only a detoxification process, but it may also generate ATP via oxidative phosphorylation (Liu et al., 2014).

Analysis of the ANT plasmids revealed that three of them (pA8H1, pA40H2, and pA58H2) contain gene clusters that are probably linked to sulfur metabolism (SUL module). These clusters are nearly identical (≥97% nucleotide sequence identity) and each of them contains a gene encoding a putative sulfur dioxygenase. Seven other proteins are encoded by these SUL modules: (i) a CsoR family transcriptional regulator, (ii) a small hypothetical protein of unknown function, (iii and iv) two rhodanese-related sulfurtransferases (COG0607), (v) a TauE/SafE sulfite exporter (Weinitschke et al., 2007), (vi) a transporter of the major facilitator superfamily, and (vii) a DUF302 domain-containing hypothetical protein (Figure 2, Table S5). Plasmids pA8H1 and pA40H2 also contain an additional genetic module (named SLP) composed of two genes encoding a MerR family transcriptional regulator and sulfate permease (SulP) involved in sulfate uptake.

Furthermore, plasmids pA40H2 and pA58H2, in close proximity to the aforementioned SUL modules, contain nearly identical (97% nucleotide sequence identity) five-gene clusters (named CYT) encoding proteins required for cytochrome biogenesis or related to sulfur metabolism: (i) a transcriptional regulator (COG3682), (ii and iii) two cytochrome c-type biogenesis proteins CcdA (DsbD analogs, COG0785), (iv) a thiol-disulfide oxireductase (DsbA family protein, COG1651), and (v) intracellular sulfur oxidation protein (DsrE/DsrF family, COG1416) (Figure 2, Table S5). It is hypothesized that the combined activities of the CYT, SUL and SLP modules may be responsible for energy gain from the oxidation of sulfur compounds.

### Protection Against Exogenous DNA

Bacteria have developed several mechanisms to protect their cells against invading exogenous DNA (mostly bacteriophages). The most common of these are restriction-modification (RM) systems that allow discrimination between foreign (non-methylated) DNA invading the bacterial cell and endogenous (methylated) genetic material. As a consequence, the restriction endonuclease destroys exogenous DNA. RM systems have been classified into four major types according to subunit composition, recognition site, cofactor requirements and cleavage position (Tock and Dryden, 2005; Vasu and Nagaraja, 2013).

The biological role of the RM systems is to protect the host against (i) infecting bacteriophages, which would otherwise convert the cell into a factory to replicate phage particles and either kill it or compromise its health, or (ii) other selfish DNA (including plasmids), which may consume cellular energy and substrates for their own maintenance, thus affecting cell growth (Tock and Dryden, 2005). The prevention of energy and substrate loss is especially important for bacteria inhabiting nutrient-poor and extreme environments like Antarctica (De Maayer et al., 2014). Moreover, plasmid-borne RM systems may function as a mechanism of post-segregational cell killing that increases the stability of the plasmid in the bacterial population (Dziewit et al., 2011; Mruk and Kobayashi, 2014; Werbowy et al., 2015; Sitaraman, 2016).

Within the analyzed ANT plasmids we found seven putative RM modules (Figure 2, Table 2). Four systems, identified in plasmids pA8H1, pA40H1, pA58H1, and pA58H3, were classified as type I RM modules. They are composed of three genes encoding a restriction endonuclease, DNA methyltransferase and the S subunit determining the specificity of both restriction and methylation (Table 2) (Vasu and Nagaraja, 2013). Two other systems, carried by pA40H2, represent type II RMs (Table 2). One belongs to the IIG subtype, and encodes a single polypeptide with both cleavage (endonuclease) and modification domains (Pingoud et al., 2005). The second represents the IIP subtype and encodes two proteins: a DNA m^5^C methyltransferase and a restriction endonuclease with the probable recognition sequence 5′-GGCGCC-3′ (predicted on the basis of detailed comparative sequence analysis performed at the REBASE database) (Roberts et al., 2015). The last RM system represents an example of type III and is present within pA58H2. This module is most probably inactive due to the insertion of the transposable element IS*Arsp8* (Figure 2).

**Table 2:** Restriction-modification systems and genes encoding type IV restriction enzymes identified within the ANT plasmids.

**Plasmid name**	**Genes for restriction or restriction-modification systems (coordinates)**	**Type^a^**	**Best BLASTp hit for the restriction endonuclease (GenBank acc. no.)**
pA8H1	*pA8H1_p28*-*p30* (26,211–29,034)	I	Restriction endonuclease of *Paenibacillus sabinae* (WP_025334610)
pA40H1	*pA40H1_p13*-*p14* and *pA40H1_p17* (8,581–15,359)	I	Restriction endonuclease of *Arthrobacter crystallopoietes* (WP_005269194)
pA58H1	*pA58H1_p07*-*p08* (3,622–6,419)	I	Restriction endonuclease of *Gordonia* sp. 852002-10350_SCH5691597 (WP_064861386)
pA58H3	*pA58H3_p35*-*p40* (37,630–44,790)	I	Restriction endonuclease subunit R of *Arthrobacter* sp. Leaf69 (KQN86732)
pA40H2	*pA40H2_p24* (19,320–22,664)	IIG	Hypothetical protein of *Arthrobacter* sp. RIT-PI-e (WP_082177329)
pA40H2	(*pA40H2_p46*-*p47*) (43,092–45,084)	IIP	Nuclease of *Arthrobacter* sp. AK-YN10 (ERI35594)
pA58H2	(*pA58H2*_*p06*-*p07* and *pA58H2_p09*) (4,195–10,824)	III	Type III restriction-modification system endonuclease of *Actinotignum timonense* (WP_087070271)
pA44BH1	(*pA44BH1_p16*-*p17*) (19,883–22,958)	IV	Hypothetical protein of *Microbacterium* sp. Cr-K29 [WP_029260290]
pA48BH2	(*pA48BH1_p17*-*p18*) (19,871–22,946)	IV	Hypothetical protein of *Microbacterium* sp. Cr-K29 [WP_029260290]

a *Classification based on comparison with the REBASE database*.

In addition, within plasmids pA44BH1 and pA48BH1, we detected identical pairs of genes (*mcrB*-like and *mcrC*-like) encoding a type IV McrBC-like methylation-dependent restriction enzyme. McrB and McrC are both necessary for methyl-directed restriction activity (Raleigh, 1992).

### Distribution of the Phenotypic Modules of the ANT Plasmids in *Arthrobacter* Genomes

To evaluate the distribution of 14 phenotypic modules carried by the ANT plasmids in various *Arthrobacter* genomes a BLAST search using 20 chromosomes and 33 plasmids of *Arthrobacter* spp. retrieved from the NCBI database was performed (Table S6). It revealed that within the chromosomes of analyzed *Arthrobacter* spp. there are between 2 and 10 genetic modules, homologous to the gene clusters of the ANT plasmids described above. It was also shown that such modules are not common within the plasmids of *Arthrobacter* spp., as they were found only in nine replicons. Amongst these plasmids, the pLDW-10 replicon originating from *A. crystallopoietes* DSM 20117, isolated from the soil sample collected in USA, contains as many as four modules (ARM, CYT, SUL, and TER), while other plasmids contain only one or two such modules (Table S6).

Further analysis revealed that the LAM and TAU gene clusters are specific for the ANT plasmids, as they were found in any of the analyzed *Arthrobacter* genomes. Interestingly both modules are involved in metabolism of alternative carbon sources (putatively, laminarin, and taurine), which may be extremely beneficial for the host under nutrient limitation. In contrast, COP, SLP, TER, and UMU modules were found in majority of *Arthrobacter* chromosomes, and additionally TER and UMU modules were also recognized within analyzed *Arthrobacter* plasmids (including: unnamed plasmid of *A. alpinus* A3, pAL1 plasmid of *Arthrobacter* sp. Rue61a, and unnamed plasmid 2 of *Arthrobacter* sp. U41) (Table S6).

### Transposable Elements Found Within the ANT Plasmids

Transposable elements (TEs) are common in prokaryotic genomes and play an important role in the evolution of bacteria. They are responsible for various DNA rearrangements, including duplications, insertions, inversions, and deletions (Mahillon and Chandler, 1998). A thorough analysis of the ANT plasmids enabled the identification of 14 TEs, including two transposons of the Tn*3* family and 12 insertion sequences (IS) of the IS*1380*, IS*3*, IS*30*, IS*481*, IS*As1*, IS*NCY*, and IS*L3* families (Table 3).

**Table 3:** Transposable elements found within the ANT plasmids.

**Transposable element**	**Family/Group**	**Plasmid**	**Number of passenger genes**	**Size (bp)**	**IR sequence^a^**	**DR sequence^a^**
IS*Arsp7*	IS*L3*	pA44BH1 pA48BH1	0	1,451	IRL: GGCTCTTCGCGGTTGGTGGTGAAT	CCTTTGCC
					IRR: GGCTCTTCGCGTTTCGTGGTGAAT
IS*Arsp8*	IS*481*	pA58H2	0	1,718	IRL:TGTGATCTCTCAGAACATCGTTTACAACATCACTCAGGACATCGCTTACA	TGCTGCAAATGCCGA
					IRR:TGTGAACACTCGCTTCTTTGTTTACAGTCGCACTCACTACATCGTTTACA
IS*Arsp9*	IS*NCY*	pA58H2	11	12,978	IRL:TGTGAGCAACAACGATAAGTGGCCTGTTTTTCAC	AAGGATGCTC
					IRR:TGTGAATCGCAACGGTAAGTGGCGTGTTTTTCGC
IS*Arsp10*	IS*1380*	pA58H3	0	1,667	IRL: CCTTGATCCACCGAT	CTTAG
					IRR: CCTTGATCCACCGAT
IS*Arsp11*	IS*As1*	pA58H3	0	1,306	IRL: CAGGGCTGATGCAAAGTCG	GGAACAGCAC
					IRR: CAGGGCTAATGCAAAGTCG
IS*Arsp12*	IS*481*	pA2H1	2	2,615	IRL: TGTACTGACCGGACACGTTG	CCTAGA
					IRR: TGTACTGACCGGACACGTTG
IS*Arsp13*	IS*3*/ IS*150*	pA2H2	0	1,442	IRL: TGAACTGCTCCCCGAAAGTTGGACT	TCC
					IRR: TGAACTGGTCCCCGAAAGTTGGACT
IS*Arsp14*	IS*NCY*	pA2H2	12	17,535	IRL: CTGTGAATCGCAGCGGTAAATGGCGTGTTTTTTGCAGCG	GCTTCTT
					IRR: CTGTGAATCGCAACGGTAAATGGCGTGTTTTTCGCAGCG
IS*Arsp15*	IS*30*	pA19BH1	0	1,862	IRL: GGATTCTATTGATCGAAGCAACG	GACCCACGCG
					IRR: GGATTTCAGTGGTCGACGCAACG
IS*Arsp16*	IS*NCY*	pA19BH1	2	4,338	IRL: TGAGAATGCTCATTGGTATGTCAGTAA	GTT
					IRR: TGAGAATGCTCATCGAGATGTCAGTAA
IS*Pst2*a	IS*L3*	pA58H2	2	2,985	IRL: GGGTATCCGGAATTTCTGGTTGAT	TTTTTAAA
					IRR: GGGTATACGGATTTAATGGTTGAT
IS*Pst2*b	IS*L3*	pA40H1	2	2,985	IRL: GGGTATCCGGAATTTCTGGTTGAT	CAAAAAAG
					IRR: GGGTATACGGATTTAATGGTTGAT
Tn*6516*	Tn*3*	pA40H1	12	14,887	IRL: GGGGTGGCTTGTGAAAAAGTGAAATATCCGACGCTAAG	TCTAT
					IRR: GGGGTGGCTTGTGAAAAAGTGAAATATCCGACGCTAAG
Tn*6517*	Tn*3*	pA40H2	6	7,080	IRL: GGGGTCTGAGCTGCAGCGGAACAGTGGTTGGCGCTAAG	TGGTA
					IRR: GGGGTCTGAACTGCAGCGGAACAGTGGTTGGCGCTAAG

a *Inverted and direct repeats are shown in the 5′ to 3′ orientation*.

#### Transposons of the Tn3 Family

The characteristic feature of Tn*3* family transposons is their modular structure. Three types of functional genetic module can be distinguished within these elements: (i) a transposase gene, (ii) a cointegrate resolution module, and (iii) passenger genes (Toussaint and Merlin, 2002; Nicolas et al., 2015). Analysis of the ANT plasmids identified two novel Tn*3*-family transposons, Tn*6516* and Tn*6517*, within the plasmids pA40H1 and pA40H2, respectively (Figure 2, Table 3).

The Tn*6516* transposon (14,887 bp) contains 15 genes, including the core genetic module encoding transposase and resolvase, as well as passenger genes (Figure 2, Table 3). The auxiliary genes of Tn*6516* include the copper resistance module (COP) described above. Interestingly, an additional TE, IS*Pst2*b of the IS*L3* family, was detected downstream of the COP module. The IS*Pst2*b element is composed of 3 genes encoding a transposase, putative permease (COG0701) and an ArsR family transcriptional regulator. The remaining passenger genes of Tn*6516* encode four hypothetical proteins, an FrmR family transcriptional regulator and a putative alkylhydroperoxidase family enzyme (COG2128).

The second Tn*3* family transposon, Tn*6517*, is of the size 7,080 bp. It carries transposase and serine resolvase genes, and five passenger genes encoding hypothetical proteins (Figure 2, Table 3).

#### Insertion Sequences

*In silico* analysis of the ANT plasmids revealed the presence of 12 insertion sequences. Six of them are typical ISs carrying only transposase genes: IS*Arsp7* (IS*L3* family), IS*Arsp8* (IS*481* family), IS*Arsp10* (IS*1380* family), IS*Arsp11* (IS*As1* family), IS*Arsp13* (IS*3* family/IS*150* group), and IS*Arsp15* (IS*30* family). Other ISs found within the ANT plasmids carry between 2 and 12 passenger genes (Figure 2, Table 3).

IS*Arsp12* (2,615 bp) belonging to the IS*481* family, identified within the smallest ANT plasmid pA2H1, carries 2 passenger genes, encoding an ArsR family transcriptional regulator and a predicted permease of unknown specificity (Figure 2, Table 3). This is highly similar (86% nucleotide sequence identity) to IS*Pfr21* of *Propionibacterium freudenreichii* (Falentin et al., 2010).

The two 2,985-bp long elements, IS*Pst2*a (of pA58H2) and IS*Pst2*b (of pA40H1) of the IS*L3* family, also carry 2 passenger genes encoding an ArsR family transcriptional regulator and putative permease (Figure 2, Table 3). It is important to mention that these auxiliary genes share no similarity with those of IS*Arsp12*. These two ISs differ in only 2 bp and are isoforms of IS*Pst2* that is present in several copies in the genomes of three *Pseudomonas stutzeri* strains (Bolognese et al., 1999).

In addition, three IS*NCY* family ISs were found within the ANT plasmids: IS*Arsp9* (of pA58H2), IS*Arsp14* (of pA2H2), and IS*Arsp16* (of pA19BH1). These elements all carry passenger genes. IS*Arsp9* (12,978 bp) contains 11 passenger genes, which comprise the CYT and SUL modules described above. IS*Arsp14* (17,535 bp) encodes a transposase, resolvase, and ATP-binding protein showing a high level of similarity to the equivalent proteins of IS*Arsp9* and IS*Bli29* of *Brevibacterium linens* BS258 (acc. no. CP014869). Seven of the 12 passenger genes of IS*Arsp14* comprise the predicted transport system modules LIP and EFX, described above. The third identified element of the IS*NCY* family, IS*Arsp16*, is much smaller (4,338 bp). It encodes a transposase showing 69% amino acid sequence identity to the transposase of IS*Aau5* found within plasmid TC1 of *Arthrobacter aurescens* TC1 (Mongodin et al., 2006). IS*Arsp16* carries two passenger genes encoding hypothetical proteins of unknown function (Figure 2, Table 3).

### Comparative Analysis of *Arthrobacter* Plasmids and Meta-Analysis of Plasmids of Psychrotolerant Bacteria

Looking for tracks of possible horizontal gene transfer and common or distinct origin of the ANT replicons their comparative genomic analysis was performed. It was based on all against all BLASTn comparison. Similar approach was applied previously in several other analyses of bacterial plasmids, e.g., extrachromosomal replicons of *Streptococcus macedonicus* and *Lactococcus lactis* (Gorecki et al., 2011; Papadimitriou et al., 2015). Performed analysis of the ANT plasmids revealed that there are several DNA regions shared by these replicons. This is particularly apparent for plasmids pA40H2, pA8H1, and pA2H2, that possess nearly identical XER modules (96% nucleotide sequence identity), and plasmids pA40H2 and pA58H2, which carry almost identical CYT and SUL modules (97% identity) (Figure S1). This may reflect possible horizontal transfer of genetic modules between these replicons. On the other hand, lack of conservation of plasmids' backbones may suggest that the ANT replicons originate from various ancestors.

Comparison of the nucleotide sequences of the ANT plasmids and other *Arthrobacter* plasmids (mostly from mesophilic strains) revealed their divergence, since only limited similarity (usually confined to the conjugal transfer modules) was found. This indicates that the ANT plasmids are novel and unique replicons. Interestingly, the highest level of similarity detected is between pA8H1 and the unnamed plasmid (acc. no. CP013298) of *Arthrobacter* sp. YC-RL1, which possess nearly identical modules predicted to participate in sulfur metabolism (97% nucleotide sequence identity) (Figure S1).

The proteomes of *Arthrobacter* plasmids were then examined by an all against all comparative protein analysis. This confirmed the results of the nucleotide sequence comparisons showing that the ANT plasmids share many more homologous proteins with each other, than with the plasmids of mesophilic *Arthrobacter* spp. The only exceptions were the nearly identical plasmids pA44BH1 and pA48BH1, that encode as many as 19 (>50%) proteins with homology to those of p3132-53 of *Arthrobacter* sp. 31-32 (acc. no. JQ418520), isolated from aromatic hydrocarbon-contaminated soil from Indiana, USA. A more detailed analysis of the possible functions of putative proteins common to various *Arthrobacter* plasmids revealed that they are mostly involved in conjugal transfer or replicon stabilization. Therefore, they are products of the conserved genetic backbones of the plasmids, whereas proteins encoded by auxiliary genes are mostly unique. This finding seems to support the hypothesis that many *Arthrobacter* plasmids may be related and have a common ancestor (Mihasan and Brandsch, 2016), and in the course of evolution they have gained various auxiliary genes that may confer an adaptive advantage to their hosts in their specific habitats.

The auxiliary genetic information stored in all sequenced so far *Arthrobacter* plasmids was carefully inspected (Table 4). It was shown that seven (43.75%) out of 16 plasmids carry genetic modules encoding proteins involved in metabolism and transport of organic compounds. Interestingly, four plasmids (all belonging to the group of ANT replicons) carry genes enabling protection against UV radiation and resistance to heavy metals. It was also shown that *Arthrobacter* plasmids are rich of restriction-modification systems, as they were found within nine (56.25%) plasmids (Table 4).

**Table 4:** Functions of auxiliary genetic modules identified within *Arthrobacter* plasmids.

**Function**		**Plasmid name/GenBank accession number^a^/Strain name**
Protection against:	Low temperature	Plasmid unnamed1/CP015733/*Arthrobacter* sp. U41
	Oxidative stress	Plasmid unnamed3/CP015735/*Arthrobacter* sp. U41
	UV radiation	pA2H2**/MH067969/***Arthrobacter* sp. ANT_H2 pA40H2**/MH067971/***Arthrobacter* sp. ANT_H40 pA58H3**/MH067976/***Arthrobacter* sp. ANT_H58 pA8H1**/MH067977/***Arthrobacter* sp. ANT_H8
Resistance to heavy metals		pA2H2**/MH067969/***Arthrobacter* sp. ANT_H2 pA40H1**/MH067970/***Arthrobacter* sp. ANT_H40 pA40H2**/MH067971/***Arthrobacter* sp. ANT_H40 pA8H1**/MH067977/***Arthrobacter* sp. ANT_H8
Protection against exogenous DNA—restriction-modification system		Plasmid unnamed1/CP015733 *Arthrobacter* sp. U41 pA19BH1/**MH067967**/*Arthrobacter* sp. ANT_H19B pA40H1/**MH067970**/*Arthrobacter* sp. ANT_H40 pA40H2/**MH067971**/*Arthrobacter* sp. ANT_H40 pA44BH1/**MH067972**/*Arthrobacter* sp. ANT_H44B pA48BH1/**MH067973**/*Arthrobacter* sp. ANT_H48B pA58H1/**MH067974**/*Arthrobacter* sp. ANT_H58 pA58H3/**MH067976**/*Arthrobacter* sp. ANT_H58 pA8H1/**MH067977**/*Arthrobacter* sp. ANT_H8
Metabolism and transport of:	Carbohydrates	pA19BH1/**MH067967**/*Arthrobacter* sp. ANT_H19B pA2H2**/MH067969/***Arthrobacter* sp. ANT_H2 pA40H1/**MH067970**/*Arthrobacter* sp. ANT_H40 pA40H2/**MH067971**/*Arthrobacter* sp. ANT_H40 pA58H2/**MH067975**/*Arthrobacter* sp. ANT_H58 pA58H3/**MH067976**/*Arthrobacter* sp. ANT_H58 pA8H1/**MH067977**/*Arthrobacter* sp. ANT_H8
	Amino acids	pA40H1/**MH067970**/*Arthrobacter* sp. ANT_H40 pA58H2/**MH067975**/*Arthrobacter* sp. ANT_H58 pA58H3/**MH067976**/*Arthrobacter* sp. ANT_H58
	Nucleotides	pA58H2/**MH067975**/*Arthrobacter* sp. ANT_H58
	Lipids	pA19BH1/**MH067967**/*Arthrobacter* sp. ANT_H19B pA2H2**/MH067969/***Arthrobacter* sp. ANT_H2
	Inorganic ions	Plasmid unnamed1/CP015733 *Arthrobacter* sp. U41 pA40H2/**MH067971**/*Arthrobacter* sp. ANT_H40 pA58H2/**MH067975**/*Arthrobacter* sp. ANT_H58 pA58H3/**MH067976**/*Arthrobacter* sp. ANT_H58 pA8H1/**MH067977**/*Arthrobacter* sp. ANT_H8
	Toxic organic compounds	pA19BH1/**MH067967**/*Arthrobacter* sp. ANT_H19B pA58H3/**MH067976**/*Arthrobacter* sp. ANT_H58
Energy production and conversion		pA19BH1/**MH067967**/*Arthrobacter* sp. ANT_H19B pA40H2/**MH067971**/*Arthrobacter* sp. ANT_H40 pA58H2/**MH067975**/*Arthrobacter* sp. ANT_H58 pA58H3/**MH067976**/*Arthrobacter* sp. ANT_H58

a *GenBank accession numbers of plasmids identified and analyzed in this study are in bold text*.

For a broader view of the possible role of the ANT plasmids in the adaptation of extremophilic *Arthrobacter* spp., a meta-analysis of 247 plasmids of psychrotolerant bacteria representing 36 genera was performed. This analysis revealed that the most common feature found within plasmids of bacteria from cold regions is the presence of genes conferring resistance to heavy metals (Figure S2, Table S7). It was shown that 11.74% of the analyzed plasmids carry such genes. In the ANT plasmids, heavy metal resistance genes were found in four replicons (Figure S2, Table S7). The abundance of heavy metal resistance genes and their presence within mobile genetic elements (i.e., plasmids) is understandable because these toxic elements are the most commonly occurring bactericidal factors in nature (Nies, 1999). It is important to mention that heavy metals are not only anthropogenic contaminants, they also occur naturally in minerals and are released during (bio)weathering processes (Gadd, 2007; Matlakowska et al., 2012).

Our meta-analysis also revealed that within the plasmids of psychrotolerant bacteria, and especially those isolated from Arctic and Antarctic regions, genes conferring protection against low temperature, oxidative stress and UV radiation are very common. Amongst the analyzed plasmids 18.22% carry such genes (Figure S2, Table S7). In polar regions, low temperature is the predominant life-limiting factor (D'amico et al., 2006). In addition, the increased UV radiation and oxygen solubility favor the formation of ROS, which may cause damage to DNA, RNA, proteins, and lipids (Cabiscol et al., 2000; Casanueva et al., 2010). Therefore, the acquisition (on a plasmid) of genes conferring protection against these stressors could be highly beneficial for the bacterial host.

We also performed a meta-analysis of the distribution (within the plasmids of psychrotolerant bacteria) of genes whose products are involved in the transport and metabolism of carbohydrates, amino acids, nucleotides, lipids, inorganic ions, and toxic organic compounds, as well as energy production and conversion (Figure S3, Table S7). This revealed that 17% of the analyzed plasmids carry such genes (Figure S3, Table S7). The abundance of metabolic genes within plasmids of psychrotolerant bacteria may represent a specific form of adaptation, since cold-active microorganisms usually inhabit nutrient-poor environments, where genes enabling the utilization of poorly-degradable compounds are highly beneficial.

## Conclusions

In this study, we explored the plasmidome of Antarctic *Arthrobacter* strains isolated from soil samples collected at the Henryk Arctowski Polish Antarctic Station on King George Island. In total, 11 various plasmids were identified, sequenced and analyzed. These replicons exhibit great diversity in both size and nucleotide sequence. The plasmids carry from 9 to 89 predicted genes, some forming genetic modules that potentially benefit their bacterial hosts. A comparative analysis of nucleotide sequences allowed us to define the ANT plasmids as novel and unique replicons in the *Arthrobacter* genus.

The analysis of the ANT plasmid backbones revealed that most of them contain partitioning systems. This allowed ParA-based phylogenetic classification of all *Arthrobacter* plasmids. In addition, despite previously reported difficulties in identifying (with the use of bioinformatic tools) replication modules in *Arthrobacter* plasmids, we were able to distinguish one such system in the plasmid pA2H1. Moreover, based on *in silico* analysis, the majority of the ANT plasmids were recognized as self-transmissible replicons.

The bioinformatic analyses that we performed indicated the presence of genetic modules that may be responsible for adaptation of the bacteria to the extreme Antarctic environment. The following genetic modules of predicted adaptive value were distinguished: (i) *umuCD* genes (UMU module) conferring protection against UV radiation, (ii) tellurium and copper resistance determinants (TER and COP), (iii) genes encoding laminarinase (LAM), several enzymes involved in aromatic compound utilization (ARM) and various transporters (EFX, LIP, MFS, TAU, and TCT), that may be involved in the transport and utilization of alternative carbon sources, and (iv) sulfur metabolism genes (CYT, SLP, and SUL).

It was also revealed that the ANT plasmids contain a number of restriction or restriction-modification systems whose function could be protection against exogenous DNAs, such as bacteriophages, or alternatively some may act as plasmid stabilization modules. Moreover, 14 novel transposable elements, including two transposons of the Tn*3* family and 12 insertion sequences of the IS*1380*, IS*3*, IS*30*, IS*481*, IS*As1*, IS*NCY*, and IS*L3* families, were identified within the ANT plasmids. Interestingly, our analysis also revealed the presence of putative novel integrative elements (XER modules) that contain a conserved core region composed of three genes encoding a predicted tyrosine recombinase XerD, transcriptional regulator of the XRE family and site-specific recombinase XerC, accompanied by 4 or 14 passenger genes.

It is important to mention that the presented work has also several limitations, that one should be aware of. First of all, the biological function of the distinguished genetic modules is based only on bioinformatic analyses, and although the investigations were very complex, these predictions are still speculative. Therefore, there is a need for experimental validation of biological functions of the predicted genetic modules. However, this needs a development of a proper host-vector systems for analyses of *Arthrobacter* spp. The majority of the *Arthrobacter* genes characterized so far have been cloned and expressed in *E. coli*. However, heterologous expression, can lead to production of inactive proteins due to misfolding, a lack of appropriate enzyme cofactors and proper insertion of these cofactors into apoenzymes, or various posttranslational modifications. Such approach was presented in this study and we hypothesize that inability of showing the functionality of selected *Arthrobacter* modules in the heterological host may be in fact a consequence of abovementioned reasons.

As a limitation of this study we can also recognize lack of full genomic sequences of all analyzed *Arthrobacter* strain, which forecloses testing if the plasmid modules confer novel and unique biological functions or genes responsible for the particular process are already present within the host's chromosome. Other limitation of this work is that although 36 strains were analyzed this is still a relatively small pool of bacteria. For more complex analyses of a larger pool of bacteria, a joint-collaboration approach involving various scientific centers working with Antarctic prokaryotes could be proposed. An alternative is also a shotgun metagenomic approach for discovering a total metaplasmidome of Antarctic soil samples, and this is an aim of our current works.

In summary, our analysis of the ANT plasmids, supported by a meta-analysis of 247 plasmids of various psychrotolerant bacteria, shows that extrachromosomal replicons may play an important role in the adaptation of *Arthrobacter* strains (and bacteria in general) to the extreme Antarctic environment.

## Author Contributions

LD and KR conceived and designed experiments. KR sequenced and annotated plasmids. KR, PG, and LD analyzed the data. LD contributed reagents, materials, and analysis tools. KR, PG, and LD wrote manuscript. All authors read and approved the submitted version.

### Conflict of Interest Statement

The authors declare that the research was conducted in the absence of any commercial or financial relationships that could be construed as a potential conflict of interest.
