# Supplementary material for: Insight Into the Diversity and Possible Role of Plasmids in the Adaptation of Psychrotolerant and Metalotolerant Arthrobacter spp. to Extreme Antarctic Environments
Source: Front Microbiol. 2018 Dec 18;9:3144. doi: 10.3389/fmicb.2018.03144 (PMC6305408; doi:10.3389/fmicb.2018.03144)
Supplement: Supplementary file 1 [file Table_1.pdf]

Supplementary Material

Insight into the Diversity and Possible Role of Plasmids in the Adaptation of Psychrotolerant and Metalotolerant *Arthrobacter* spp. to Extreme Antarctic Environments

Krzysztof Romaniuk, Piotr Golec, Lukasz Dziewit*
*** Correspondence:** Dr. Lukasz Dziewit: ldziewit@biol.uw.edu.pl

TABLE S1. Plasmids and PCR primers used in this study.

Plasmid	Characteristics	Reference or source
pBBR1 MCS-2	Km^r; 5.1 kb; *ori* pBBR1; Mob^+; *oriT* RK2; *lacZα*; MCS	(Kovach et al., 1994)
pBBR-LAM	Km^r; 6.9 kb; pBBR1 MCS-2 derivative carrying PCR-amplified LAM module of the pA8H1 plasmid cloned within SalI site	This study
pBBR-TER	Km^r; 7.8 kb; pBBR1 MCS-2 derivative carrying PCR-amplified TER module of the pA40H2 plasmid cloned within SacI site	This study
pBBR-UMU	Km^r; 9.3 kb; pBBR1 MCS-2 derivative carrying PCR-amplified UMU module of the pA58H3 plasmid cloned within KpnI site	This study
**Primer name**	**Primer sequence[*]**	**Coordinates in appropriate plasmid**
fLAM8H1<br>rLAM8H1	5'- ACTGTCGACTCCCGCAGAAGCCCAAATC -3'<br>5'- TCCGTCGACCCGGTAATCGTCCTGTCCAAC -3'	pA8H1<br>9,455 – 11,230
fTER40H2<br>rTER40H2	5'- TGCGCGAGCTCTCTCGGCATCCACGTTGATCGG -3'<br>5'- GGCCGAGCTCCGTCCTGCTGTTCAGTGCCTTC -3'	pA40H2<br>6,103 – 8,787
fUMU58H3<br>rUMU58H3	5'- TACGGTACCCTCGTTCCGTTACTACTC -3'<br>5'- CCAGGTACCAAGATGGCTCTCAGGAAG -3'	pA58H3<br>92 – 4,201

[*] Sequences shown in the 5′ to 3′ orientation. Sequences of the restriction sites were underlined.

References

Kovach, M.E., Phillips, R.W., Elzer, P.H., Roop, R.M., 2nd, and Peterson, K.M. (1994). pBBR1MCS: a broad-host-range cloning vector. *BioTechniques* 16**,** 800-802.
