# Supplementary material for: Insight Into the Diversity and Possible Role of Plasmids in the Adaptation of Psychrotolerant and Metalotolerant Arthrobacter spp. to Extreme Antarctic Environments
Source: Front Microbiol. 2018 Dec 18;9:3144. doi: 10.3389/fmicb.2018.03144 (PMC6305408; doi:10.3389/fmicb.2018.03144)
Supplement: Supplementary file 2 [file Table_2.pdf]

Supplementary Material

Insight into the Diversity and Possible Role of Plasmids in the Adaptation of Psychrotolerant and Metalotolerant *Arthrobacter* spp. to Extreme Antarctic Environments

Krzysztof Romaniuk, Piotr Golec, Lukasz Dziewit*
*** Correspondence:** Dr. Lukasz Dziewit: ldziewit@biol.uw.edu.pl

TABLE S2. Summarization of the sequencing data for the ANT plasmids.

Plasmid name	GenBank accession number	Contigs	Number of reads	Covarage
pA2H1	MH067968	contig 1<br>contig 2	18,701<br>571	630.9<br>590.1
pA2H2	MH067969	contig 1<br>contig 2	10,464<br>11,867	72.8<br>85.2
pA8H1	MH067977	contig 1<br>contig 2<br>contig 3<br>contig 4<br>contig 5	417<br>743<br>332<br>291<br>174	11.3<br>11.1<br>12.2<br>11.6<br>13.1
pA19BH1	MH067967	contig 1	6,634	25.5
pA40H1	MH067970	contig 1	18,059	56.6
pA40H2	MH067971	contig 1	9,366	26.2
pA44BH1	MH067972	contig 1	3,375	21.5
pA48BH1	MH067973	contig 1<br>contig 2	809<br>532	8.1<br>9.2
pA58H1	MH067974	contig 1	22,900	162.7
pA58H2	MH067975	contig 1	7,627	24.3
pA58H3	MH067976	contig 1	5,677	15.7

[a] Sequences shown in the 5′ to 3′ orientation.
