# Supplementary material for: Insight Into the Diversity and Possible Role of Plasmids in the Adaptation of Psychrotolerant and Metalotolerant Arthrobacter spp. to Extreme Antarctic Environments
Source: Front Microbiol. 2018 Dec 18;9:3144. doi: 10.3389/fmicb.2018.03144 (PMC6305408; doi:10.3389/fmicb.2018.03144)
Supplement: Supplementary file 3 [file Table_3.pdf]

Supplementary Material

Insight into the Diversity and Possible Role of Plasmids in the Adaptation of Psychrotolerant and Metalotolerant *Arthrobacter* spp. to Extreme Antarctic Environments

Krzysztof Romaniuk, Piotr Golec, Lukasz Dziewit*

*** Correspondence:** Dr. Lukasz Dziewit: ldziewit@biol.uw.edu.pl

TABLE S3. Genes located within the *Arthrobacter* spp. plasmids described in this study.

Gene name	Coding region (bp)	Orientation	Protein size (aa)	Possible function	Best BLAST hits		
					Percentage identity (aa)	Organism	GenBank accession no.
				**pA2H1**			
pA2H1_p...							
01	263-1171	→	302	replication protein	277/306 (91%)	*Glutamicibacter* sp. BW77	WP_096254262
02	1161-1412	→	83	replicase / regulator	79/83 (95%)	*Actinobacteria*	WP_078617992
03	1703-2101	→	132	Ars family regulator	132/132 (100%)	*Actinobacteria*	WP_048896403
04	2098-2976	→	292	membrane permease	288/292 (99%)	*Kocuria salsicia*	WP_055083813
05	3260-4246	→	328	transposase of IS481 family	328/328 (100%)	*Mycobacterium conceptionense*	WP_065062263
06	4708-5472	→	254	preprotein translocase	132/201 (66%)	*Microbacterium aurum*	WP_076690377
07	5669-6577	→	302	hypothetical protein	196/303 (65%)	*Actinotalea ferrariae*	WP_034220959
08	7574-6828	←	248	relaxase	194/250 (78%)	*Glutamicibacter halophytocola*	WP_060703571
09	8044-7571	←	157	mobilization protein C	127/161 (79%)	*Glutamicibacter halophytocola*	WP_060703570
				**pA2H2**			
pA2H2_p...							
01	414-1721	→	435	partitioning protein ParB	220/440 (50%)	*Arthrobacter sp. SRS-W-1-2016*	WP_078106487
02	2376-2696	→	106	hypothetical protein	27/86 (31%)	*Arthrobacter nitrophenolicus*	WP_009359328
03	3726-4715	→	329	hypothetical protein	208/329 (63%)	*Paeniglutamicibacter antarcticus*	WP_084382566
04	4809-5219	→	136	hypothetical protein	99/123 (80%)	*Paeniglutamicibacter antarcticus*	WP_068738522

05	5226-5630	→	134	hypothetical protein	99/115 (86%)	*Paeniglutamicibacter antarcticus*	WP_068738520
06	5633-5929	→	98	hypothetical protein	53/100 (53%)	*Paeniglutamicibacter antarcticus*	WP_068738518
07	6229-5933	←	98	hypothetical protein	31/76 (41%)	*Sinomonas mesophila*	WP_077490866
08	6594-6848	→	84	glutaredoxin NrdH	76/84 (90%)	*Paeniglutamicibacter antarcticus*	WP_068738516
09	7238-7642	→	134	hypothetical protein	78/132 (59%)	*Glutamicibacter arilaitensis Re117*	CBT77183
10	9000-8161	←	279	transposase OrfB, IS3 family, IS150 group	226/279 (81%)	*Arthrobacter alpinus*	WP_062008556
11	9503-9012	←	163	transposase OrfA, IS3 family, IS150 group	112/163 (69%)	*Arthrobacter alpinus*	WP_062005577
12	10716-9748	←	322	SulP family inorganic anion transporter, partial	294/318 (92%)	*Arthrobacter sp. UCD-GKA*	WP_071214384
13	10970-11878	→	302	transposase TniB, Tn3 family	222/301 (74%)	*Vitreoscilla filiformis*	WP_089416951
14	11875-12987	→	370	transposase TniQ, Tn3 family	147/335 (44%)	*Delftia tsuruhatensis*	WP_061287697
15	13417-14055	→	212	transposon ivertase/resolvase TniR	185/203 (91%)	*Arthrobacter sp. U41*	WP_069952892
16	14303-14983	→	226	ABC lipoprotein transporter, ATP-binding	173/226 (77%)	*Microbacterium sp. CH12i*	KDA04992
17	14980-16278	→	432	ABC-type permease	306/433 (71%)	*Microbacterium sp. CH12i*	WP_036280764
18	16290-16928	→	212	transcriptional regulator, TetR family	113/212 (53%)	*Leucobacter celer*	WP_083451407
19	17470-17120	←	116	transcriptional regulator, MarR family, partial	50/98 (51%)	*Arthrobacter nitrophenolicus*	WP_009356916
20	20265-17731	←	844	RND transporter, membrane protein YdfJ	774/822 (94%)	*Arthrobacter subterraneus*	WP_090588210
21	20925-20296	←	209	transcriptional regulator, TetR family	193/208 (93%)	*Pseudarthrobacter phenanthrenivorans*	WP_013602978
22	22611-20986	←	541	drug resistance transporter	472/541 (87%)	*Arthrobacter sp. Leaf69*	WP_056426022
23	23234-22608	←	208	transcriptional regulator, TetR family	136/207 (66%)	*Arthrobacter sp. I3*	WP_052355037
24	24119-24730	→	203	invertase/recombinase of TE	186/194 (96%)	*Arthrobacter sp. YC-RL1*	WP_047118360
25	24737-26197	→	486	transposase of ISNCY family	377/482 (78%)	*Brevibacterium yomogidense*	WP_087005899
26	26172-27077	→	301	ATP-binding protein of TE	238/301 (79%)	*Brevibacterium yomogidense*	WP_087005896
27	27229-27825	→	198	invertase	122/184 (66%)	*Arthrobacter sp. H20*	WP_026551421
28	27860-28153	→	97	hypothetical protein	33/93 (35%)	*Arthrobacter sp. KI72*	WP_079941698
29	28166-28642	→	158	hypothetical protein	93/157 (59%)	*Arthrobacter sp. Leaf337*	WP_055805228
30	29004-28639	←	121	hypothetical protein	60/120 (50%)	*Arthrobacter sp. Rue61a*	WP_014923458
31	29406-33911	→	1501	conjugal transfer protein TraA	911/1466 (62%)	*Arthrobacter sp. Rue61a*	WP_014923459
32	34017-34769	→	250	hypothetical protein	115/215 (53%)	*Mycobacterium abscessus*	WP_032691426
33	35843-34860	←	327	hypothetical protein	232/326 (71%)	*Paeniglutamicibacter antarcticus*	WP_068731261
34	36866-36363	←	167	hypothetical protein	148/172 (86%)	*Arthrobacter sp. W1*	KWR70626
35	38002-36866	←	378	peptidase M23	256/340 (75%)	*Paeniglutamicibacter antarcticus*	WP_068735610
36	38717-37995	←	240	hypothetical protein	97/167 (58%)	*Arthrobacter sp. KI72*	WP_079941686
37	40582-38714	←	622	conjugal transfer protein TraG	374/592 (63%)	*Arthrobacter sp. ZXY-2*	WP_071416994
38	42046-40604	←	480	conjugal transfer ATPase	331/489 (68%)	*Arthrobacter sp. Hiyo1*	WP_059390189
39	43788-42163	←	541	hypothetical protein	343/541 (63%)	*Arthrobacter sp. Hiyo1*	WP_059390188
40	45535-43979	←	518	hypothetical protein	262/504 (52%)	*Arthrobacter sp. KI72*	WP_079941690
41	46247-45582	←	221	hypothetical protein	77/219 (35%)	*Arthrobacter sp. Hiyo1*	WP_059390186

42	46612-46250	←	120	hypothetical protein	65/120 (54%)	*Arthrobacter sp. ZXY-2*	AOY74439
43	47763-46609	←	384	hypothetical protein	213/379 (56%)	*Arthrobacter sp. Hiyo1*	WP_059390184
44	47958-48251	→	97	transcriptional regulator, ArsR family	69/96 (72%)	*Arthrobacter sp. AK-1*	WP_012311513
45	48373-49104	→	243	hypothetical protein	105/243 (43%)	*Arthrobacter saudimassiliensis*	CEA07078
46	49104-49832	→	242	hypothetical protein	112/240 (47%)	*Tersicoccus sp. Bi-70*	WP_076695439
47	49906-50136	→	76	hypothetical protein	47/71 (66%)	*Glutamicibacter sp. BW80*	WP_096289390
48	50289-51716	→	475	hypothetical protein	197/367 (54%)	*Glutamicibacter sp. BW77*	WP_096253930
49	51902-52672	→	256	partitioning protein ParA	182/256 (71%)	*Paeniglutamicibacter antarcticus*	WP_068738528
50	52669-52995	→	117	partitioning protein ParB	38/106 (36%)	*Arthrobacter subterraneus*	SDI70605
51	53459-53893	→	144	DNA polymerase V subunit UmuD	136/143 (95%)	*Paeniglutamicibacter gangotriensis*	WP_007273049
52	54048-55169	→	373	tyrosine recombinase XerC	372/373 (99%)	*Paeniglutamicibacter gangotriensis*	WP_007273086
53	55166-55516	→	116	transcriptional regulator, XRE family	116/116 (100%)	*Paeniglutamicibacter gangotriensis*	WP_007273085
54	55506-57992	→	828	site-specific recombinase XerD	827/828 (99%)	*Paeniglutamicibacter gangotriensis Lz1y*	EMQ96799
55	58251-59273	→	340	tellurium resistance membrane protein TerC	340/340 (100%)	*Paeniglutamicibacter gangotriensis*	WP_007273083
56	59270-60103	→	277	cation-transporting ATPase	277/277 (100%)	*Paeniglutamicibacter gangotriensis*	WP_007273082
57	60300-60605	→	101	transcription termination factor Rho	101/101 (100%)	*Paeniglutamicibacter gangotriensis*	WP_007273081
58	60712-61293	→	193	thymidylate kinase	193/193 (100%)	*Paeniglutamicibacter gangotriensis*	WP_007273080
59	61405-62739	→	444	DNA polymerase V subunit UmuC	401/436 (92%)	*Paeniglutamicibacter gangotriensis*	WP_007273048
60	63954-63601	←	117	hypothetical protein	-/-	*none*	-
				**pA8H1**			
pA8H1_p...							
1	78-728	→	216	partitioning protein A	177/214 (83%)	*Arthrobacter sp. YC-RL1*	WP_047117786
2	725-961	→	78	partitioning protein B	57/78 (78%)	*Arthrobacter sp. YC-RL1*	WP_047117785
3	1365-1799	→	144	DNA polymerase V subunit UmuD	136/143 (95%)	*Paeniglutamicibacter gangotriensis*	WP_007273049
4	1954-3075	→	373	tyrosine recombinase XerC	371/373 (99%)	*Paeniglutamicibacter gangotriensis*	WP_007273086
5	3072-3422	→	116	transcriptional regulator, XRE family	116/116 (100%)	*Paeniglutamicibacter gangotriensis*	WP_007273085
6	3412-5898	→	828	site-specific recombinase XerD	827/828 (99%)	*Paeniglutamicibacter gangotriensis Lz1y*	EMQ96799
7	6157-7079	→	340	tellurium resistance protein TerC	340/340 (100%)	*Paeniglutamicibacter gangotriensis*	WP_007273083
8	7176-8009	→	277	cation-transporting ATPase	277/277 (100%)	*Paeniglutamicibacter gangotriensis*	WP_007273082
9	8206-8511	→	101	putative termination factor Rho	101/101 (100%)	*Paeniglutamicibacter gangotriensis*	WP_007273081
10	8618-9199	→	193	thymidylate kinase	193/193 (100%)	*Paeniglutamicibacter gangotriensis*	WP_007273080
11	9311-9577	→	88	DNA polymerase V subunit UmuC, partial	81/88 (92%)	*Paeniglutamicibacter gangotriensis Lz1y*	EMQ96794
12	9690-10781	→	363	laminarinase	363/363 (100%)	*Paeniglutamicibacter gangotriensis*	WP_007273079
13	11340-11047	←	97	hypothetical protein	84/97 (87%)	*Paeniglutamicibacter gangotriensis*	WP_007273077
14	11768-11923	→	51	hypothetical protein	50/51 (98%)	*Paeniglutamicibacter gangotriensis Lz1y*	EMQ96791
15	13183-11993	←	396	filamentation protein	396/396 (100%)	*Paeniglutamicibacter gangotriensis*	WP_007273075
16	13722-14744	→	340	tellurium resistance protein TerC	340/340 (100%)	*Paeniglutamicibacter gangotriensis*	WP_007273074
17	14886-15461	→	191	hypothetical protein	191/191 (100%)	*Paeniglutamicibacter gangotriensis*	WP_007273072

18	16738-15548	←	396	hypothetical protein	167/393 (42%)	*Microbacterium ginsengisoli*	WP_045248425
19	17087-16740	←	115	transcriptional regulator, XRE family	87/117 (74%)	*Pseudonocardia autotrophica*	OSY36007
20	18211-17084	←	375	tyrosine recombinase XerC	262/360 (73%)	*Microbacterium ginsengisoli*	KJL35305
21	18429-19442	→	337	DNA polymerase V subunit UmuC	316/337 (94%)	*Arthrobacter sp. UCD-GKA*	WP_071216190
22	20561-19515	←	348	hypothetical protein	191/328 (58%)	*Glutamicibacter halophytocola*	WP_060700398
23	21731-22711	→	326	hypothetical protein	234/319 (73%)	*Arthrobacter sp. YC_RL1*	WP_052772146
24	23294-24283	→	329	hypothetical protein	207/329 (63%)	*Paeniglutamicibacter antarcticus*	WP_084382566
25	24365-24781	→	138	hypothetical protein	103/133 (77%)	*Paeniglutamicibacter antarcticus*	WP_068738522
26	24794-25198	→	134	hypothetical protein	113/134 (84%)	*Paeniglutamicibacter antarcticus*	WP_068738520
27	25201-25497	→	98	hypothetical protein	52/100 (52%)	*Paeniglutamicibacter antarcticus*	WP_068738518
28	26211-28061	→	616	type I RM protein subunit M	361/630 (57%)	*Paenibacillus sabinae*	WP_025334610
29	28064-28567	→	167	type I RM protein subunit S, truncated	83/158 (53%)	*Mycobacterium abscessus*	WP_079609079
30	28465-29034	→	189	type I RM protein subunit S, truncated	92/180 (51%)	*Actinobacteria*	WP_051132165
31	30027-29314	←	237	hypothetical protein	148/232 (64%)	*Kocuria sp. SM24M-10*	WP_047803197
32	30429-30031	←	132	hypothetical protein	85/115 (74%)	*Kocuria sp. SM24M-10*	KLU08433
33	32295-30772	←	507	sulfate permease	479/507 (94%)	*Arthrobacter sp. UCD-GKA*	WP_071214384
34	32482-32874	→	130	transcriptional regulator, MerR family	112/129 (87%)	*Paeniglutamicibacter gangotriensis*	WP_007272368
35	33583-33326	←	85	transcriptional regulator, CsoR family	85/85 (100%)	*Arthrobacter*	WP_022876876
36	33750-33926	→	58	hypothetical protein	52/58 (90%)	*Arthrobacter sp. 7749*	ASN39331
37	34101-35498	→	465	persulfide dioxygenase	465/465 (100%)	*Arthrobacter*	WP_022876878
38	35613-36197	→	194	sulfurtransferase	193/194 (99%)	*Arthrobacter*	WP_022876879
39	36318-36614	→	98	sulfurtransferase	98/98 (100%)	*Arthrobacter sp. YC_RL1*	WP_047117791
40	36680-37576	→	298	sulfite exporter TauE/SafE family	291/298 (98%)	*Arthrobacter sp. PAO19*	WP_022876881
41	37631-38959	→	442	MFS transporter	440/442 (99)%	*Arthrobacter sp. YC_RL1*	WP_047117789
42	39048-39467	→	139	ABC transporter	139/139 (100%)	*Arthrobacter*	WP_022876883
43	39845-40456	→	203	resolvase	202/203 (99%)	*Paeniglutamicibacter gangotriensis Lz1y*	EMQ96627
44	40687-41415	→	242	hypothetical protein	113/240 (47%)	*Tersicoccus sp. Bi-70*	WP_076695439
45	41493-41723	→	76	hypothetical protein	41/71 (58%)	*Arthrobacter sp. YC_RL1*	WP_082123748
46	41876-43279	→	467	hypothetical protein	209/439 (48%)	*Arthrobacter sp. YC_RL1*	WP_047117787
				**pA40H1**			
pA40H1_p...							
01	90-860	→	256	partitioning protein ParA	180/256 (70%)	*Paeniglutamicibacter antarcticus*	WP_068738528
02	857-1138	→	93	partitioning protein ParB	32/96 (33%)	*Microbacterium sp. CH12i*	WP_036282148
03	1534-1181	←	117	truncated transposase	33/43 (77%)	*Paeniglutamicibacter gangotriensis Lz1y*	EMQ96780
04	2414-1875	←	179	DNA invertase/recombinase	168/179 (94%)	*Arthrobacter alpinus*	WP_074713669
05	2841-2431	←	136	hypothetical protein, truncated transposase	64/98 (65%)	*Arthrobacter alpinus*	WP_062008744
06	2925-3134	→	69	DNA polymerase V subunit UmuC, partial	56/63 (89%)	*Paeniglutamicibacter gangotriensis*	WP_007273048
07	3446-3252	←	64	transcriptional regulator, XRE-family	54/63 (86%)	*Arthrobacter sp. Edens01*	WP_055240980

08	3910-3446	←	154	hypothetical protein	84/150 (56%)	*Arthrobacter koreensis*	WP_091604535
09	4193-4777	→	194	resolvase, DNA invertase	194/194 (100%)	*Arthrobacter sp. TB 23*	WP_019481146
10	5029-6003	→	324	hypothetical protein	207/322 (64%)	*Arthrobacter sp. J3.40*	WP_015061943
11	6903-6247	←	218	hypothetical protein	-/-	*none*	-
12	7660-8274	→	204	hypothetical protein	169/198 (85%)	*Paeniglutamicibacter gangotriensis*	WP_007273036
13	8581-10098	→	505	type I restriction-modification system, subunit M, SAM-dependent	409/505 (81%)	*Arthrobacter crystallopoietes*	WP_005266257
14	10292-11305	→	337	type I restriction-modification system, subunit S	187/408 (46%)	*Arthrobacter crystallopoietes*	WP_081608413
15	11306-11785	→	159	addiction module toxin	76/154 (49%)	*Rhodococcus pyridinivorans*	WP_064060139
16	11875-12216	→	113	addiction module antitoxin	73/113 (65%)	*Arthrobacter sp. W1*	WP_052253545
17	12330-15359	→	1009	type I restriction endonuclease subunit R	747/1012 (74%)	*Arthrobacter crystallopoietes*	WP_005269194
18	18864-15407	←	1152	HNH endonuclease	720/1141 (63%)	*Leucobacter sp. UCD-THU*	WP_031289558
19	19136-19309	→	57	invertase, partial	32/40 (80%)	*Paeniglutamicibacter gangotriensis*	WP_007272296
20	20058-19375	←	227	hypothetical protein	133/221 (60%)	*Mycobacterium sinense*	WP_064922354
21	21403-20252	←	383	Zn-dependent peptidase ImmA, M78 family	213/381 (56%)	*Paenarthrobacter nicotinovorans*	GAT87930
22	22046-21396	←	216	hypothetical protein	113/217 (52%)	*Paenarthrobacter nicotinovorans*	WP_064722765
23	23604-22498	←	368	hypothetical protein	180/373 (48%)	*Micromonospora narathiwatensis*	WP_091191950
24	24322-23750	←	190	DNA resolvase	184/190 (97%)	*Arthrobacter sp. UCD-GKA*	WP_071216176
25	25223-24921	←	100	hypothetical protein	57/99 (58%)	*Paeniglutamicibacter antarcticus*	WP_068738518
26	25917-25585	←	110	hypothetical protein	59/110 (54%)	*Arthrobacter sp. B6*	WP_066286544
27	27503-26505	←	332	pepsidase	190/298 (64%)	*Arthrobacter sp. UCD-GKA*	WP_071216184
28	28161-27619	←	180	hypothetical protein	88/180 (49%)	*Arthrobacter sp. ok362*	WP_091561456
29	30386-28806	←	526	signal transduction histidine kinase	406/525 (77%)	*Arthrobacter sp. UCD-GKA*	WP_071216182
30	31237-30386	←	283	alpha/beta hydrolase	229/283 (81%)	*Arthrobacter sp. UCD-GKA*	WP_071216183
31	31536-31790	→	84	hypothetical protein	44/80 (55%)	*Arthrobacter sp. ZXY-2*	WP_071416815
32	33179-32472	←	235	hypothetical protein	194/235 (83%)	*Paeniglutamicibacter antarcticus*	WP_068731980
33	34106-34816	→	236	resolvase/DNA invertase	157/207 (76%)	*Arthrobacter sp. H5*	WP_081746535
34	34767-40304	→	1845	adenine-specific DNA methylase, N12 class, helicase	1256/1847 (68%)	*Arthrobacter luteolus*	WP_083521870
35	40810-40526	←	94	hypothetical protein	55/89 (62%)	*Arthrobacter crystallopoietes*	WP_005272797
36	41003-41398	→	131	mobilization protein C	86/128 (67%)	*Glutamicibacter arilaitensis Re117*	CBT74902
37	41395-43056	→	553	relaxase	459/554 (83%)	*Paeniglutamicibacter antarcticus*	WP_068731965
38	43101-44555	→	484	hypothetical protein	316/396 (80%)	*Paeniglutamicibacter antarcticus*	WP_068731963
39	44669-46462	→	601	hypothetical protein	344/596 (58%)	*Nocardioides sp. Iso805N*	WP_081615574
40	46945-46532	←	137	hypothetical protein	90/115 (78%)	*Paeniglutamicibacter antarcticus*	WP_084382367
41	49001-47202	←	599	conjugal transfer protein TraG	556/599 (93%)	*Paeniglutamicibacter antarcticus*	WP_068735628
42	50528-49002	←	502	ATP-binding protein	483/496 (97%)	*Paeniglutamicibacter antarcticus*	WP_068735626
43	52036-50528	←	502	hypothetical protein	488/502 (97%)	*Paeniglutamicibacter antarcticus*	WP_068735625

44	53466-52033	←	477	hypothetical protein	151/256 (59%)	*Arthrobacter sp. J3.40*	WP_015061895
45	54185-53463	←	240	hypothetical protein	147/166 (89%)	*Paeniglutamicibacter antarcticus*	WP_068735621
46	54489-54205	←	94	hypothetical protein	91/94 (97%)	*Paeniglutamicibacter antarcticus*	WP_068735618
47	55076-54486	←	196	hypothetical protein	148/195 (76%)	*Paeniglutamicibacter antarcticus*	WP_068735616
48	56521-55094	←	475	partitioning protein A	406/482 (84%)	*Paeniglutamicibacter antarcticus*	WP_068735614
49	57030-56554	←	158	hypothetical protein	126/158 (80%)	*Paeniglutamicibacter antarcticus*	WP_068735612
50	58187-57075	←	370	peptidase, M23 family	315/350 (90%)	*Paeniglutamicibacter antarcticus*	WP_068735610
51	58343-58654	→	103	DNA-binding protein	73/78 (94%)	*Paeniglutamicibacter antarcticus*	WP_068735608
52	59267-58674	←	197	transposase, Tn3 family	205/207 (99%)	*Glutamicibacter sp. 0426*	WP_073708019
53	61641-59272	←	789	transposase, Tn3 family	772/781 (99%)	*Glutamicibacter sp. 0426*	WP_073708019
54	62292-61720	←	190	resolvase	189/190 (99%)	*Glutamicibacter sp. BW80*	WP_096287908
55	62613-62819	→	68	copper chaperone, CopZ	68/68 (100%)	*Micrococcaceae*	WP_047117896
56	62816-63751	→	311	heavy metal-binding protein	311/311 (100%)	*Micrococcaceae*	WP_047117895
57	63800-66067	→	755	copper-translocating P-type ATPase, CopA	730/755 (97%)	*Glutamicibacter sp. 0426*	WP_073708020
58	66304-66191	←	37	hypothetical protein	36/37 (97%)	*Acinetobacter gyllenbergii*	EPH35188
59	66593-66279	←	104	transcriptional regulator, ArsR family	104/104 (100%)	*Proteobacteria*	WP_000349485
60	66705-67739	→	344	permease	343/344 (99%)	*Proteobacteria*	WP_000098298
61	67752-69029	→	425	transposase, ISL3 family	425/425 (100%)	*Proteobacteria*	WP_000610830
62	69112-69381	→	89	hypothetical protein	83/89 (93%)	*Micrococcaceae*	WP_047117893
63	69667-69996	→	109	transcriptional regulator, CopY family	109/109 (100%)	*Micrococcaceae*	WP_047117892
64	70303-70936	→	211	hypothetical protein	211/211 (100%)	*Micrococcaceae*	WP_047117907
65	71204-71785	→	193	alkylhydroperoxidase	192/193 (99%)	*Arthrobacter sp. YC-RL1*	KLI90490
66	71865-73298	→	477	MFS transporter, H^+ antiporter protein	474/477 (99%)	*Micrococcaceae*	WP_047117890
67	73814-74380	→	188	hypothetical protein	146/184 (79%)	*Paeniglutamicibacter antarcticus*	WP_068735606
68	75081-74353	←	242	hypothetical protein	192/242 (79%)	*Paeniglutamicibacter antarcticus*	WP_068735604
69	75773-75081	←	230	hypothetical protein	199/230 (87%)	*Paeniglutamicibacter antarcticus*	WP_068735602
70	76046-76273	→	75	hypothetical protein	45/75 (60%)	*Arthrobacter sp. SRS-W-1-2016*	WP_078106431
71	76563-78287	→	574	transcriptional regulator, MarR family	413/576 (72%)	*Paeniglutamicibacter antarcticus*	WP_068735600
				**pA40H2**			
pA40H2_p...							
01	241-1038	→	265	partitioning protein ParA	191/265 (72%)	*Kocuria marina*	KHE74185
02	1035-1397	→	120	hypothetical protein	76/120 (63%)	*Kocuria marina*	WP_035964271
03	1790-2224	→	144	DNA polymerase V subunit UmuD	133/143 (93%)	*Paeniglutamicibacter gangotriensis*	WP_007273049
04	2379-3500	→	373	tyrosine recombinase XerD	372/373 (99%)	*Paeniglutamicibacter gangotriensis*	WP_007273086
05	3497-3847	→	116	transcriptional regulator, XRE family	116/116 (100%)	*Paeniglutamicibacter gangotriensis*	WP_007273085
06	3837-6323	→	828	Site-specific recombinase XerC	826/828 (99%)	*Paeniglutamicibacter gangotriensis Lz1y*	EMQ96799
07	6582-7604	→	340	tellurium resistance protein TerC	340/340 (100%)	*Paeniglutamicibacter gangotriensis*	WP_007273083
08	7601-8434	→	277	cation-transporting ATPase	277/277 (100%)	*Paeniglutamicibacter gangotriensis*	WP_007273082

09	8631-8936	→	101	transcription termination factor Rho	101/101 (100%)	*Paeniglutamicibacter gangotriensis*	WP_007273081
10	9043-9624	→	193	thymidylate kinase	193/193 (100%)	*Paeniglutamicibacter gangotriensis*	WP_007273080
11	9736-11070	→	444	DNA polymerase V subunit UmuC	400/432 (93%)	*Paeniglutamicibacter gangotriensis*	WP_007273048
12	11539-11715	→	58	hypothetical protein	41/58 (71%)	*Arthrobacter sp. YC-RL1*	WP_047117640
13	11750-12730	→	326	hypothetical protein	230/319 (72%)	*Arthrobacter sp. YC-RL1*	WP_052772146
14	14888-15877	→	105	hypothetical protein	27/86 (31%)	*Arthrobacter nitrophenolicus*	WP_009359328
15	15971-16375	→	?	hypothetical protein	230/361 (64%)	*Paeniglutamicibacter antarcticus*	WP_084382566
16	15959-16375	→	138	hypothetical protein	98/123 (80%)	*Paeniglutamicibacter antarcticus*	WP_068738522
17	16388-16792	→	134	hypothetical protein	116/133 (87%)	*Paeniglutamicibacter antarcticus*	WP_068738520
18	16795-17091	→	98	hypothetical protein	53/100 (53%)	*Paeniglutamicibacter antarcticus*	WP_068738518
19	17095-17376	←	93	hypothetical protein	31/76 (41%)	*Sinomonas mesophila*	WP_077490866
20	17755-18009	→	84	NrdH-redoxin	69/84 (82%)	*Paeniglutamicibacter antarcticus*	WP_068738516
21	18006-18206	→	66	hypothetical protein	45/52 (87%)	*Paeniglutamicibacter antarcticus*	WP_068738514
22	18203-18637	→	144	hypothetical protein	32/90 (36%)	*Photobacterium piscicola*	WP_080157527
23	18838-19242	→	134	hypothetical protein	82/132 (62%)	*Glutamicibacter sp. BW77*	WP_096254231
24	22664-19320	←	1114	DNA methyltransferase	628/937 (67%)	*Arthrobacter sp. RIT-PI-e*	WP_082177329
25	24182-22719	←	487	hypothetical protein	427/487 (88%)	*Arthrobacter sp. 49Tsu3.1M3*	SKB43645
26	24784-24179	←	201	hypothetical protein	179/201 (89%)	*Arthrobacter sp. 49Tsu3.1M3*	SKB43651
27	26832-25309	←	507	SulP family inorganic anion transporter	480/507 (95%)	*Arthrobacter sp. UCD-GKA*	WP_071214384
28	27019-27411	→	130	transcriptional regulator, MerR family	112/129 (87%)	*Paeniglutamicibacter gangotriensis*	WP_007272368
29	28111-27854	←	85	transcriptional regulator, CsoR family	85/85 (100%)	*Micrococcaceae*	WP_022876876
30	28278-28454	→	58	hypothetical protein	53/58 (91%)	*Arthrobacter sp. 7749*	ASN39331
31	28629-30026	→	465	persulfide dioxygenase A	459/465 (99%)	*Arthrobacter*	WP_022876878
32	30114-30725	→	203	rhodanese-related sulfurtransferase	190/203 (94%)	*Glutamicibacter sp. BW80*	WP_096287256
33	30816-31112	→	98	rhodanese-related sulfurtransferase	98/98 (100%)	*Arthrobacter sp. UCD-GKA*	WP_071214388
34	31176-32072	→	298	sulfite exporter TauE/SafE	297/298 (99%)	*Glutamicibacter sp. BW80*	WP_096287260
35	32127-33455	→	442	MFS transporter	435/442 (98%)	*Paeniglutamicibacter gangotriensis*	WP_007273058
36	33542-33961	→	139	hypothetical protein	138/139 (99%)	*Paeniglutamicibacter gangotriensis*	WP_007273057
37	34380-34030	←	116	intracellular sulfur oxidation protein	116/116 (100%)	*Paeniglutamicibacter gangotriensis*	WP_007273056
38	35285-34377	←	302	cytochrome C biogenesis protein CcdA	301/302 (99%)	*Paeniglutamicibacter gangotriensis*	WP_007273055
39	36088-35285	←	267	thiol-disulfide oxidoreductase	266/267 (99%)	*Paeniglutamicibacter gangotriensis*	WP_007273054
40	36223-36774	→	183	cytochrome c-type biogenesis protein CcdA (DsbD analog)	183/183 (100%)	*Paeniglutamicibacter gangotriensis*	WP_007273053
41	36771-37169	→	132	transcriptional regulator	131/132 (99%)	*Paeniglutamicibacter gangotriensis*	WP_007273052
42	37518-38129	→	203	resolvase	202/203 (99%)	*Paeniglutamicibacter gangotriensis Lz1y*	EMQ96627
43	39342-38410	←	310	hypothetical protein	102/293 (35%)	*Brevibacterium linens*	WP_096160932
44	39687-41099	→	470	hypothetical protein	193/456 (42%)	*Leifsonia aquatica*	WP_025155972
45	41683-42285	→	200	hypothetical protein	178/202 (88%)	*Paeniglutamicibacter gangotriensis*	WP_007273036
46	43931-43092	←	279	endonuclease	239/278 (86%)	*Arthrobacter sp. AK-YN10*	ERI35594

47	45084-43921	←	387	DNA cytosine methyltransferase	235/381 (62%)	*Rhodococcus*	WP_094633520
48	45518-46129	→	203	serine resolvase, DNA invertase	112/201 (56%)	*Arthrobacter sp. P2b*	WP_079599224
49	46822-46205	←	205	hypothetical protein	45/197 (23%)	*Cryobacterium luteum*	SEO03821
50	47232-46909	←	107	DNA-cytosine methyltransferase, partial	38/69 (55%)	*Rhodococcus sp. AW25M09*	CCQ14341
51	49276-47501	←	591	abortive infection phage resistance protein AbiU	269/568 (47%)	*Streptomyces achromogenes*	WP_078844454
52	49665-50078	→	137	hypothetical protein	57/145 (39%)	*Leucobacter komagatae*	WP_042543649
53	50159-50515	→	118	hypothetical protein	66/106 (62%)	*Nesterenkonia massiliensis*	WP_044495328
54	50809-52026	→	405	hypothetical protein	125/409 (31%)	*Paenarthrobacter nicotinovorans*	WP_064721708
55	52635-52027	←	202	DNA resolvase	182/190 (96%)	*Arthrobacter sp. UCD-GKA*	WP_071215874
56	53001-53624	→	207	hypothetical protein	-/-	*none*	-
57	53617-54354	→	245	hypothetical protein	-/-	*none*	-
58	54609-55304	→	231	hypothetical protein	152/231 (66%)	*Paeniglutamicibacter gangotriensis*	WP_007273096
59	55600-56310	→	236	DNA resolvase/invertase	146/193 (76%)	*Arthrobacter sp. H5*	WP_081746535
60	56261-61795	→	1844	adenine-specific DNA methylase, N12 class	1246/1832 (68%)	*Arthrobacter luteolus*	WP_083521870
61	62321-62037	←	94	hypothetical protein	59/90 (66%)	*Arthrobacter sp. EPSL27*	WP_066435256
62	62515-62907	→	130	mobilization protein MobC	77/126 (61%)	*Arthrobacter crystallopoietes*	SDR29386
63	62904-64577	→	557	relaxase	410/558 (73%)	*Paeniglutamicibacter gangotriensis*	WP_068731965
64	64622-65824	→	400	hypothetical protein	326/400 (82%)	*Paeniglutamicibacter gangotriensis*	WP_068731963
65	66239-66730	→	163	hypothetical protein	58/140 (41%)	*Microbacterium chocolatum*	WP_053549043
66	67949-67518	←	143	hypothetical protein	81/115 (70%)	*Paeniglutamicibacter gangotriensis*	WP_084382367
67	69972-68173	←	599	conjugal transfer protein TraG	574/599 (96%)	*Paeniglutamicibacter antarcticus*	WP_068735628
68	71451-69973	←	492	ATP/GTP-binding conjugal protein	478/497 (96%)	*Paeniglutamicibacter antarcticus*	WP_068735626
69	72956-71448	←	502	hypothetical membrane protein	489/502 (99%)	*Paeniglutamicibacter antarcticus*	WP_068735625
70	74548-72953	←	531	hypothetical protein	247/264 (94%)	*Paeniglutamicibacter antarcticus*	WP_068735622
71	75267-74545	←	240	hypothetical protein	136/166 (82%)	*Paeniglutamicibacter antarcticus*	WP_068735621
72	75571-75287	←	94	hypothetical protein	91/94 (97%)	*Paeniglutamicibacter antarcticus*	WP_068735618
73	76158-75568	←	196	hypothetical protein	147/196 (75%)	*Paeniglutamicibacter antarcticus*	WP_068735616
74	77600-76176	←	474	partitioning protein ParA	409/478 (86%)	*Paeniglutamicibacter antarcticus*	WP_068735614
75	78143-77631	←	170	hypothetical protein	158/170 (93%)	*Paeniglutamicibacter antarcticus*	WP_068735612
76	79276-78140	←	378	peptidase M23	314/349 (90%)	*Paeniglutamicibacter antarcticus*	WP_068735610
77	79427-79699	→	90	transcriptional regulator, ArsR family	56/90 (62%)	*Arthrobacter castelli*	WP_035770425
78	79851-80582	→	243	transcriptional regulator, XRE family	102/243 (42%)	*Arthrobacter saudimassiliensis*	CEA07078
79	80582-81310	→	242	hypothetical protein	116/242 (48%)	*Tersicoccus sp. Bi-70*	WP_076695439
80	81384-81611	→	75	hypothetical protein	50/75 (67%)	*Arthrobacter sp. AK-YN10*	WP_043427946
81	81825-82862	→	345	hypothetical protein	182/345 (53%)	*Arthrobacter sp. TB 26*	WP_024366458
82	82943-86035	→	1030	Tn3 family transposase	1029/1030 (99%)	*Paeniglutamicibacter gangotriensis*	WP_007272224
83	86213-86356	→	47	hypothetical protein, partial	30/38 (79%)	*Arthrobacter sp. YC-RL1*	KLI90155
84	86706-86440	←	88	hypothetical protein, cytochrome terminal	87/88 (99%)	*Paeniglutamicibacter gangotriensis Lz1y*	EMQ97673

85	88051-86768	←	427	hypothetical protein	427/427 (100%)	*Paeniglutamicibacter gangotriensis Lz1y*	EMQ97674
86	88793-88596	←	65	hypothetical protein	65/65 (100%)	*Paeniglutamicibacter gangotriensis*	WP_083874168
87	89048-88914	←	44	hypothetical protein	44/44 (100%)	*Paeniglutamicibacter gangotriensis Lz1y*	EMQ97676
88	89316-89897	→	193	serine resolvase	192/193 (99%)	*Paeniglutamicibacter gangotriensis*	WP_007272219
89	90247-90618	→	123	hypothetical protein	47/126 (37%)	*Arthrobacter sp. PAO19*	WP_022875896
				**pA58H1**			
pA58H1_p...							
01	172-933	→	253	partitioning protein ParA	182/249 (73%)	*Microbacterium esteraromaticum*	SJN25287
02	1011-1622	→	203	hypothetical protein	109/203 (54%)	*Microbacterium sp. H83*	WP_067117699
03	1715-2011	→	98	hypothetical protein	42/86 (49%)	*Arthrobacter sp. 35W*	WP_026554317
04	2008-2319	→	103	hypothetical protein	-/-	*none*	-
05	2442-2669	→	75	hypothetical protein	51/72 (71%)	*Arthrobacter sp. YC-RL1*	WP_060617064
06	3339-3539	→	66	hypothetical protein	44/64 (69%)	*Arthrobacter sp. YC-RL1*	WP_060617064
07	4566-3622	←	314	type I restriction endonuclease subunit S	218/313 (70%)	*Clavibacter michiganensis*	WP_053774982
08	6419-4563	←	618	type I restriction endonuclease subunit M	491/610 (80%)	*Gordonia sp. 852002-10350_SCH5691597*	WP_064861386
09	6855-7532	→	225	DNA invertase/resolvase	164/207 (79%)	*Arthrobacter sp. H20*	WP_081743482
10	7598-8002	→	134	hypothetical protein	71/130 (55%)	*Arthrobacter sp. H5*	WP_035775518
11	8041-13062	→	1673	Adenine-specific DNA methylase, N12 class, helicase	1155/1661 (70%)	*Arthrobacter luteolus*	WP_083521870
12	14041-13766	←	91	hypothetical protein	55/88 (63%)	*Arthrobacter sp. U41*	WP_069953057
13	14230-14628	→	132	mobilization protein MobC	88/103 (85%)	*Paeniglutamicibacter antarcticus*	WP_068731966
14	14625-16289	→	554	relaxase	448/554 (81%)	*Paeniglutamicibacter antarcticus*	WP_068731965
15	16327-17526	→	399	hypothetical protein	290/400 (73%)	*Paeniglutamicibacter antarcticus*	WP_068731963
16	18058-17603	←	152	hypothetical protein	93/109 (85%)	*Paeniglutamicibacter antarcticus*	WP_084382367
17	20102-18303	←	599	conjugal transfer protein	566/599 (94%)	*Paeniglutamicibacter antarcticus*	WP_068735628
18	21587-20103	←	494	ATP-binding protein	482/496 (97%)	*Paeniglutamicibacter antarcticus*	WP_068735626
19	23122-21614	←	502	hypothetical protein	489/502 (97%)	*Paeniglutamicibacter antarcticus*	WP_068735625
20	24702-23119	←	527	hypothetical protein	247/264 (94%)	*Paeniglutamicibacter antarcticus*	WP_068735622
21	25415-24699	←	238	hypothetical protein	138/167 (83%)	*Paeniglutamicibacter antarcticus*	WP_068735621
22	25727-25443	←	94	hypothetical protein	91/94 (97%)	*Paeniglutamicibacter antarcticus*	WP_068735618
23	26314-25724	←	196	hypothetical protein	145/195 (74%)	*Paeniglutamicibacter antarcticus*	WP_068735616
24	27756-26332	←	474	partitioning protein ParA	408/478 (85%)	*Paeniglutamicibacter antarcticus*	WP_068735614
25	28299-27787	←	170	hypothetical protein	158/170 (93%)	*Paeniglutamicibacter antarcticus*	WP_068735612
26	29042-28296	←	248	peptidase M23	236/248 (95%)	*Paeniglutamicibacter antarcticus*	WP_068735610
27	29611-29883	→	90	transcriptional regulator, ArsR family	47/87 (54%)	*Arthrobacter castelli*	WP_035770425
28	30047-30274	→	75	hypothetical protein	38/72 (53%)	*Glutamicibacter sp. BW77*	WP_096253929
29	30424-31842	→	472	hypothetical protein	188/367 (51%)	*Glutamicibacter sp. BW77*	WP_096253930
30	32235-32585	→	116	hypothetical protein	50/112 (45%)	*Corynebacterium crudilactis*	WP_066570273

31	33297-33641	→	114	hypothetical protein	65/114 (57%)	*Microbacterium esteraromaticum*	WP_087130336
				**pA58H2**			
pA58H2_p...							
01	276-1046	→	256	partitioning protein ParA	250/256 (98%)	*Paeniglutamicibacter antarcticus*	WP_068738528
02	1043-1327	→	94	partitioning protein ParB	46/117 (39%)	*Kocuria sp. UCD-OTCP*	WP_017832696
03	2218-1607	←	203	hypothetical protein	184/203 (91%)	*Arthrobacter sp. UCD-GKA*	WP_071216150
04	2465-2674	→	69	hypothetical protein	58/69 (84%)	*Paeniglutamicibacter gangotriensis*	WP_007273045
05	2840-3886	→	348	hypothetical protein	297/342 (87%)	*Arthrobacter sp. ok909*	WP_091256393
06	7200-4195	←	1001	endonuclease of type III RM system	467/1002 (47%)	*Actinotignum timonense*	WP_087070271
07	8881-7205	←	558	DNA-methyltransferase, truncated	267/559 (48%)	*Anaerococcus rubeinfantis*	WP_058991267
08	10417-8978	←	479	transposase of 481-family	452/479 (94%)	*Arthrobacter sp. UCD-GKA*	WP_071216516
09	10824-10468	←	118	DNA-methyltransferase, truncated	52/99 (53%)	*Corynebacterium sp. L2-79-05*	WP_011867707
10	11646-11011	←	211	hypothetical protein	136/210 (65%)	*Glutamicibacter sp. 0426*	WP_073706563
11	12266-11730	←	178	hypothetical protein	91/167 (54%)	*Sinomonas mesophila*	WP_077490879
12	12867-12553	←	104	transcriptional regulator, ArsR family	104/104 (100%)	*Proteobacteria*	WP_000349485
13	13621-14013	→	130	permease, partial	129/130 (99%)	*Oleibacter sp. HI0075*	KZZ01663
14	14026-15303	→	425	transposase of ISL3-family	425/425 (100%)	*Proteobacteria*	WP_000610830
15	15954-15346	←	202	adenylate cyclase	138/195 (71%)	*Leifsonia sp. Leaf264*	WP_055900772
16	17137-16232	←	301	ATP-binding protein of transposon	232/301 (77%)	*Arthrobacter castelli*	WP_026820632
17	18578-17124	←	484	transposase of Tn3-family	370/484 (76%)	*Brevibacterium*	WP_051505134
18	19178-18585	←	197	recombinase of transposon	185/195 (95%)	*Arthrobacter sp. YC-RL1*	WP_047118360
19	19943-19545	←	132	transcriptional regulator, MecI family	132/132 (100%)	*Paeniglutamicibacter gangotriensis*	WP_007273052
20	20491-19940	←	183	hypothetical protein	180/183 (98%)	*Paeniglutamicibacter gangotriensis*	WP_007273053
21	20628-21452	→	274	thiol-disulfide oxidoreductase	255/274 (93%)	*Paeniglutamicibacter gangotriensis*	WP_007273054
22	21452-22360	→	302	cytochrome c biogenesis protein CcdA	301/302 (99%)	*Paeniglutamicibacter gangotriensis*	WP_007273055
23	22357-22707	→	116	intracellular sulfur oxidation protein DsrE	116/116 (100%)	*Paeniglutamicibacter gangotriensis*	WP_007273056
24	23195-22776	←	139	hypothetical protein	131/139 (94%)	*Paeniglutamicibacter gangotriensis*	WP_007273057
25	24610-23282	←	442	MFS transporter	431/442 (98%)	*Paeniglutamicibacter gangotriensis*	WP_007273058
26	25561-24665	←	298	sulfite exporter TauE/SafE	256/269 (95%)	*Paeniglutamicibacter gangotriensis*	WP_007273059
27	25921-25625	←	98	rhodanese-like domain-containing protein	98/98 (100%)	*Arthrobacter sp. UCD-GKA*	WP_071214388
28	26596-26012	←	194	hypothetical protein	190/194 (98%	*Arthrobacter sp. UCD-GKA*	WP_071214387
29	28108-26711	←	465	MBL fold metallo-hydrolase	445/465 (96%)	*Arthrobacter sp. YC-RL1*	ALQ32526
30	28459-28635	→	58	hypothetical protein	53/58 (91%)	*Arthrobacter sp. 7749*	ASN39331
31	28626-28883	→	85	transcriptional regulator, CsoR-family	85/85 (100%)	*Arthrobacter sp. YC-RL1*	ALQ32527
32	29284-29850	→	188	recombinase	177/186 (95%)	*Arthrobacter sp. UCD-GKA*	WP_071216167
33	29847-30755	→	302	hypothetical protein	122/288 (42%)	*Arthrobacter sp. ok909*	SDP64712
34	31173-32324	→	383	hypothetical protein	174/370 (47%)	*Arthrobacter sp. N199823*	WP_104111179
35	32434-33945	→	503	hypothetical protein	219/515 (43%)	*Arthrobacter sp. N199823*	WP_104111179

36	34845-34057	←	262	hypothetical protein	171/251 (68%)	*Arthrobacter sp. 162MFSha1.1*	WP_018771441
37	35766-35383	←	127	hypothetical protein	54/97 (56%)	*Arthrobacter sp. SRS-W-1-2016*	WP_078105078
38	37636-35684	←	650	hypothetical protein	353/629 (56%)	*Arthrobacter sp. SRS-W-1-2016*	WP_078105078
39	38039-37617	←	140	hypothetical protein	62/110 (56%)	*Arthrobacter sp. SRS-W-1-2016*	WP_078105079
40	38239-38949	→	236	recombinase	156/210 (74%)	*Arthrobacter sp. DWC3*	WP_104051565
41	38982-39377	→	131	hypothetical protein	70/129 (54%)	*Kocuria flava*	WP_101853274
42	39416-44437	→	1673	helicase	1187/1613 (74%)	*Arthrobacter subterraneus*	SDI69836
43	44939-44658	←	93	hypothetical protein	55/85 (65%)	*Arthrobacter sp. AK-1*	WP_012311548
44	45129-45527	→	132	mobilization protein MobC	89/130 (68%)	*Glutamicibacter arilaitensis Re117*	CBQ74062
45	45524-47188	→	554	mobilization protein, relaxase	415/564 (74%)	*Paeniglutamicibacter antarcticus*	WP_068731965
46	47231-48424	→	397	hypothetical protein	285/397 (72%)	*Paeniglutamicibacter antarcticus*	WP_068731963
47	49028-48540	←	162	hypothetical protein	82/159 (52%)	*Arthrobacter sp. Soil762*	WP_056345714
48	50194-49025	←	389	metallo-endopeptidase, ImmA/IrrE family	192/390 (49%)	*Geodermatophilus saharensis*	WP_089404652
49	51034-50309	←	241	hypothetical protein	78/114 (68%)	*Paeniglutamicibacter antarcticus*	WP_084382367
50	52850-51042	←	602	conjugal transfer protein	535/602 (89%)	*Paeniglutamicibacter antarcticus*	WP_068735628
51	54344-52851	←	497	ATP-binding protein	486/497 (98%)	*Paeniglutamicibacter antarcticus*	WP_068735626
52	55867-54359	←	502	hypothetical protein	490/502 (98%)	*Paeniglutamicibacter antarcticus*	WP_068735625
53	57432-55864	←	522	hypothetical protein	260/264 (98%)	*Paeniglutamicibacter antarcticus*	WP_068735622
54	58151-57429	←	240	hypothetical protein	162/166 (98%)	*Paeniglutamicibacter antarcticus*	WP_068735621
55	58453-58169	←	94	hypothetical protein	89/94 (95%)	*Paeniglutamicibacter antarcticus*	WP_068735618
56	59040-58450	←	196	hypothetical protein	143/196 (73%)	*Paeniglutamicibacter antarcticus*	WP_068735616
57	60467-59058	←	469	hypothetical protein	393/472 (83%)	*Paeniglutamicibacter antarcticus*	WP_068735614
58	60997-60500	←	165	hypothetical protein	132/170 (78%)	*Paeniglutamicibacter antarcticus*	WP_068735612
59	62133-60994	←	379	peptidase, M23-family	349/379 (92%)	*Paeniglutamicibacter antarcticus*	WP_068735610
60	62286-62558	→	90	transcriptional regulator, ArsR-family	56/90 (62%)	*Arthrobacter castelli*	WP_035770425
61	62711-63442	→	243	hypothetical protein	115/244 (47%)	*Arthrobacter sp. B1805*	WP_104119076
62	63442-64170	→	242	hypothetical protein	105/240 (44%)	*Arthrobacter sp. B1805*	WP_104119077
63	64243-64470	→	75	hypothetical protein	53/75 (71%)	*Arthrobacter sp. SX1312*	WP_104168933
64	64738-65721	→	327	hypothetical protein	181/327 (55%)	*Arthrobacter sp. TB 26*	WP_024366458
65	66119-66964	→	281	DNA/RNA non-specific endonuclease	183/282 (65%)	*Arthrobacter sp. MYb213*	PRB66826
66	67353-67790	→	145	hypothetical protein	41/101 (41%)	*Frankia sp. Cpl1-P*	KQM07901
67	67794-68192	→	132	transposase of IS5-family	109/112 (97%)	*Arthrobacter sp. UCD-GKA*	WP_071216986
68	69947-68514	←	477	MFS transporter	474/477 (99%)	*Arthrobacter sp. YC-RL1*	KLI90489
69	70608-70027	←	193	alkylhydroperoxidase	192/193 (99%)	*Arthrobacter sp. YC-RL1*	KLI90490
70	71509-70661	←	282	hypothetical protein	282/282 (100%)	*Arthrobacter sp. YC-RL1*	KLI90501
71	72145-71816	←	109	transcriptional regulator, CopY family	109/109 (100%)	*Arthrobacter sp. YC-RL1*	KLI90491
72	72700-72431	←	89	hypothetical protein	83/89 (93%)	*Arthrobacter sp. YC-RL1*	KLI90492
73	73130-72753	←	125	Cu(2+)-exporting ATPase, partial	119/125 (95%)	*Arthrobacter sp. YC-RL1*	WP_047117894

pA58H3							
pA58H3_p...							
01	248-1045	→	265	partitioning protein ParA	192/265 (72%)	*Kocuria marina*	KHE74185
02	1042-1404	→	120	hypothetical protein	76/120 (63%)	*Kocuria marina*	WP_035964271
03	2016-2447	→	143	DNA polymerase V subunit UmuD	131/141 (93%)	*Paeniglutamicibacter gangotriensis*	WP_007273049
04	2429-3748	→	439	DNA polymerase V subunit UmuC	412/439 (94%)	*Paeniglutamicibacter gangotriensis*	WP_007273048
05	5079-4669	←	136	hypothetical protein, partial	95/135 (70%)	*Arthrobacter sp. UCD-GKA*	WP_071216150
06	5413-5114	←	99	DNA invertase, partial	99/99 (100%)	*Glutamicibacter sp. BW77*	WP_096253936
07	5649-6020	→	123	hypothetical protein	46/126 (37%)	*Arthrobacter sp. PAO19*	WP_022875896
08	6065-7123	→	352	hypothetical protein	208/322 (65%)	*Arthrobacter sp. J3.40*	WP_015061943
09	7837-7367	←	156	hypothetical protein	-/-	*none*	-
10	8784-9392	→	202	hypothetical protein	182/202 (90%)	*Paeniglutamicibacter gangotriensis*	WP_007273036
11	10652-9462	←	396	Fic family protein	374/396 (94%)	*Paeniglutamicibacter gangotriensis*	WP_007273075
12	11069-11851	→	260	dimethylmenaquinone methyltransferase	223/257 (87%)	*Arthrobacter sp. Leaf337*	WP_055805518
13	11848-12843	→	331	tripartite tricarboxylate transporter substrate binding protein TctC	253/330 (77%)	*Arthrobacter sp. UNC362MFTsu5.1*	WP_028271781
14	12840-13340	→	166	tripartite tricarboxylate transporter TctB	105/166 (63%)	*Arthrobacter sp. Leaf337*	WP_055805524
15	13353-14867	→	504	tripartite tricarboxylate transporter TctA	447/499 (90%)	*Arthrobacter sp. Leaf337*	KQR64159
16	14879-15799	←	306	transcriptional regulator, LysR family	248/301 (82%)	*Arthrobacter sp. Leaf337*	KQR64160
17	16013-16876	→	287	transcriptional regulator, MurR/RpiR family	180/256 (70%)	*Arthrobacter sp. UNC362MFTsu5.1*	WP_081818807
18	16873-18084	→	403	MFS transporter	279/393 (71%)	*Pseudarthrobacter siccitolerans*	WP_050053759
19	20002-18656	←	448	MFS transporter	231/442 (52%)	*Rhodococcus*	WP_072936912
20	21378-20005	←	457	aldehyde dehydrogenase	251/454 (55%)	*Streptomyces sp. NBRC 109706*	WP_062213628
21	21407-22177	←	256	dehydrogenase, 3-ketoacyl-(acyl-carrier-protein) reductase	147/248 (59%)	*Arthrobacter sp. 161MFSha2.1*	WP_018780036
22	22416-23198	→	260	transcriptional regulator, IclR family	99/254 (39%)	*Actinopolyspora saharensis*	WP_092521740
23	24016-23195	←	273	transcriptional regulator, IclR family	92/238 (39%)	*Rhodococcus opacus*	AII10570
24	25009-24032	←	325	phthalate dioxygenase reductase	262/325 (81%)	*Arthrobacter sp. UNC362MFTsu5.1*	WP_028272367
25	25527-25012	←	171	hypothetical protein	136/170 (80%)	*Arthrobacter sp. 68b*	AKG47402
26	26730-25537	←	397	threonine dehydrogenase	316/389 (81%)	*Arthrobacter crystallopoietes*	WP_074703479
27	28042-26756	←	428	(2Fe-2S)-binding protein	394/428 (92%)	*Arthrobacter crystallopoietes*	WP_074703480
28	28217-29860	→	547	transcriptional regulator, IclR family	293/545 (54%)	*Arthrobacter crystallopoietes*	SDR29105
29	30300-31124	→	254	transcriptional regulator, IclR family	105/264 (40%)	*Rhodococcus pyridinivorans*	WP_094980998
30	31915-31121	←	264	transcriptional regulator, IclR family	102/225 (45%)	*Rhodococcus marinonascens*	WP_084721780
31	32314-33339	→	341	ABC transporter substrate-binding	195/332 (59%)	*Streptomyces sp. TP-A0874*	WP_079127740
32	33387-34193	→	268	ABC-type nitrate/sulfonate/bicarbonate transport system, permease component	187/260 (72%)	*Streptomyces sp. TP-A0874*	WP_079127741
33	34193-35053	→	286	ABC transporter ATP-binding protein	186/263(71%)	*Caballeronia fortuita*	WP_061133805
34	35204-36436	→	410	arabinose ABC transporter permease	161/392 (41%)	*Sinomonas atrocyanea*	WP_066499825

35	41055-37630	←	1141	restriction endonuclease subunit R, type I EcoKI enzyme	1012/1138 (89%)	*Arthrobacter sp. Leaf69*	WP_056432216
36	41417-41175	←	80	restriction endonuclease Mrr, part III	79/80 (99%)	*Arthrobacter sp. Leaf69*	WP_056431625
37	41815-41495	←	106	restriction endonuclease Mrr, part II	79/100 (79%)	*Arthrobacter sp. Leaf69*	WP_056431625
38	42110-41817	←	97	restriction endonuclease Mrr, part I	76/97 (78%)	*Arthrobacter sp. Leaf69*	WP_056431625
39	43249-42107	←	380	restriction endonuclease subunit S, type I EcoKI enzyme	238/374 (64%)	*Arthrobacter sp. 31Y*	WP_024818465
40	44790-43294	←	498	SAM-dependent DNA methyltransferase , type I RM system	442/497 (89%)	*Arthrobacter sp. Leaf69*	WP_056431620
41	43294-44790	→	323	hypothetical protein	123/172 (72%)	*Arthrobacter sp. Soil764*	WP_056332725
42	46971-46096	←	291	hypothetical protein	112/266 (42%)	*Arthrobacter sp. 9MFCol3.1*	WP_026536639
43	47261-47947	→	228	hypothetical protein	189/228 (93%)	*Glutamicibacter halophytocola*	WP_060703080
44	49446-48226	←	406	hypothetical protein	120/393 (31%)	*Demequina aurantiaca*	WP_061964237
45	49859-49443	←	138	hypothetical protein	46/134 (34%)	*Clavibacter michiganensis subsp. Michiganensis*	OUD86942
46	50671-50086	←	195	DNA resolvase	181/189 (96%)	*Arthrobacter sp. UCD-GKA*	WP_071216176
47	51571-51269	←	100	hypothetical protein	56/100 (56%)	*Paeniglutamicibacter antarcticus*	WP_068738518
48	52586-51912	←	224	hypothetical protein	73/139 (53%)	*Arthrobacter sp. B6*	WP_066286544
49	53932-52934	←	332	peptidase	192/321 (60%)	*Arthrobacter sp. UCD-GKA*	WP_071216184
50	54586-54044	←	180	hypothetical protein	93/181 (51%)	*Arthrobacter sp. 9MFCol3.1*	WP_051532949
51	55257-54928	←	109	hypothetical protein	34/85 (40%)	*Renibacterium salmoninarum*	WP_041684834
52	55374-55673	→	99	resolvase/invertase, partial	39/91 (43%)	*Arthrobacter sp. L77*	WP_052274287
53	56197-55925	←	90	hypothetical protein	-/-	*none*	-
54	56346-56609	→	87	hypothetical protein	53/84 (63%)	*Arthrobacter sp. Soil762*	WP_056343145
55	56678-56821	→	47	hypothetical protein	24/46 (52%)	*Arthrobacter sp. M2012083*	WP_026005769
56	57183-56818	←	121	hypothetical protein	59/120 (49%)	*Arthrobacter sp. Rue61a*	WP_014923458
57	57592-62076	→	1494	conjugal transfer protein TraA	920/1535 (60%)	*Arthrobacter sp. Rue61a*	WP_014923459
58	63217-62168	←	349	hypothetical protein	195/324 (60%)	*Glutamicibacter halophytocola*	WP_060700398
59	63586-63362	←	74	hypothetical protein	-/-	*none*	-
60	63708-64046	→	112	hypothetical protein	-/-	*none*	-
61	64605-65102	→	165	hypothetical protein	-/-	*none*	-
62	65648-67042	→	464	transposase, IS1380 family	355/464 (77%)	*Arthrobacter sp. U41*	WP_083266399
63	67698-67195	←	167	hypothetical protein	136/172 (79%)	*Arthrobacter sp. W1*	KWR70626
64	68991-67825	←	388	transposase, ISAs1 family	222/388 (57%)	*Arthrobacter alpinus*	WP_082357861
65	70126-69104	←	340	peptidase M23	233/304 (77%)	*Paeniglutamicibacter antarcticus*	WP_068735610
66	70880-70143	←	245	hypothetical protein	104/195 (53%)	*Arthrobacter sp. KI72*	WP_079941686
67	72745-70877	←	622	conjugal transfer protein TraG	377/592 (64%)	*Arthrobacter sp. ZXY-2*	WP_071416994
68	74333-72765	←	522	conjugal ATP-binding protein	337/517 (65%)	*Tersicoccus sp. Bi-70*	WP_083701827
69	75958-74333	←	541	hypothetical protein	340/541 (63%)	*Arthrobacter sp. Hiyo1*	GAP61402

70	77508-75958	←	516	hypothetical protein	261/481 (54%)	*Arthrobacter sp. KI72*	WP_079941690
71	78175-77510	←	221	hypothetical protein	79/220 (36%)	*Arthrobacter sp. Hiyo1*	WP_059390186
72	78540-78178	←	120	hypothetical protein	66/120 (55%)	*Arthrobacter sp. ZXY-2*	AOY74439
73	79697-78537	←	386	partitioning protein	215/382 (56%)	*Arthrobacter sp. Hiyo1*	WP_059390184
74	79862-80185	→	107	transcriptional regulator, ArsR family	75/106 (71%)	*Arthrobacter sp. UNC362MFTsu5.1*	WP_081818995
75	80305-81036	→	243	hypothetical protein	94/228 (41%)	*Arthrobacter sp. QXT-31*	WP_076798726
76	81083-81772	→	229	hypothetical protein	110/228 (48%)	*Tersicoccus sp. Bi-70*	WP_076695439
77	81904-82140	→	78	hypothetical protein	66/77 (86%)	*Arthrobacter sp. YC-RL1*	WP_082123725
78	82324-84207	→	627	hypothetical protein	330/632 (52%)	*Arthrobacter sp. KI72*	WP_079941819
				**pA19BH1**			
pA19BH1_p...							
01	300-1223	→	307	partitioning protein ParA	293/307 (95%)	*Cryobacterium arcticum*	WP_084021494
02	1220-1633	→	137	partitioning protein ParB	100/139 (72%)	*Cryobacterium arcticum*	ANP74904
03	1915-6816	→	1633	DNA damage-inducible protein, helicase	1373/1624 (85%)	*Glaciibacter superstes*	WP_022883363
04	7368-8138	→	256	hypothetical protein	99/147 (67%)	*Leifsonia sp. 71-9*	OJX78854
05	8407-9471	→	354	nucleotidyltransferase	238/352 (68%)	*Leifsonia sp. 71-9*	OJX78886
06	9459-9947	→	162	hypothetical protein	93/151 (62%)	*Leifsonia sp. 71-9*	OJX78887
07	10007-10480	→	157	hypothetical protein	80/164 (49%)	*Arthrobacter sp. TB 23*	WP_019482522
08	11248-10649	←	199	invertase/recombinase	175/196 (89%)	*Microbacterium sp. SCN 71-17*	ODT36231
09	14570-11712	←	952	NTPase	424/949 (45%)	*Candidatus Frankia californiensis*	SBW24504
10	15297-15022	←	91	hypothetical protein	78/91 (86%)	*Cryobacterium levicorallinum*	WP_092450722
11	15482-16108	→	208	transcriptional regulator, PadR family	148/212 (70%)	*Cryobacterium arcticum*	WP_084020817
12	16424-16966	→	180	hypothetical protein	91/172 (53%)	*Gulosibacter molinativorax*	WP_026935697
13	17002-21993	→	1663	Adenine-specific DNA methylase, N12 class	1057/1610 (66%)	*Agromyces sp. Soil535*	WP_056729902
14	22769-22419	←	116	hypothetical protein	34/86 (40%)	*Curtobacterium flaccumfaciens*	WP_042539564
15	22952-26758	→	1268	conjugal transfer protein TraA	737/1260 (58%)	*Rathayibacter rathayi*	SOE05907
16	28357-26828	←	509	hypothetical protein	193/501 (39%)	*Cryobacterium flavum*	WP_092341887
17	29365-28354	←	343	protein kinase	186/342 (54%)	*Microbacterium mangrovi*	WP_082008124
18	29784-30176	→	130	transcriptional regulator, XRE family	80/113 (71%)	*Cryobacterium roopkundense*	KGJ79262
19	30242-31291	→	349	hypothetical protein	273/344 (79%)	*Cryobacterium arcticum*	WP_066600569
20	31390-33144	→	584	transposase, IS30 family	435/584 (74%)	*Arthrobacter sp. U41*	WP_083266386
21	34039-34812	→	257	3-oxoacyl-[acyl-carrier-protein] reductase	167/257 (65%)	*Rugosibacter aromaticivorans*	AJP47628
22	35799-34891	←	302	extradiol ring-cleavage dioxygenase	283/302 (94%)	*Micrococcales bacterium 32-70-13*	OYX57909
23	36213-36632	←	139	PIN domain-containing protein	126/138 (91%)	*Cryobacterium sp. MLB-32*	WP_051973804
24	37168-37794	→	208	transcriptional regulator, TetR/AcrR family	134/208 (64%)	*Arthrobacter sp. U41*	WP_083266626
25	37791-39416	→	541	drug resistance transporter	469/529 (89%)	*Arthrobacter sp. Leaf69*	WP_056426022
26	39481-40110	→	209	transcriptional regulator, TetR/AcrR family	193/209 (92%)	*Pseudarthrobacter phenanthrenivorans*	WP_013602978
27	40457-40966	→	169	partial transposase of IS21 family, part 1	104/126 (83%)	*Cryobacterium flavum*	SDN36336

28	40860-41405	→	181	partial transposase of IS21 family, part 2	86/93 (93%)	*Arthrobacter sp. H20*	WP_081743327
29	42502-41573	←	309	NADP-dependent oxidoreductase	193/310 (62%)	*Gordonia sp. HS-NH1*	WP_055476403
30	42606-43247	→	213	transcriptional regulator, TetR/AcrR family	140/202 (69%)	*Gordonia rubripertincta*	ASR05659
31	43287-43838	→	183	glutathione peroxidase	130/158 (82%)	*Cellulomonas sp. KRMCY2*	WP_024286154
32	44244-43843	←	133	partial, MFS transporter	78/95 (82%)	*Herbiconiux ginsengi*	WP_092552320
33	45042-44329	←	237	succinoglycan biosynthesis protein ExoA	127/232 (55%)	*Micrococcales bacterium 72-143*	OJX66848
34	45943-45122	←	273	lysophospholipase	153/219 (70%)	*Kocuria sp. WN036*	WP_095650796
35	46842-46267	←	191	transcriptional regulator, TetR/AcrR family	96/187 (51%)	*Streptomyces mirabilis*	WP_037718496
36	46990-49395	→	801	drug exporter, membrane protein YdfJ	507/761 (67%)	*Micrococcales bacterium 32-70-13*	OYX57908
37	50001-49759	←	80	hypothetical protein	56/78 (72%)	*Cryobacterium luteum*	WP_092111469
38	50261-50040	←	73	hypothetical protein	65/70 (93%)	*Cryobacterium psychrotolerans*	WP_092324200
39	52267-50549	←	572	conjugal transfer protein TraD/TraG	232/503 (46%)	*Arthrobacter sp. ZXY-2*	WP_071416994
40	53700-52270	←	476	conjugal transfer ATPase	242/466 (52%)	*Leifsonia sp. NCR5*	WP_085370153
41	55313-53697	←	538	conjugal transfer ATP-binding protein TraC	297/492 (60%)	*Microbacterium mangrove*	WP_052226535
42	56809-55313	←	498	conjugal transfer membrane protein	287/498 (58%)	*Microbacterium sp. SCN 69-37*	ODT25947
43	58214-56826	←	462	conjugal transfer protein	179/427 (42%)	*Microbacterium sp. SCN 69-37*	ODT25991
44	58518-58216	←	100	hypothetical protein	49/93 (53%)	*Microbacterium trichothecenolyticum*	WP_045299576
45	59147-58542	←	201	murein hydrolase activator EnvC precursor	72/134 (54%)	*Cnuibacter physcomitrellae*	WP_085021889
46	59911-59150	←	253	hypothetical protein	87/239 (36%)	*Microbacterium sediminis*	WP_067028783
47	60191-60514	→	107	transcriptional regulator, ArsR family	46/107 (43%)	*Arthrobacter sp. 31Cvi3.1E*	WP_079581666
48	60573-60809	→	78	hypothetical protein	41/78 (53%)	*Herbiconiux ginseng*	WP_092558271
49	61054-62889	→	611	hypothetical protein	439/610 (72%)	*Cryobacterium flavum*	SDO33730
50	63199-63621	→	140	hypothetical protein	103/136 (76%)	*Cryobacterium luteum*	WP_092111893
51	64993-63875	←	372	hypothetical protein	268/369 (73%)	*Cryobacterium roopkundense*	WP_035839023
52	65249-66832	→	527	transposase, ISNCY family	463/525 (88%)	*Cryobacterium sp. MLB-32*	WP_051973528
53	66829-67599	→	256	ATP-binding protein	240/256 (94%)	*Cryobacterium sp. MLB-32*	KFF59070
54	67667-69343	→	558	hypothetical protein	425/557 (76%)	*Cryobacterium sp. MLB-32*	WP_051973529
				**pA44BH1**			
pA44BH1_p...							
01	453-1268	→	271	partitioning protein ParA	231/370 (86%)	*Arthrobacter*	WP_069694809
02	1273-1689	→	138	partitioning protein ParB	113/141 (85%)	*Arthrobacter*	WP_083261974
03	2121-2804	→	227	hypothetical protein	124/227 (55%)	*Arthrobacter alpinus*	WP_062008985
04	2951-3565	→	204	KfrA protein	176/207 (85%)	*Arthrobacter alpinus*	WP_062008987
05	4127-3804	←	107	hypothetical protein, partial	83/107 (78%)	*Arthrobacter crystallopoietes*	WP_074698543
06	5692-4400	←	430	transposase, ISL3 family	266/431 (62%)	*Janibacter terrae*	WP_032492314
07	7620-6118	←	500	haloacid dehalogenase (HAD)	369/484 (76%)	*Arthrobacter sp. SRS-W-1-2016*	WP_078106477
08	8058-8765	→	235	resolvase/invertase/recombinase	165/207 (80%)	*Arthrobacter sp. H5*	WP_081746535
09	8801-9196	→	131	hypothetical protein	71/131 (54%)	*Arthrobacter sp. H5*	WP_035775518

10	9292-14256	→	1654	adenine-specific DNA methylase, N12 class	1183/1639 (72%)	*Arthrobacter subterraneus*	SDI69836
11	15094-14705	←	129	hypothetical protein	71/96 (74%)	*Arthrobacter sp. SRS-W-1-2016*	WP_078106456
12	15343-15735	→	130	mobilization protein MobC	115/130 (88%)	*Arthrobacter sp. 31.31*	WP_015061717
13	15735-17294	→	519	mobilization protein, relaxase	466/519 (90%)	*Arthrobacter sp. TB 26*	WP_024366420
14	17312-18424	→	370	hypothetical protein colicin imp	223/371 (60%)	*Arthrobacter nitrophenolicus*	WP_009359335
15	18874-19770	→	298	DNA methyltransferase	207/281 (74%)	*Microbacterium trichothecenolyticum*	WP_045296087
16	19883-21655	→	590	restriction enzyme subunit McrB	294/551 (53%)	*Microbacterium sp. Cr-K29*	WP_029260290
17	21645-22958	→	437	restriction endonuclease McrBC, regulatory subunit McrC	246/430 (57%)	*Microbacterium sp. Cr-K29*	WP_029260291
18	24014-23100	←	304	hypothetical protein	83/247 (34%)	*Nocardioides sp. Leaf285*	WP_056862662
19	24188-25216	→	342	hypothetical protein	280/342 (82%)	*Arthrobacter crystallopoietes*	WP_083339472
20	25765-25349	←	138	hypothetical protein	115/140 (82%)	*Arthrobacter sp. J3-37*	WP_015062378
21	27527-25758	←	589	conjugal transfer protein TraG	529/589 (90%)	*Arthrobacter sp. SRS-W-1-2016*	WP_078106445
22	29119-27527	←	530	ATP/GTP-binding protein	503/530 (95%)	*Arthrobacter sp. TB 26*	WP_024366415
23	30630-29119	←	503	hypothetical membrane protein	478/503 (95%)	*Arthrobacter sp. TB 26*	WP_024366414
24	31975-30632	←	447	hypothetical protein	312/447 (70%)	*Arthrobacter sp. Soil764*	WP_056331991
25	32703-31975	←	242	hypothetical protein	172/244 (70%)	*Arthrobacter sp. 31.31*	WP_015061728
26	33002-32715	←	95	hypothetical protein	78/95 (82%)	*Arthrobacter sp. TB 26*	WP_024366463
27	33640-33017	←	207	hypothetical protein	166/207 (80%)	*Arthrobacter sp. TB 26*	WP_024366462
28	33755-34018	→	87	DNA-binding protein	73/87 (84%)	*Arthrobacter sp. TB 26*	WP_024366461
29	34050-35117	→	355	amidase	296/347 (85%)	*Arthrobacter sp. 31.31*	WP_015061732
30	35129-35647	→	172	hypothetical protein	148/172 (86%)	*Arthrobacter sp. TB 26*	WP_024366460
31	35661-37094	→	477	partitioning protein ParA	407/471 (86%)	*Arthrobacter sp. TB 26*	WP_024366459
32	37152-37382	→	76	hypothetical protein	60/76 (79%)	*Arthrobacter sp. J3-37*	WP_015062388
33	37500-38768	→	422	hypothetical protein	247/385 (64%)	*Arthrobacter sp. Chr15*	WP_012311476
34	39453-38794	←	199	hypothetical protein	108/184 (59%)	*Arthrobacter sp. A3*	WP_058945698
35	40029-39736	←	97	hypothetical protein	56/97 (58%)	*Arthrobacter alpinus*	WP_074710841
36	40413-40676	→	87	NrdH-redoxin	62/85 (73%)	*Sinomonas mesophila*	WP_077490863
				**pA48BH1**			
pA48BH1_p...							
01	441-1256	→	271	partitioning protein ParA	231/370 (86%)	*Arthrobacter*	WP_069694809
02	1261-1677	→	138	partitioning protein ParB	113/141 (85%)	*Arthrobacter*	WP_083261974
03	2109-2792	→	227	hypothetical protein	124/227 (55%)	*Arthrobacter alpinus*	WP_062008985
04	2939-3553	→	204	KfrA protein	176/207 (85%)	*Arthrobacter alpinus*	WP_062008987
05	4115-3792	←	107	hypothetical protein, partial	83/107 (78%)	*Arthrobacter crystallopoietes*	WP_074698543
06	5680-4388	←	430	transposase, ISL3 family	266/431 (62%)	*Janibacter terrae*	WP_032492314
07	6945-6106	←	279	haloacid dehalogenase, truncated	188/263 (71%)	*Arthrobacter sp. SRS-W-1-2016*	WP_078106477
08	7608-6970	←	212	haloacid dehalogenase, truncated	169/212 (80%)	*Arthrobacter sp. SRS-W-1-2016*	WP_078106477

09	8088-8753	→	235	resolvase/invertase/recombinase	165/207 (80%)	*Arthrobacter sp. H5*	WP_081746535
10	8789-9184	→	131	hypothetical protein	71/131 (54%)	*Arthrobacter sp. H5*	WP_035775518
11	9223-14244	→	1654	adenine-specific DNA methylase	1183/1639 (72%)	*Arthrobacter subterraneus*	SDI69836
12	15082-14693	←	129	hypothetical protein	71/96 (74%)	*Arthrobacter sp. SRS-W-1-2016*	WP_078106456
13	15331-15723	→	130	mobilization protein MobC	115/130 (88%)	*Arthrobacter sp. 31.31*	WP_015061717
14	15723-17282	→	519	mobilization protein, relaxase	466/519 (90%)	*Arthrobacter sp. TB 26*	WP_024366420
15	17300-18412	→	370	hypothetical protein colicin imp	223/371 (60%)	*Arthrobacter nitrophenolicus*	WP_009359335
16	18844-19758	→	298	DNA methylase	207/281 (74%)	*Microbacterium trichothecenolyticum*	WP_045296087
17	19871-21643	→	590	restriction enzyme subunit McrB	294/551 (53%)	*Microbacterium sp. Cr-K29*	WP_029260290
18	21633-22946	→	437	restriction endonuclease McrBC, regulatory subunit McrC	246/430 (57%)	*Microbacterium sp. Cr-K29*	WP_029260291
19	24002-23088	←	304	hypothetical protein	83/247 (34%)	*Nocardioides sp. Leaf285*	WP_056862662
20	24176-25204	→	342	hypothetical protein	280/342 (82%)	*Arthrobacter crystallopoietes*	WP_083339472
21	25753-25337	←	138	hypothetical protein	115/140 (82%)	*Arthrobacter sp. J3-37*	WP_015062378
22	27515-25746	←	589	conjugal transfer protein TraG	529/589 (90%)	*Arthrobacter sp. SRS-W-1-2016*	WP_078106445
23	29107-27515	←	530	ATP/GTP-binding protein	503/530 (95%)	*Arthrobacter sp. TB 26*	WP_024366415
24	30618-29107	←	503	hypothetical membrane protein	478/503 (95%)	*Arthrobacter sp. TB 26*	WP_024366414
25	31963-30620	←	447	hypothetical protein	312/447 (70%)	*Arthrobacter sp. Soil764*	WP_056331991
26	32691-31963	←	242	hypothetical protein	172/244 (70%)	*Arthrobacter sp. 31.31*	WP_015061728
27	32990-32703	←	95	hypothetical protein	78/95 (82%)	*Arthrobacter sp. TB 26*	WP_024366463
28	33628-33005	←	207	hypothetical protein	166/207 (80%)	*Arthrobacter sp. TB 26*	WP_024366462
29	33743-34006	→	87	DNA-binding protein	73/87 (84%)	*Arthrobacter sp. TB 26*	WP_024366461
30	34038-35105	→	355	amidase	296/347 (85%)	*Arthrobacter sp. 31.31*	WP_015061732
31	35117-35635	→	172	hypothetical protein	148/172 (86%)	*Arthrobacter sp. TB 26*	WP_024366460
32	35649-37082	→	477	partitioning protein ParA	407/471 (86%)	*Arthrobacter sp. TB 26*	WP_024366459
33	37140-37370	→	76	hypothetical protein	60/76 (79%)	*Arthrobacter sp. J3-37*	WP_015062388
34	37488-38756	→	422	hypothetical protein	247/385 (64%)	*Arthrobacter sp. Chr15*	WP_012311476
35	39381-38782	←	199	hypothetical protein	108/184 (59%)	*Arthrobacter sp. A3*	WP_058945698
36	40017-39724	←	97	hypothetical protein	56/97 (58%)	*Arthrobacter alpinus*	WP_074710841
37	40399-40662	→	87	NrdH-redoxin	62/85 (73%)	*Sinomonas mesophila*	WP_077490863
