# Supplementary material for: Insight Into the Diversity and Possible Role of Plasmids in the Adaptation of Psychrotolerant and Metalotolerant Arthrobacter spp. to Extreme Antarctic Environments
Source: Front Microbiol. 2018 Dec 18;9:3144. doi: 10.3389/fmicb.2018.03144 (PMC6305408; doi:10.3389/fmicb.2018.03144)
Supplement: Supplementary file 4 [file Table_4.pdf]

Supplementary Material

Insight into the Diversity and Possible Role of Plasmids in the Adaptation of Psychrotolerant and Metalotolerant *Arthrobacter* spp. to Extreme Antarctic Environments

Krzysztof Romaniuk, Piotr Golec, Lukasz Dziewit*
*** Correspondence:** Dr. Lukasz Dziewit: ldziewit@biol.uw.edu.pl

TABLE S4. Partitioning systems (including sequences of predicted partitioning sites – *parS*) identified within the ANT plasmids.

Plasmid name	*par* genes (coordinates)	Sequences and coordinates of putative direct repeats (DR) of *parS* site [a]	Homologous PAR system (found based on the best BLASTp hit to the ParA protein)
pA2H2	*pA2H2_49-50* (51,902 – 52,672)	DR.1:51851-ATGCAA-51856<br>DR.2:51871-ATGAAA-51876<br>DR.3:51879-ATGAAA-51884<br>DR.4:51902-ATGCAA-51907<br><br>Consensus: ATGMAA	*Arthrobacter* sp. MA-N2 [GenBank: WP_028268205 and WP_028268206]
pA8H1	*pA8H1_01-02* (78 – 961)	DR.1: 5-CACTTACTCATCTACT-20<br>DR.2:46-CATTTACTCACCCTCT-61<br><br>Consensus: CAYTTACTCAYCYWCT	*Glutamicibacter* sp. BW77 [GenBank: WP_096253931 and WP_096253932]
pA19BH1	*pA19BH1_01-02* (300 – 1,633)	DR.1:198-CAGTAAAAGCAGCA-211<br>DR.2:216-CAGCAAAAGCAGCA-229<br>DR.3:252-CAGCATTAGCTGTA-265<br><br>Consensus: CAGYAWWAGCWGYA	*Cryobacterium arcticum* plasmid pP27867_2 [GenBank: ANP74904 and ANP74905]
pA40H1	*pA40H1_01-02* (90 – 1,138)	DR.1:27-AATCTTTC-34<br>DR.2:39-AATCTTTC-46<br>DR.3:63-AATCTTTC-70<br><br>Consensus: AATCTTTC	*Arthrobacter* sp. MA-N2 [GenBank: WP_028268205 and WP_028268206]
pA40H2	*pA40H2_01-02* (241 – 1,397)	DR.1:174-CAAGTAATGC-183<br>DR.2:201-CAAGTAATAC-210<br>DR.3:219-CAAGTACTAC-228<br><br>Consensus: CAAGTAMTRC	*Kocuria marina* [GenBank: KHE74185 and KHE74186]
pA44BH1	*pA44BH1_01-02* (453 – 1,689)	DR.1:383-GTGGAATGGC-392<br>DR.2:393-GTGGAATTGC-402<br><br>Consensus: GTGGAATKGC	*Arthrobacter* sp. 31.31 [GenBank: WP_015061701 and WP_015061702]
pA48BH1	*pA48BH1_01-02* (441 – 1,677)	DR.1:371-GTGGAATGGC-380<br>DR.2:381-GTGGAATTGC-390<br><br>Consensus: GTGGAATKGC	*Arthrobacter* sp. 31.31 [GenBank: WP_015061701 and WP_015061702]
pA58H1	*pA58H1_01-02* (172 – 1,622)	DR.1:127-AATCAGCA-134<br>DR.2:146-AATCACCA-153<br><br>Consensus: AATCASCA	*Microbacterium esteraromaticum* [GenBank: WP_087130340 and WP_087130341]
pA58H2	*pA58H2_01-02* (276 – 1,046)	DR2.1:181-CTTGACGTC-189<br>DR2.2:208-CTTACTTTC-216<br>DR2.2:240-CTTGCTATC-248<br><br>Consensus: CTTRMYDTC	*Kocuria polaris* [GenBank: KHD96206 and KHD96206]
pA58H3	*pA58H3_01-02* (248 – 1,404)	DR.1:174-CAAGTAATGC-183<br>DR.2:208-CAAGTAATAC-217<br>DR.3:226-CAAGTACTAC-235<br><br>Consensus: CAAGTAMTRC	*Kocuria marina* [GenBank: KHE74185 and KHE74186]

[a] Sequences shown in the 5′ to 3′ orientation.
