# Supplementary material for: Insight Into the Diversity and Possible Role of Plasmids in the Adaptation of Psychrotolerant and Metalotolerant Arthrobacter spp. to Extreme Antarctic Environments
Source: Front Microbiol. 2018 Dec 18;9:3144. doi: 10.3389/fmicb.2018.03144 (PMC6305408; doi:10.3389/fmicb.2018.03144)
Supplement: Supplementary file 5 [file Table_5.pdf]

Supplementary Material

Insight into the Diversity and Possible Role of Plasmids in the Adaptation of Psychrotolerant and Metalotolerant *Arthrobacter* spp. to Extreme Antarctic Environments

Krzysztof Romaniuk, Piotr Golec, Lukasz Dziewit*

*** Correspondence:** Dr. Lukasz Dziewit: ldziewit@biol.uw.edu.pl

TABLE S5. Summary of phenotypic modules found within the ANT plasmids.

Plasmid name	Protein	Predicted protein function [Enzyme commission number]
Protection against UV radiation		
pA2H2	pA2H2_p51,<br>pA2H2_p59	DNA-directed DNA polymerase subunit UmuD, COG1974<br>DNA-directed DNA polymerase subunit UmuC, COG0389 [EC: 2.7.7.7]
pA8H1	pA8H1_p03,<br>pA8H1_p21	DNA-directed DNA polymerase subunit UmuD, COG1974<br>DNA-directed DNA polymerase subunit UmuC, COG0389 [EC: 2.7.7.7]
pA40H2	pA40H2_p03<br>pA40H2_p11	DNA-directed DNA polymerase subunit UmuD, COG1974<br>DNA-directed DNA polymerase subunit UmuC, COG0389 [EC: 2.7.7.7]
pA58H3	pA58H3_p03<br>pA58H3_p04	DNA-directed DNA polymerase subunit UmuD, COG1974<br>DNA-directed DNA polymerase subunit UmuC, COG0389 [EC: 2.7.7.7]
Uptake and utilisation of various organic compounds		
pA2H2	pA2H2_p16<br>pA2H2_p17<br>pA2H2_p18	ABC-type lipoprotein export system, ATPase component, COG1136 [EC: 3.6.3.28]<br>ABC-type antimicrobial peptide transport system, permease component, COG0577<br>TetR family transcriptional regulator
pA2H2	pA2H2_p20<br>pA2H2_p21<br>pA2H2_p22<br>pA2H2_p23	MMPL family transporter<br>TetR/AcrR family transcriptional regulator, COG1309<br>drug resistance transporter, EmrB/QacA subfamily<br>TetR family transcriptional regulator
pA8H1	pA8H1_p12	laminarinase, glycoside hydrolase, COG2273
pA58H3	pA58H3_p13<br>pA58H3_p14<br>pA58H3_p15<br>pA58H3_p16	tripartite tricarboxylate transporter substrate binding protein, COG3181<br>tripartite tricarboxylate transporter TctB<br>tripartite tricarboxylate transporter permease, TctA, COG3333<br>LysR family transcriptional regulator, COG0583
pA58H3	pA58H3_p17<br>pA58H3_p18	major facilitator superfamily transporter<br>MurR/RpiR family transcriptional regulator, COG1737
pA58H3	pA58H3_p19<br>pA58H3_p20<br>pA58H3_p21<br>pA58H3_p22<br>pA58H3_p23<br>pA58H3_p24<br>pA58H3_p25<br>pA58H3_p26<br>pA58H3_p27	NAD(P)-dependent dehydrogenase, COG1028 [EC: 1.1.1.-]<br>aldehyde dehydrogenase, COG1022, [EC: 1.2.1.3]<br>major facilitator superfamily transporter, COG2814<br>IclR family transcriptional regulator, COG1414<br>IclR family transcriptional regulator, COG1414<br>Phthalate 4,5-dioxygenase, COG1018 [EC: 1.14.12.7]<br>hypothetical protein<br>L-threonine 3-dehydrogenase, COG1063 [EC: 1.1.1.103]<br>aromatic ring-hydroxylating dioxygenase subunit alpha, COG4638 [EC: 1.14.12.7]
pA58H3	pA58H3_p32<br>pA58H3_p33<br>pA58H3_p34<br>pA58H3_p35	taurine transporter substrate binding subunit TauA, COG0715<br>taurine ABC transporter permease TauC, COG0600 [EC: 3.6.3.31]<br>taurine transporter TauB, COG:1116<br>arabinose ABC transporter permease

pA19BH1	pA19BH1_p21<br>pA19BH1_p22	short-chain dehydrogenase, COG1028<br>Aromatic ring-opening dioxygenase, catalytic subunit, COG3384 [EC: 1.13.11.29]
pA19BH1	pA19BH1_p24<br>pA19BH1_p25<br>pA19BH1_p26<br>pA19BH1_p36	TetR family transcriptional regulator<br>drug resistance transporter, EmrB/QacA subfamily<br>TetR/AcrR family transcriptional regulator, COG1309<br>MMPL family transporter
Transport of heavy metals		
pA2H2	pA2H2_p55<br>pA2H2_p56	tellurium resistance protein TerC, COG0861<br>putative cation transporting ATPase
pA8H1	pA8H1_p16	tellurium resistance protein TerC, COG0861,
pA8H1	pA8H1_p07<br>pA8H1_p08	tellurium resistance protein TerC, COG0861<br>putative cation transporting ATPase
pA40H2	pA40H2_p07<br>pA40H2_p08	tellurium resistance protein TerC, COG0861<br>putative cation transporting ATPase
pA40H1	pA40H1_p55<br>pA40H1_p56<br>pA40H1_p57	copper chaperone CopZ, COG2608<br>heavy metal-binding domain-containing protein<br>copper-(or silver)-translocating P-type ATPase, COG2217 [EC: 3.6.3.53]
Sulfur detoxification and energy acquisition		
pA8H1	pA8H1_p33<br>pA8H1_p34	SulP family inorganic anion transporter, sulfate permease, COG0659<br>MerR family DNA-binding transcriptional regulator, COG0789
pA8H1	pA8H1_p35<br>pA8H1_p36<br>pA8H1_p37<br>pA8H1_p38<br>pA8H1_p39<br>pA8H1_p40<br>pA8H1_p41<br>pA8H1_p42	FrmR family DNA-binding transcriptional regulator, COG1937<br>hypothetical protein<br>persulfide dioxygenase A, COG0491 [EC: 1.13.11.18]<br>rhodanese-related sulfurtransferase, COG0607<br>rhodanese-related sulfurtransferase, COG0607<br>sulfite exporter TauE/SafE, COG0730<br>major facilitator superfamily transporter<br>hypothetical protein, uncharacterized conserved protein, COG3439
pA58H2	pA58H2_p19<br>pA58H2_p20<br>pA58H2_p21<br>pA58H2_p22<br>pA58H2_p23	intracellular sulfur oxidation, DsrE/DsrF, COG1416<br>cytochrome C biogenesis protein transmembrane region, COG0785<br>thiol-disulfide oxidoreductase, COG1651<br>Cytochrome c-type biogenesis protein CcdA<br>predicted transcriptional regulator, COG3682
pA58H2	pA58H2_p24<br>pA58H2_p25<br>pA58H2_p26<br>pA58H2_p27<br>pA58H2_p28<br>pA58H2_p29<br>pA58H2_p30<br>pA58H2_p31	hypothetical protein, uncharacterized conserved protein,<br>major facilitator superfamily transporter<br>sulfite exporter TauE/SafE, COG0730<br>rhodanese-related sulfurtransferase, COG0607<br>rhodanese-related sulfurtransferase, COG0607<br>persulfide dioxygenase A, COG0491 [EC: 1.13.11.18]<br>hypothetical protein<br>COG3439FrmR family DNA-binding transcriptional regulator, COG1937
pA40H2	pA40H2_p27<br>pA40H2_p28	SulP family inorganic anion transporter, sulfate permease, COG0659<br>MerR family DNA-binding transcriptional regulator, COG0789
pA40H2	pA40H2_p29<br>pA40H2_p30<br>pA40H2_p31<br>pA40H2_p32<br>pA40H2_p33<br>pA40H2_p34<br>pA40H2_p35<br>pA40H2_p36	FrmR family DNA-binding transcriptional regulator, COG1937<br>hypothetical protein<br>persulfide dioxygenase A, COG0491 [EC: 1.13.11.18]<br>rhodanese-related sulfurtransferase, COG0607<br>rhodanese-related sulfurtransferase, COG0607<br>sulfite exporter TauE/SafE, COG0730<br>major facilitator superfamily transporter<br>hypothetical protein, uncharacterized conserved protein, COG3439
pA40H2	pA58H2_p37<br>pA58H2_p38<br>pA58H2_p39<br>pA58H2_p40<br>pA58H2_p41	predicted transcriptional regulator, COG3682<br>Cytochrome c-type biogenesis protein CcdA<br>thiol-disulfide oxidoreductase, COG1651<br>cytochrome C biogenesis protein transmembrane region, COG0785<br>intracellular sulfur oxidation protein, DsrE/DsrF, COG1416
