# Supplementary material for: Insight Into the Diversity and Possible Role of Plasmids in the Adaptation of Psychrotolerant and Metalotolerant Arthrobacter spp. to Extreme Antarctic Environments
Source: Front Microbiol. 2018 Dec 18;9:3144. doi: 10.3389/fmicb.2018.03144 (PMC6305408; doi:10.3389/fmicb.2018.03144)
Supplement: Supplementary file 6 [file Table_6.pdf]

Supplementary Material

Insight into the Diversity and Possible Role of Plasmids in the Adaptation of Psychrotolerant and Metalotolerant *Arthrobacter* spp. to Extreme Antarctic Environments

Krzysztof Romaniuk, Piotr Golec, Lukasz Dziewit*
*** Correspondence:** Dr. Lukasz Dziewit: ldziewit@biol.uw.edu.pl

TABLE S6. Distribution of the genetic modules carried by the ANT plasmids in various *Arthrobacter* genomes.

Strain (chromosome or plasmid)	GenBank acc. no	Genetic modules:													
		AMI	ARM	COP	CYT	EFX	LAM	LIP	MFS	SLP	SUL	TAU	TCT	TER	UMU
Arthrobacter agilis UMCV2	CP024915	-	-	+	-	-	-	+	-	+	-	-	-	+	+
Arthrobacter alpinus A3	CP013745	+	-	+	-	+	-	+	-	+	-	-	-	+	+
plasmid unnamed	CP013746	-	-	-	-	-	-	-	-	-	-	-	-	-	+
Arthrobacter alpinus R3.8	CP012677	+	-	+	+	+	-	-	-	+	+	-	-	+	+
Arthrobacter alpinus ERGS4:06	CP013200	+	-	+	-	+	-	+	-	+	-	-	-	+	+
plasmid pRK01	CP013201	-	-	-	-	-	-	-	-	-	-	-	-	-	-
Arthrobacter crystallopoietes DSM 20117	CP018863	+	-	+	+	+	-	-	+	+	+	-	+	+	+
plasmid pLDW-9	CP018864	-	-	-	-	-	-	-	-	-	-	-	-	-	-
plasmid pLDW-10	CP018865	-	+	-	+	-	-	-	-	-	+	-	-	+	-
Arthrobacter sp. 7749	CP022462	+	-	+	-	+	-	+	+	+	+	-	+	+	+
Arthrobacter sp. ATCC 21022	CP014196	+	+	+	-	-	-	+	+	+	+	-	-	+	+
Arthrobacter sp. DCT-5	CP029642	+	-	-	-	+	-	-	-	-	-	-	-	-	-
plasmid pDCT5	CP029643	-	-	-	-	-	-	-	-	-	-	-	-	-	-
Arthrobacter sp. ERGS1:01	CP012479	+	-	+	-	+	-	+	-	+	-	-	-	+	+
plasmid unnamed 1	CP012478	-	-	-	-	-	-	-	-	-	-	-	-	-	-
plasmid unnamed 2	CP012477	-	-	-	-	-	-	-	-	-	-	-	-	-	-
Arthrobacter sp. FB24	CP000454	+	-	+	-	+	-	-	+	+	+	-	+	+	+
plasmid 1	CP000455	-	-	+	-	-	-	-	-	-	-	-	-	-	-
plasmid 2	CP000456	-	-	-	-	-	-	-	-	-	-	-	-	-	-
plasmid 2	CP000457	-	-	-	-	-	-	-	-	-	-	-	-	-	-
Arthrobacter sp. Hiyo4	AP014718	-	-	+	+	-	-	-	-	+	+	-	-	+	-
Arthrobacter sp. PAMC 25486	CP007595	+	-	+	-	+	-	-	-	+	-	-	-	+	+
Arthrobacter sp. PGP41	CP026514	-	-	+	-	-	-	-	+	-	+	-	-	+	+
Arthrobacter sp. PM3	CP022314	+	-	+	-	+	-	+	+	+	+	-	-	-	-
Arthrobacter sp. QXT-31	CP019304	-	-	+	-	-	-	-	+	+	+	-	+	+	-
Arthrobacter sp. Rue61a	CP003203	+	-	+	-	+	-	+	+	+	+	-	-	+	+
plasmid p232	CP003204	-	-	-	-	-	-	-	-	-	+	-	-	-	-
plasmid pAL1	CP003205	-	-	-	-	-	-	-	-	-	-	-	-	-	+
Arthrobacter sp. U41	CP015732	+	-	+	+	+	-	-	-	+	+	-	+	-	+
plasmid unnamed 1	CP015733	-	-	-	-	-	-	-	-	-	-	-	-	-	-
plasmid unnamed 2	CP015734	-	-	-	-	-	-	-	-	-	-	-	-	+	+

plasmid unnamed 3	CP015735	-	-	-	-	-	-	-	-	-	-	-	-	-	-
Arthrobacter sp. YC-RL1	CP013297	+	-	+	+	+	-	-	+	+	-	-	-	+	+
plasmid unnamed 1	CP013298	-	-	-	-	-	-	-	-	-	+	-	-	-	-
plasmid unnamed 2	CP013299	-	-	-	-	-	-	-	-	-	-	-	-	-	-
Arthrobacter sp. YN	CP022436	-	-	+	-	+	-	+	+	+	-	-	-	+	+
Arthrobacter sp. ZXY-2	CP017421	+	-	+	-	-	-	+	+	+	+	-	-	+	+
plasmid pZXY21	CP017422	-	-	-	-	-	-	-	-	-	+	-	-	-	-
plasmid pZXY22	CP017423	-	-	-	-	-	-	-	-	-	-	-	-	-	-
plasmid pZXY23	CP017424	-	-	-	-	-	-	-	-	-	-	-	-	-	-
plasmid pZXY24	CP017425	-	-	-	-	-	-	-	-	-	-	-	-	-	-
plasmid pZXY25	CP017426	-	-	-	-	-	-	-	-	-	-	-	-	-	-
**Plasmids originating from strains with not sequenced chromosomes:**															
(*Arthrobacter rhombi*) plasmid pPRH	HQ624979	-	-	-	-	-	-	-	-	-	-	-	-	-	-
(*Arthrobacter* sp. 31.31) plasmid p3132-53	JQ418520	-	-	-	-	-	-	-	-	-	-	-	-	-	-
(*Arthrobacter* sp. AK-1) plasmid pSI-1	EF495211	-	-	-	-	-	-	-	-	-	-	-	-	-	-
(*Arthrobacter* sp. Chr15) plasmid pChr15	EF495212	-	-	-	-	-	-	-	-	-	-	-	-	-	-
(*Arthrobacter* sp. Hiyo8) plasmid pHiyo8-1	AP014720	-	-	+	-	-	-	-	-	-	+	-	-	-	-
(*Arthrobacter* sp. Hiyo8) plasmid pHiyo8-2	AP014721	-	-	-	-	-	-	-	-	-	-	-	-	-	-
(*Arthrobacter* sp. J3.37) plasmid pJ337-114	JQ418527	-	-	-	-	-	-	-	-	-	-	-	-	-	-
(*Arthrobacter* sp. J3.40) plasmid pJ340-69	JQ418528	-	-	-	-	-	-	-	-	-	-	-	-	-	-
(*Arthrobacter* sp. J3.40) plasmid pJ340-114	JQ418529	-	-	-	-	-	-	-	-	-	-	-	-	-	-
(*Arthrobacter* sp. J3.49) plasmid pJ349-116	JQ418530	-	-	-	-	-	-	-	-	-	-	-	-	-	-
(*Arthrobacter* sp. J3.53) plasmid pJ353-116	JQ418531	-	-	-	-	-	-	-	-	-	-	-	-	-	-
