# Supplementary material for: Insight Into the Diversity and Possible Role of Plasmids in the Adaptation of Psychrotolerant and Metalotolerant Arthrobacter spp. to Extreme Antarctic Environments
Source: Front Microbiol. 2018 Dec 18;9:3144. doi: 10.3389/fmicb.2018.03144 (PMC6305408; doi:10.3389/fmicb.2018.03144)
Supplement: Supplementary file 7 [file Table_7.pdf]

Supplementary Material

Insight into the Diversity and Possible Role of Plasmids in the Adaptation of Psychrotolerant and Metalotolerant *Arthrobacter* spp. to Extreme Antarctic Environments

Krzysztof Romaniuk, Piotr Golec, Lukasz Dziewit*

*** Correspondence:** Dr. Lukasz Dziewit: ldziewit@biol.uw.edu.pl

TABLE S7. Summary of plasmids (of cold-active bacteria) that carry phenotypic modules of adaptive value.

	**Protection against:**			**Cell mobility**		**Resistance to:**		**Protection against exogenous DNA**	
	**low temperature**	**oxidative stress**	**UV radiation**	**gas vesicles production**	**flagella formation**	**antibiotics**	**heavy metals**	**restriction-modification system**	**CRISPR-Cas system**
***Acinetobacter* (16)**	CP019151	CP014652<br>CP014653<br>CP019144			CP015111		CP019144	CP014652	
***Aeromonas* (4)**									
***Aliivibrio* (6)**	NC_011311								
***Altererythrobacter* (1)**		CP011453							
***Alteromonas* (1)**		CP013927					CP013927		
***Arthrobacter* (16)**	CP015733	CP015735	**MH067969**<br>**MH067971**<br>**MH067976**<br>**MH067977**				**MH067969**<br>**MH067970**<br>**MH067971**<br>**MH067977**	CP015733<br>**MH067967**<br>**MH067970**<br>**MH067971**<br>**MH067972**<br>**MH067973**<br>**MH067974**<br>**MH067976**<br>**MH067977**	
***Bacillus* (12)**	NZ_CP013056<br>NC_010180								
***Bosea* (1)**									
***Carnobacterium* (7)**						NC_022607	NC_022601<br>NC_022603<br>NC_022607<br>NZ_CP010819		
***Chryseobacterium* (2)**									
***Cryobacterium* (4)**	CP021994						CP016283	CP016284	
***Desulfotalea* (2)**	NC_006139								
***Exiguobacterium* (2)**									
***Flavobacterium* (2)**									
***Glaciecola* (1)**	CP002527						CP002527		
***Halocynthiibacter* (1)**		CP014328				CP014328			
***Halomonas* (2)**									
***Moraxella* (2)**									
***Octadecabacter* (4)**	NC_020909<br>NC_020910			NC_020910	NC_020909				

***Paenibacillus* (1)**									
***Pedobacter* (1)**									
***Photobacterium* (1)**									
***Planococcus* (24)**		CP025124<br>CM009124					CM009124<br>CP025124<br>CP025128	CP025123	
***Planomicrobium* (1)**									
***Polaromonas* (13)**	MG869625	MG869617<br>MG869618<br>MG869615<br>MG869620<br>MG869625	MG869620				MG869617<br>MG869618<br>MG869615<br>MG869616<br>MG869620		
***Pseudoalteromonas* (11)**	NZ_CP013139						CP011027	CP011027	
***Pseudomonas* (10)**	CP017887	NC_021212<br>NC_016644<br>CP017887<br>NZ_CP015601	NC_021212<br>NC_016644<br>NZ_CP015601					CP017887	
***Psychrobacter* (59)**	CP012708	NC_019278			CM009132	NC_021158	CM009116<br>CP012534<br>CM009109	NC_019274<br>NC_019278<br>CP000324<br>NC_009516<br>NC_021668<br>CP012711<br>CP012534<br>CM009136	
***Psychroflexus* (1)**									
***Runella* (5)**							NC_015704	NC_015694	
***Shewanella* (20)**		NC_017570<br>NC_017577<br>NC_009035	NC_009661<br>NC_009998<br>NC_009999<br>NC_016905		NC_017580		NC_009035	NC_017570<br>NC_017578<br>NC_017580<br>NC_009036<br>NC_011668<br>NC_016905	
***Sinorhizobium* (2)**					KU140623		NC_021209<br>KU140623		
***Sphingopyxis* (1)**									
***Streptomyces* (4)**	NZ_CP011665					NC_021056			NC_02105<br>6
***Sulfuricella* (1)**		NC_022358	NC_022358				NC_022358		
***Variovorax* (6)**									

	**Metabolism and transport of:**						**Energy production and conversion**
	**carbohydrates**	**amino acids**	**nucleotides**	**lipids**	**inorganic ions**	**toxic organic compounds**	
***Acinetobacter* (16)**		CP014653			CP014652<br>CP014653<br>CP014655<br>CP015111<br>CP019144<br>CP019145		
***Aeromonas* (4)**							
***Aliivibrio* (6)**							
***Altererythrobacter* (1)**	CP011453					CP011453	CP011453
***Alteromonas* (1)**	CP013927						
***Arthrobacter* (16)**	**MH067967**<br>**MH067969**<br>**MH067970**<br>**MH067971**<br>**MH067975**<br>**MH067976**<br>**MH067977**	**MH067970**<br>**MH067975**<br>**MH067976**	**MH067975**	**MH067967**<br>**MH067969**	**MH067971**<br>**MH067975**<br>**MH067976**<br>**MH067977**<br>CP015733	**MH067967**<br>**MH067976**	**MH067967**<br>**MH067971**<br>**MH067975**<br>**MH067976**
***Bacillus* (12)**		NC_010180					
***Bosea* (1)**	NZ_CP014302	NZ_CP014302					
***Carnobacterium* (7)**	NC_015390	NC_015390					
***Chryseobacterium* (2)**							
***Cryobacterium* (4)**					CP016283		
***Desulfotalea* (2)**		NC_006139					
***Exiguobacterium* (2)**							

***Flavobacterium* (2)**							
***Glaciecola* (1)**							CP002527
***Halocynthiibacter* (1)**							
***Halomonas* (2)**							
***Moraxella* (2)**							
***Octadecabacter* (4)**	NC_020910<br>NZ_CP012161	NC_020910	NC_020910	NC_020910			NC_020910
***Paenibacillus* (1)**							
***Pedobacter* (1)**							
***Photobacterium* (1)**							
***Planococcus* (24)**							CM009142
***Planomicrobium* (1)**							
***Polaromonas* (13)**	MG869615<br>MG869617<br>MG869616<br>MG869625	MG869615<br>MG869625		MG869620	MG869616	MG869617	
***Pseudoalteromonas* (11)**							NC_015708
***Pseudomonas* (10)**	NZ_CP015601				CP017887<br>NZ_CP015601	NC_016644<br>CP017887	NZ_CP015601
***Psychrobacter* (59)**					CM009139<br>CP022042<br>CM009133		
***Psychroflexus* (1)**							
***Runella* (5)**							NC_015693
***Shewanella* (20)**	NC_009035	NC_009035<br>NZ_CM009684			NC_009035		NC_009035
***Sinorhizobium* (2)**	KU140623	KU140623	KU140623	KU140623	KU140623		KU140623
***Sphingopyxis* (1)**							NC_008036
***Streptomyces* (4)**							
***Sulfuricella* (1)**							
***Variovorax* (6)**							

Accession numbers of plasmids identified and characterized in this study are bolded.
In parentheses numbers of plasmids found in cold-active representatives of a particular taxonomic group are indicated.

Descriptions of plasmids presented in the table:

CP015111 – plasmid unnamed1 (298,877 bp, *Acinetobacter* sp. TGL-Y2);
CP019144 – pmZS (198,391 bp, *Acinetobacter lwoffii* ZS207);
CP019145 – pZS-3 (13,816 bp, *Acinetobacter lwoffii* ZS207);
CP019151 – pZS-13 (6,168 bp, *Acinetobacter lwoffii* ZS207);
CP014652 – plasmid unnamed1 (141,965 bp, *Acinetobacter* sp. DUT-2);
CP014653 – plasmid unnamed2 (50,047 bp, *Acinetobacter* sp. DUT-2);
CP014655 – plasmid unnamed4 (11,158 bp, *Acinetobacter* sp. DUT-2);
CP015111 – plasmid unnamed1 (298,877 bp, *Acinetobacter* sp. TGL-Y2);
NC_011311 – pVSAL840 (83,540 bp, *Aliivibrio salmonicida* LFI1238);
CP011453 – plasmid unnamed (88,815 bp, *Altererythrobacter atlanticus* 26DY36);
CP013927 – pASTE61-200 (252,173 bp, *Alteromonas stellipolaris* LMG 21861);
CP015733 – plasmid unnamed1 (178,569 bp, *Arthrobacter* sp. U41);
CP015734 – plasmid unnamed2 (166,444 bp, *Arthrobacter* sp. U41);
CP015735 – plasmid unnamed3 (61,249 bp, *Arthrobacter* sp. U41);
NZ_CP013056 – pYWC2-8-1 (250,706 bp, *Bacillus thuringiensis* YWC2-8);
NC_010180 – pBWB401 (417,054 bp, *Bacillus weihenstephanensis* KBAB4);
NZ_CP014302 – plasmid (7,199 bp, *Bosea* sp. PAMC 26642);
NC_022607 – pWNCR15 (15,475 bp, *Carnobacterium inhibens* subsp. gilichinskyi WN1359);
NC_022601 – pWNCR12 (12,655 bp, *Carnobacterium inhibens* subsp. gilichinskyi WN1359);
NC_022603 – pWNCR64 (64,491 bp, *Carnobacterium inhibens* subsp. gilichinskyi WN1359);
NZ_CP010819 – pCP1 (8,883 bp, *Carnobacterium* sp. CP1);

NC_015390 – pCAR50 (50,105 bp, *Carnobacterium* sp. 17-4);
CP016283 – pP27867_1 (117,792 bp; *Cryobacterium arcticum* PAMC 27867);
CP016284 – pP27867_2 (58,936 bp, *Cryobacterium arcticum* PAMC 27867);
CP021994 – plasmid unnamed1 (169,719 bp, *Cryobacterium* sp. LW097);
NC_006139 – plasmid large (121,587 bp, *Desulfotalea psychrophila* LSv54);
CP002527 – pGLAAG01 (341,282 bp, *Glaciecola* sp. 4H-3-7+YE-5);
CP014328 – plasmid (46,566 bp, *Halocynthiibacter arcticus* PAMC 20958);
NC_020909 – pOA238_118 (118,287 bp, *Octadecabacter arcticus* 238);
NC_020910 – pOA238_160 (159,683 bp, *Octadecabacter arcticus* 238);
NZ_CP012161– plasmid PSB_p1 (31,560 bp, *Octadecabacter temperatus* SB1);
CM009124 – plasmid unnamed1 (70,671 bp, *Planococcus* sp. MB-3u-09);
CP025124 – plasmid unnamed1 (70,808 bp, *Planococcus* sp. MB-3u-03);
CP025128 – plasmid unnamed6 (5,115 bp, *Planococcus* sp. MB-3u-03);
CP025123 – plasmid unnamed12 (129,321 bp, *Planococcus* sp. MB-3u-03);
CM009142 – plasmid unnamed1 (152,323 bp, *Planococcus* sp. MB-3u-03);
MG869615 – pE10SP1 (86,294 bp, *Polaromonas* sp. E10S);
MG869616 – pE19SP1 (18,920 bp; *Polaromonas* sp. E19S);
MG869617 – pE3SP1 (101,077 bp, *Polaromonas* sp. E3S);
MG869618 – pE5SP1 (65,477 bp, *Polaromonas* sp. E5S);
MG869620 – pH6NP1 (82,545 bp, *Polaromonas* sp. H6N);
MG869625 – pW11NP2 (52,468 bp, *Polaromonas* sp. W11N);
NZ_CP013139 – pPBSW1 (906,103 bp, *Pseudoalteromonas* sp. Bsw20308);
CP011027 – plasmid unnamed (91,261 bp, *Pseudoalteromonas arctica* A 37-1-2)
NZ_CP015601 – pP27494_1 (135,475 bp, *Pseudomonas antarctica* PAMC 27494);
NC_015708 – pTML1 (97,600 bp, *Pseudoalteromonas* sp. SANK 73390);
NC_021212 – pGLE121P3 (39,583 bp, *Pseudomonas* sp. GLE121);
NC_016644 – plasmid KOPRI126573 (81,814 bp, *Pseudomonas* sp. MC1);
CP017887 – plasmid unnamed1 (371,069 bp, *Pseudomonas frederiksbergensis* ERDD5:01);
NC_019278 – pP60P2 (14,924 bp, *Psychrobacter* sp. DAB_AL60);
CM009132 – plasmid unnamed1 (14,711 bp, *Psychrobacter* sp. MES7-P7E);
NC_021158 – pKLH80 (14,835 bp, *Psychrobacter maritimus* MR29-12);
CM009116 – plasmid unnamed2 (59,859 bp, *Psychrobacter* sp. 4Dc);
CP012534 – pPspP11G5a (40,963 bp, *Psychrobacter* sp. P11G5);
CP012708 – plasmid 2 (15,605 bp, *Psychrobacter urativorans* R10.10B);
CP000324 – plasmid 1 (41,221 bp, *Psychrobacter cryohalolentis* K5);
CM009109 – plasmid unnamed1 (20,934 bp, *Psychrobacter* sp. 4Bb);
NC_019274 – pP62BP1 (34,467 bp, *Psychrobacter* sp. DAB_AL62B);
NC_009516 – pRWF101 (13,956 bp, *Psychrobacter* sp. PRwf-1);
NC_021668 – plasmid PsyG_26 (26,087 bp, *Psychrobacter* sp. G);
CP012711 – plasmid 5 (49,020 bp, *Psychrobacter urativorans* R10.10B);
CP022042 – plasmid unnamed1 (41,221 bp, *Psychrobacter cryohalolentis* FDAARGOS_308);
CM009133 – plasmid unnamed3 (7,765 bp, *Psychrobacter* sp. MES7-P7E);
CM009136 – plasmid unnamed1 (11,462 bp, *Psychrobacter* sp. Sarcosine-02u-2);
CM009139 – plasmid unnamed4 (6,971 bp, *Psychrobacter* sp. Sarcosine-02u-2);
NC_015693 – pRUNSL01 (106,999 bp, *Runella slithyformis* DSM 19594);
NC_015704 – pRUNSL02 (93,527 bp, *Runella slithyformis* DSM 19594);
NC_015694 – pRUNSL03 (66,926 bp, *Runella slithyformis* DSM 19594);
NC_017570 – pSBAL17501 (72392 bp, *Shewanella baltica* BA175);
NC_017577 – pSBAL11701 (120,153 bp, *Shewanella baltica* OS117);

NC_017580 – pSBAL11702 (91,728 bp, *Shewanella baltica* OS117);
NC_017578 – pSBAL11703 (71,173 bp, *Shewanella baltica* OS117);
NC_009035 – pSbal01 (116,763 bp, *Shewanella baltica* OS155);
NC_009036 – pSbal02 (74,000 bp, *Shewanella baltica* OS155);
NC_009661 – pS18501 (83,224 bp; *Shewanella baltica* OS155);
NC_009998 – pS19501 (75,605 bp, *Shewanella baltica* OS195);
NC_009999 – pS19502 (75,508 bp, *Shewanella baltica* OS195);
NC_011668 – pS22302 (65,448 bp, *Shewanella baltica* OS223);
NC_016905 – pSBAL67801 (80,698 bp, *Shewanella baltica* OS678);
NZ_CM009684 – pWMBT7 (113,604 bp; *Shewanella morhuae* CW7);
KU140623 – pSinB (297,376 bp, *Sinorhizobium* sp. M14);
NC_021209 – pSinA (108,938 bp, *Sinorhizobium* sp. M14);
NC_008036 – plasmid F (28,543 bp, *Sphingopyxis alaskensis* RB2256);
NZ_CP011665 – pSMg1-1 (529,571 bp, *Streptomyces* sp. Mg1);
NC_021056 – pSP01 (104,048 bp, *Streptomyces* sp. PAMC26508);
NC_022358– pSCD (86,619 bp, *Sulfuricella denitricans* skB26).
