# Supplementary material for: Insight Into the Diversity and Possible Role of Plasmids in the Adaptation of Psychrotolerant and Metalotolerant Arthrobacter spp. to Extreme Antarctic Environments
Source: Front Microbiol. 2018 Dec 18;9:3144. doi: 10.3389/fmicb.2018.03144 (PMC6305408; doi:10.3389/fmicb.2018.03144)
Supplement: Supplementary file 8 [file Data_Sheet_1.PDF]

Supplementary Material

Insight into the Diversity and Possible Role of Plasmids in the Adaptation of Psychrotolerant and Metalotolerant *Arthrobacter* spp. to Extreme Antarctic Environments

Krzysztof Romaniuk, Piotr Golec, Lukasz Dziewit*
*** Correspondence:** Dr. Lukasz Dziewit: ldziewit@biol.uw.edu.pl

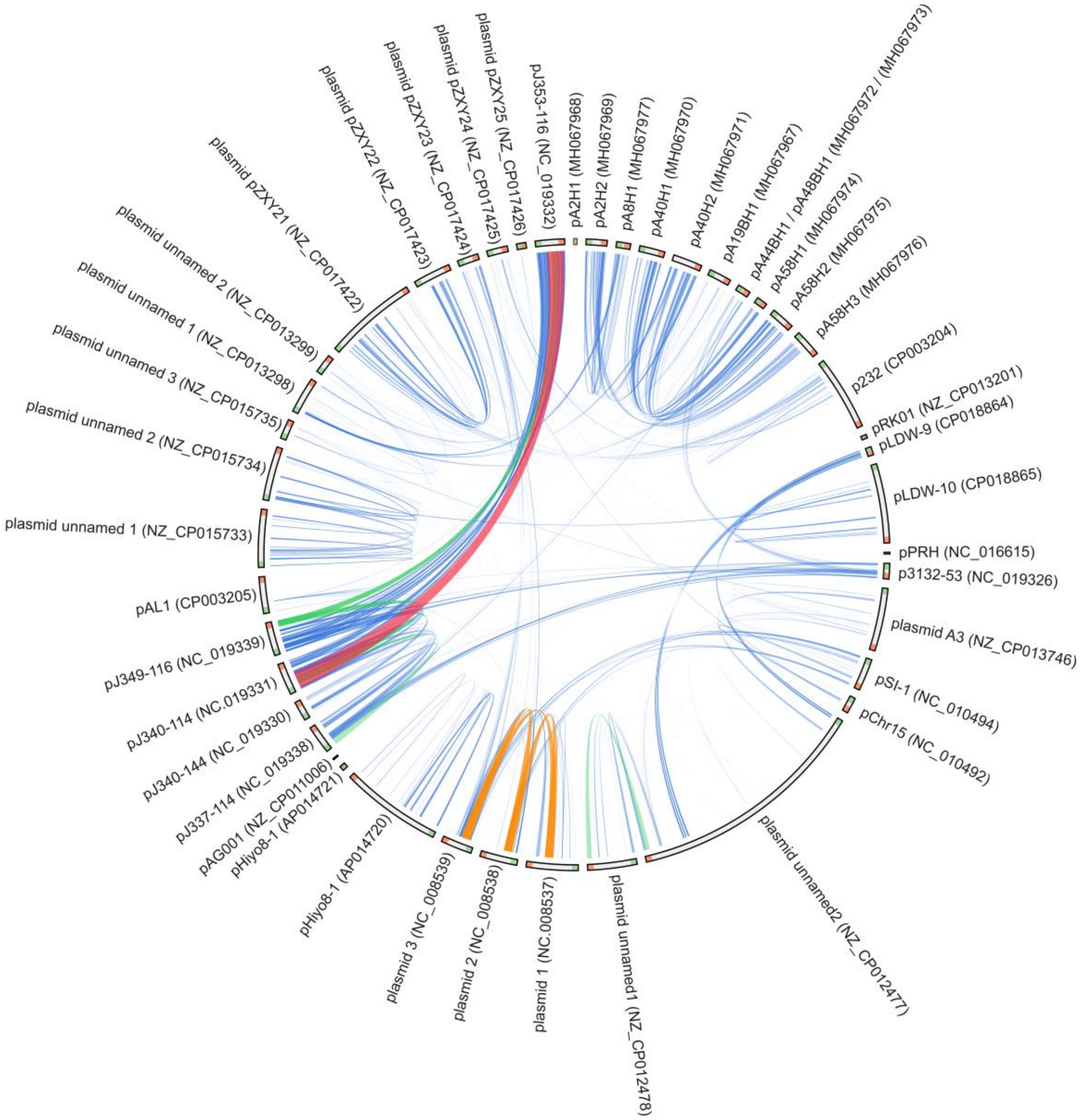

Figure S1. Comparison of the nucleotide sequences of the *Arthrobacter* plasmids. Accession numbers are presented in parentheses. The color ribbons connecting particular plasmids link similar DNA regions. Red and then yellow color represents the best much, while green and finally blue corresponds to the weakest similarity.
