# Supplementary material for: Insight Into the Diversity and Possible Role of Plasmids in the Adaptation of Psychrotolerant and Metalotolerant Arthrobacter spp. to Extreme Antarctic Environments
Source: Front Microbiol. 2018 Dec 18;9:3144. doi: 10.3389/fmicb.2018.03144 (PMC6305408; doi:10.3389/fmicb.2018.03144)
Supplement: Supplementary file 9 [file Data_Sheet_2.PDF]

Supplementary Material

Insight into the Diversity and Possible Role of Plasmids in the Adaptation of Psychrotolerant and Metalotolerant *Arthrobacter* spp. to Extreme Antarctic Environments

Krzysztof Romaniuk, Piotr Golec, Lukasz Dziewit*

*** Correspondence:** Dr. Lukasz Dziewit: ldziewit@biol.uw.edu.pl

	Protection against:			Cell mobility		Resistance to:		Protection against exogenous DNA	
	low temperature	oxidative stress	UV radiation	gas vesicles production	flagella formation	antibiotics	heavy metals	restriction-modification system	CRISPR-Cas system
Acinetobacter (16)	6.25	18.75			6.25		6.25	6.25	
Aeromonas (4)									
Aliivibrio (6)	16.67								
Altererythrobacter (1)		100.00							
Alteromonas (1)		100.00					100.00		
Arthrobacter (16)	6.25	6.25	25.00				25.00	56.25	
Bacillus (12)	16.67								
Bosea (1)									
Carnobacterium (7)						14.29	57.14		
Chryseobacterium (2)									
Cryobacterium (4)	25.00						25.00	25.00	
Desulfotalea (2)	50.00								
Exiguobacterium (2)									
Flavobacterium (2)									
Glaciecola (1)	100.00						100.00		
Halocynthiibacter (1)		100.00				100.00			
Halomonas (2)									
Moraxella (2)									
Octadecabacter (4)	50.00			25.00	25.00				
Paenibacillus (1)									
Pedobacter (1)									
Photobacterium (1)									
Planococcus (24)		8.33					12.50	4.17	
Planomicrobium (1)									
Polaromonas (13)	7.69	38.46	7.69				38.46		
Pseudoalteromonas (11)	9.09						9.09	9.09	
Pseudomonas (10)	10.00	40.00	30.00					10.00	
Psychrobacter (59)	1.69	1.69			1.69	1.69	5.08	13.56	
Psychroflexus (1)									
Runella (5)							20.00	20.00	
Shewanella (20)		15.00	20.00		5.00		5.00	30.00	
Sinorhizobium (2)					50.00		100.00		
Sphingopyxis (1)									
Streptomyces (4)	25.00					25.00			25.00
Sulfuricella (1)		100.00	100.00				100.00		
Variovorax (6)									
SUMMARY (247):	6.07	9.31	5.26	0.40	2.02	1.62	11.74	11.74	0.40

Figure S2. The proportion (%) of plasmids of cold-active bacteria that carry genes conferring protection against low temperature, oxidative stress, UV radiation and exogenous DNA, as well as cell mobility, and resistance to antibiotics and heavy metals. The numbers of plasmids found in cold-active representatives of a particular taxonomic group are indicated in parentheses. The values and pink blocks indicate the percentages of plasmids (from the pool of replicons of cold-active bacteria representing a given taxonomic group) that carry at least one gene conferring the particular physiological feature. The summarization was presented on a blue background. Detailed data are presented in table S5.
