# Supplementary material for: Insight Into the Diversity and Possible Role of Plasmids in the Adaptation of Psychrotolerant and Metalotolerant Arthrobacter spp. to Extreme Antarctic Environments
Source: Front Microbiol. 2018 Dec 18;9:3144. doi: 10.3389/fmicb.2018.03144 (PMC6305408; doi:10.3389/fmicb.2018.03144)
Supplement: Supplementary file 10 [file Data_Sheet_3.PDF]

Supplementary Material

Insight into the Diversity and Possible Role of Plasmids in the Adaptation of Psychrotolerant and Metalotolerant *Arthrobacter* spp. to Extreme Antarctic Environments

Krzysztof Romaniuk, Piotr Golec, Lukasz Dziewit*

*** Correspondence:** Dr. Lukasz Dziewit: ldziewit@biol.uw.edu.pl

	Metabolism and transport of:						Energy production and conversion
	carbohydrates	amino acids	nucleotides	lipids	inorganic ions	toxic organic compounds	
Acinetobacter (16)		6.25			37.50		
Aeromonas (4)							
Aliivibrio (6)							
Altererythrobacter (1)	100.00					100.00	100.00
Alteromonas (1)	100.00						
Arthrobacter (16)	43.75	18.75	6.25	12.50	31.25	12.50	25.00
Bacillus (12)		8.33					
Bosea (1)	100.00	100.00					
Carnobacterium (7)	14.29	14.29					
Chryseobacterium (2)							
Cryobacterium (4)					25.00		
Desulfotalea (2)		50.00					
Exiguobacterium (2)							
Flavobacterium (2)							
Glaciecola (1)							100.00
Halocynthiibacter (1)							
Halomonas (2)							
Moraxella (2)							
Octadecabacter (4)	50.00	25.00	25.00	25.00			25.00
Paenibacillus (1)							
Pedobacter (1)							
Photobacterium (1)							
Planococcus (24)							4.17
Planomicrobium (1)							
Polaromonas (13)	30.77	15.38		7.69	7.69	7.69	
Pseudoalteromonas (11)							9.09
Pseudomonas (10)	10.00				20.00	20.00	10.00
Psychrobacter (59)					5.08		
Psychroflexus (1)							
Runella (5)							20.00
Shewanella (20)	5.00	10.00			5.00		5.00
Sinorhizobium (2)	50.00	50.00	50.00	50.00	50.00		50.00
Sphingopyxis (1)							100.00
Streptomyces (4)							
Sulfuricella (1)							
Variovorax (6)							
SUMMARY (247):	8.10	5.67	1.21	2.02	8.10	2.43	5.67

Figure S3. The proportion (%) of plasmids of cold-active bacteria, that carry genes encoding proteins involved in metabolism and transport of carbohydrates, amino acids, nucleotides, lipids, inorganic ions, and toxic organic compounds, as well as energy production and conversion. The numbers of plasmids found in cold-active representatives of a particular taxonomic group are indicated in parentheses. The values and pink blocks indicate the percentages of plasmids (from the pool of replicons of cold-active bacteria representing a given taxonomic group) that carry at least one gene conferring the particular metabolic feature. The summarization was presented on a blue background. Detailed data are presented in table S5.
